# Synthesis, high-throughput screening and pharmacological characterization of β–lactam derivatives as TRPM8 antagonists

**DOI:** 10.1038/s41598-017-10913-x

**Published:** 2017-09-07

**Authors:** Roberto de la Torre-Martínez, M. Angeles Bonache, Pedro J. Llabrés-Campaner, Beatriz Balsera, Asia Fernández-Carvajal, Gregorio Fernández-Ballester, Antonio Ferrer-Montiel, M. Jesús Pérez de Vega, Rosario González-Muñiz

**Affiliations:** 1 Institute of Molecular and Cellular Biology, University Miguel Hernandez of Elche, 03202 Elche, Alicante Spain; 2 Institute of Medicinal Chemistry, IQM-CSIC, 28006 Madrid, Spain

## Abstract

The mammalian transient receptor potential melastatin channel 8 (TRPM8), highly expressed in trigeminal and dorsal root ganglia, mediates the cooling sensation and plays an important role in the cold hypersensitivity characteristic of some types of neuropathic pain, as well as in cancer. Consequently, the identification of selective and potent ligands for TRPM8 is of great interest. Here, a series of compounds, having a β-lactam central scaffold, were prepared to explore the pharmacophore requirements for TRPM8 modulation. Structure-activity studies indicate that the minimal requirements for potent β-lactam-based TRPM8 blockers are hydrophobic groups (benzyl preferentially or ^*t*^Bu) on R^1^, R^2^, R^3^ and R^5^ and a short N-alkyl chain (≤3 carbons). The best compounds in the focused library (**41** and **45**) showed IC_50_ values of 46 nM and 83 nM, respectively, in electrophysiology assays. These compounds selectively blocked all modalities of TRPM8 activation, i.e. menthol, voltage, and temperature. Molecular modelling studies using a homology model of TRPM8 identified two putative binding sites, involving networks of hydrophobic interactions, and suggesting a negative allosteric modulation through the stabilization of the closed state. Thus, these β-lactams provide a novel pharmacophore scaffold to evolve TRPM8 allosteric modulators to treat TRPM8 channel dysfunction.

## Introduction

Thermo-Transient Receptor Potential receptors (thermoTRPs) belong to a subfamily of ion channels responsible for the activation by changes in temperature, voltage, pressure, acid and different types of endogenous and exogenous chemicals. These channels are involved in thermosensation, from noxious cold (<15 °C) to harmful heat temperatures (>42 °C)^1^. Moreover, alterations on this family of ion channels contribute to the thermal hypersensitivity associated with pathological pain^2^. Beyond the implication in thermal transduction, their dysfunction has been associated with pathological processes, such as pulmonary diseases, neurodegeneration, skin sensitivity or cancer, among others^3^.

Transient Receptor Potential Melastatin 8 (TRPM8) is an ion channel from the thermoTRP family that was initially identified up regulated in prostate tumour cells^4^. While it is possible to find it in other tissues (bladder, lung or urogenital tract), TRPM8 is predominantly expressed in peripheral nervous system neurons^2, 5^. Different analysis in Dorsal Root Ganglia neurons (DRGs) showed that TRPM8 is expressed in sensory neurons, mostly in C and Aδ fibres. The functional TRPM8 receptor is a tetrameric membrane protein with four identical subunits assembled around a central aqueous pore. Each TRPM8 subunit shows a membrane domain composed of six transmembrane segments (S1–S6), with an amphipathic region between S5–S6 segments that forms the channel conductive pore, and intracellularly located amino- and carboxyl-termini^6^. TRPM8 channels are activated by menthol, icilin, voltage and cold temperatures (<26 °C in heterologous systems and <30 °C in sensory neurons) and, therefore, they can be considered as a molecular integrators of noxious stimuli in nociceptors^5, 7^. Moreover, TRPM8 knockout mice and pharmacological suppression of the TRPM8 activity markedly reduce the thermal hyperalgesia typical from some types of pain^8–10^. Notably, enhanced expression of this thermoTRP channel has been observed in various human chronic pathologies. In patients with peripheral nerve disease, innocuous cold stimulation elicits painful sensations^11, 12^. Similarly, some platinum-based chemotherapeutic agents, i.e. oxaliplatin, cause cold hypersensitivity, which severely restricts its dosage and the duration of treatment^13^. In addition, recent studies have demonstrated that the use of TRPM8 antagonists reduced the proliferation rates and proliferation fraction in all prostate tumour cells tested, but not in non-tumour cells^14^. Thus, TRPM8 has garnered great attention as a therapeutic target not only for pain management, but also for other pathologies^15^.

**Figure 1:**
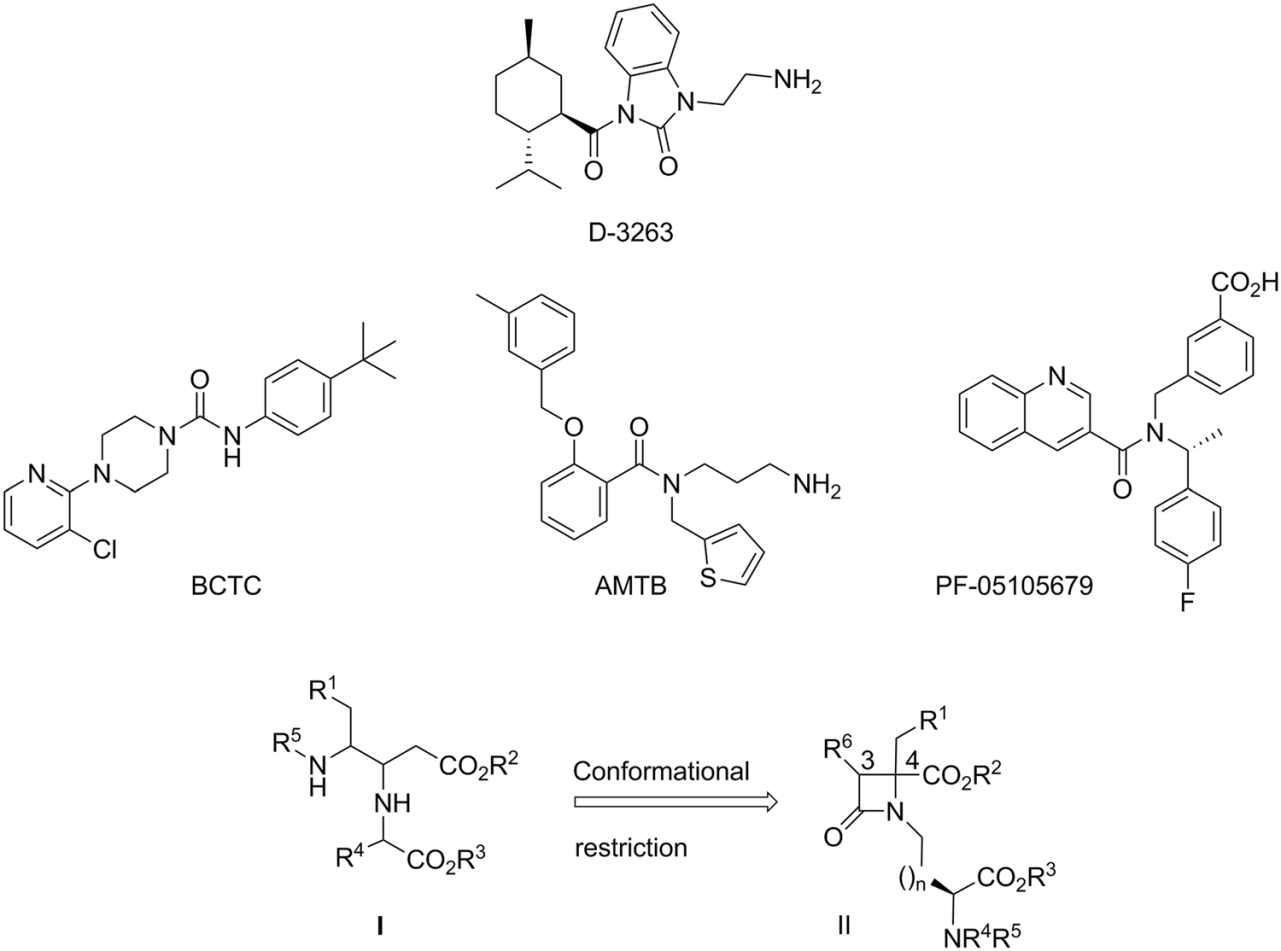
Advanced TRPM8 ligands and rational for the newly proposed modulators.

Accordingly, validation of TRPM8 as a therapeutic target has increased the efforts dedicated to design TRPM8 modulators, starting from different structural scaffolds. Thus, menthol and compounds with different hydrocarbon skeletons, earlier identified to possess menthol-like cooling effects, were then categorized as TRPM8 agonists^16, 17^. Among them, D-3263, entered phase I clinical trials for advanced prostate cancer and benign prostatic hyperplasia (Fig. 1)^18^. Among the TRPM8 antagonists, several families have been reported, including different chemotypes like benzothiophene, benzimidazole, and arylglycine as central scaffolds^15, 19–21^. Some of these compounds have demonstrated *in vitro* and *in vivo* activity in animal models of inflammatory and neuropathic pain. Furthermore, BCTC (Fig. 1) has been used together with other antagonists to reduce the proliferation of prostate tumour cells^14^. Moreover, some benzamide-type antagonists reduced the hyperactivity and painful sensation in bladder syndromes through inhibition of TRPM8^22^. A related isoquinoline derivative, PF-05105679, showed clinical efficacy in cold-related pain in humans^23, 24^. However, most of current TRPM8 inhibitors showed also agonistic/antagonistic properties towards other receptors and have side effects that justify the need for new, more selective compounds^25, 26^.

In a previous study we described some β,γ-diaminoesters **I** with TRPV1, TRPM8 and TRPA1 blocking properties (Fig. 1)^27^. Within this series, an increase in the general hydrophobicity of the molecule enhanced the ability to block the TRPM8 activation, allowing the identification of substituents and amino acid residues that led to selective modulators. For instance, compound **I** (R^1^, R^4^ = Bn, R^2^ = Me, R^3^ = ^*t*^Bu, R^5^ = Z) showed a 73% TRPM8 blockade at 5 μM, but reduced activity at TRPA1 and especially at TRPV1. Attaching these substituents on a rigid β-lactam scaffold^28^, could serve to decrease the inherent flexibility of the linear β,γ-diaminoester series, in a way to discover new TRP blockers with higher selectivity. On this basis, we designed an innovative chemical library founded on the β-lactam ring (structure II, Fig. 1). These new derivatives were evaluated by high-throughput screening allowing the study of the structure-activity relationship on this series, and contributing to the establishment of the minimal requirements for potent TRPM8 blockers. Hence, two potent TRPM8 blocking candidates were identified, which in electrophysiological assays revealed great selectivity against TRPM8 activity evoked by either chemical or physical stimuli. Computational studies suggested possible binding sites for these β-lactam-based TRPM8 modulators that contribute to our understanding of TRPM8 modulation.

## Results and Discussion

### Chemistry

**Figure 2:**
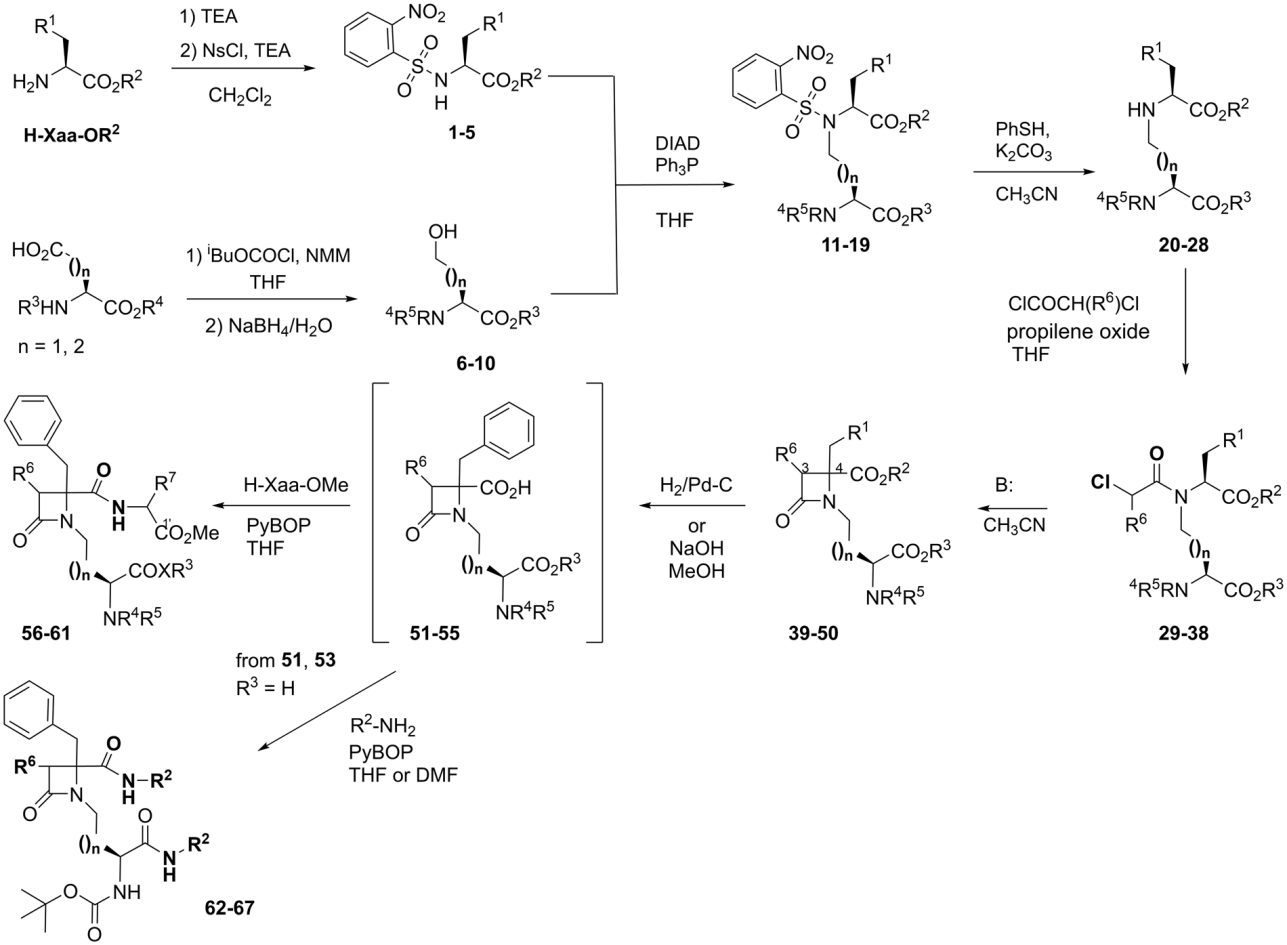
Synthesis of β–lactams from Phe or Ala and Asp or Glu derivatives, and dipeptide and amide analogues.

To mount three or four hydrophobic groups into a central scaffold, we designed highly functionalized β–lactam derivatives **II** (Fig. 1). Following our expertise in amino acid-derived 2-azetidinones^29, 30^ compounds **39–50** were prepared in a four-step synthetic procedure starting from N-nosyl-Phe- or N-nosyl-Ala-derived compounds **1–5** and alcohols **6–10** (Fig. 2), obtained from Asp and Glu derivatives. In the course of a Mitsunobu reaction^31^, these nosyl derivatives reacted with the alcohols to afford *N*-nosyl-*N*-alkyl amino acid derivatives **11–19** in good yield. Different esters (Me, ^*t*^Bu, Bn) and Z or Boc amino protecting groups were combined to obtain compounds with diverse hydrophobic character. Removal of the Ns group under standard conditions provided amino derivatives **20–28**, which upon reaction with chloroacetyl or racemic 2-chloropropanoyl chloride led to key linear synthetic intermediates **29–38**. Base-mediated cyclization of these chloroalkanoyl derivatives provided the desired β–lactam derivatives **39–50** in moderate to good yield. It is interesting to note that the cyclization of intermediate **29** afforded the expected compound **40** (56%), along with two related derivatives, resulting from the transesterification of the Me, Bn esters to Me, Me and Bn, Bn analogues (**39** and **41**, respectively). The preparation of **41**, one of the best compound within this series, as indicated later, was improved to 64% yield by direct cyclization of the dibenzyl ester linear intermediate **30**. All β–lactam derivatives were formed as mixtures of two diastereoisomers in variable ratio (Table 1S). The diastereoisomeric mixture was resolved by semipreparative chiral HPLC in the case of compound **41**, but was not attempted for other derivatives since similar blocking activity was observed for both isomers (see SI, Fig. S1, and discussion later).

To assign the configuration at C4 of the obtained diastereoisomers, we prepared and analyzed by ^1^H NMR a series of dipeptide derivatives **56–61** from compounds **41**, **43–46** (Fig. 2). The synthesis was carried out through hydrolysis or hydrogenolysis of one or two esters to the corresponding carboxylic acids **51–55**, followed by coupling with the corresponding amino acid, using PyBOP as coupling agent. Following established rules^32, 33^, for each diastereoisomeric pair, the dipeptide with more shielded chemical shift of the β-H aliphatic side-chain protons and higher retention time in HPLC was assigned as the heterochiral dipeptide (Table S2). These rules were previously validated for a closely related β–lactam derivative^34^. Thus, major diastereoisomers **56a** and **57a** were assigned as heterochiral around the 4,1′ positions, and therefore having 4*R*,1′*S*,1′′*S* configuration, as the coupled amino acids were of the natural series L. Minor isomers **56b** and **57b** have in concordance 4*S*,1′*S*,1′′*S* configuration. Although not separated by chromatography, the major components in compounds **58** and **61** are also heterochiral, showing more shielded Ala β-Me protons and longer retention time in HPLC than their corresponding minor diastereoisomers. In agreement with this, the main isomer in compound **59**, incorporating a D-Ala residue, is compatible with a homochiral derivative (considering the 4,1′ positions). These results mean that during the β–lactam ring closure the 4*R* isomers were predominantly formed, in contrast with that observed for simple L-Phe-derived β–lactams, which provided major 4*S* isomers due to memory of chirality^29, 35^. Compulsorily, this reversal selectivity should be attributed to the presence of the additional stereogenic centre (coming from the L-Asp or L-Glu residues) that should regulate the preferential formation of the 4*R* isomer.

The corresponding benzyl amide derivatives **62–64**, as well as some pyridine analogues **65–67** (which can be protonated), were also prepared from diacids **51** and **53** (Fig. 2, Table 3S). Diastereoisomeric pairs of compounds **63** and **67** were easily resolved by column chromatography. Based on the peptide derivatives assignment, 4*R* configuration was designated to major isomers.

### Biological evaluation

#### Screening of synthesized compounds by Ca^2+^-microfluorography

All compounds were tested at two different concentrations (50 and 5 µM) on TRPM8 and TRPV1 channels stably expressed in HEK and SH-SY5Y cell lines, respectively. The agonist induced intracellular Ca^2+^ signals were measured using a fluorescent Ca^2+^ indicator, in the absence and in the presence of test compounds. Menthol (TRPM8) and capsaicin (TRPV1) were used as the respective agonists. The obtained results were compared to those of **68** (AMTB, TRPM8 antagonist) and ruthenium red (TRPV1 antagonist). The IC_50_ values for the assay on TRPM8 were also calculated. The obtained results are summarized in Tables 1 and 2.

**Table 1:** Activity at TRPV1 and TRPM8 of β–lactam esters derived from Phe or Ala and Asp or Glu (ester derivatives).

**Compound**	**R** ^**1**^	**R** ^**2**^	**R** ^**3**^	**R** ^**4**^	**R** ^**5**^	**R** ^**6**^	**n**	**% Block TRPV1 50 µM**	**% Block TRPM8 50 µM**	**IC**_**50**_ **TRPM8 (μM)**
**39**	Ph	Me	Me	H	Boc	H	1	7.3 + 5.1	49.2 ± 15.3	50.2 ± 1.3
**40**	Ph	Me	Bn	H	Boc	H	1	42.4 + 5.3	75,35 ± 8.7	6.8 ± 1.02
**41**	Ph	Bn	Bn	H	Boc	H	1	38.3 + 17.9	96.3 ± 1.6	0.42 ± 1.62
**42**	Ph	Me	tBu	H	Z	H	1	51.7 + 15.4	82.2 ± 29.0	6.2 ± 1.6
**43**	H	Me	Bn	H	Boc	H	1	—	80.5 ± 15.8	36.8 ± 3.1
**44**	H	Me	tBu	H	Z	H	1	26.8 + 4.8	21.3 ± 9.8	339.8 ± 1.4
**45**	Ph	Bn	Bn	H	Boc	Me	1	31.8 + 6.5	100	0.8 ± 1.17
**46**	Ph	Bn	Bn	H	Boc	H	2	59.4 + 10.5	86.5±19.6	1.7 ± 2.7
**47**	Ph	tBu	Me	H	Z	H	2	49.8 + 7.2	94.9 ± 19.7	40.6 ± ± 1.3
**48**	Ph	tBu	Me	Me	Z	H	2	30.2 + 8.9	88.9 ± 13.1	7.9 ± 2.8
**49**	Ph	tBu	Me	Me	H	H	2	22.2 + 12.7	21.5 ± 20.5	750
**50**	H	tBu	Me	H	Z	H	2	27.2 + 32.7	4.1 ± 16.9	1800
**51**	Ph	H	H	H	Boc	H	1	15.9 ± 4.3	41.7 ± 8.7	74.1 ± 3.6
**53**	Ph	H	H	H	Boc	H	2	20.6 ± 9.2	40.9 ± 10.3	68.4 ± 8.5
**68**								—	100	7.0 ± 1.0
**Ruthenium red**								100	—	0.015 ± 0.0^a^

In our assay, the EC_50_ value of menthol was 136.6 μM (TRPM8) and that of capsaicin 1 μM (TRPV1). ^a^Evaluated in TRPV1.

**Table 2:** Percentage of blockade of TRPV1 and TRPM8 channels by peptide- and amide-derived β–lactams.

**Compound**	**R** ^**2**^	**X-R** ^**3**^	**R** ^**4**^	**R** ^**5**^	**R** ^**6**^	**n**	**% Block TRPV1 50 µM**	**% Block TRPM8 50 µM**	**IC**_**50**_ **TRPM8 (μM)**
**56a**	L-Phe-OMe	L-Phe-OMe	H	Boc	H	1	16.8 ± 10.4	99.3 ± 3.4	9.30 ± 1.0
**56b**	L-Phe- OMe	L-Phe-OMe	H	Boc	H	1	12.4 ± 10.8	99.3 ± 7.7	8.41 ± 1.4
**57a**	L-Ala-OMe	L-Ala-OMe	H	Boc	H	1	10.3 ± 11.9	33.7 ± 11.7	75.2 ± 1.9
**57b**	L-Ala-OMe	L-Ala-OMe	H	Boc	H	1	—	44.0 ± 16.5	70.4 ± 1.6
**58**	L-Ala-OMe	OBn	H	Z	H	2	18.3 ± 16.2	50.7 ± 9.5	48.41 ± 18.6
**59**	D-Ala-OMe	OMe	H	Z	H	2	19.4 ± 5.1	36.2 ± 22.3	81.2 ± 4.8
**60**	L-Ala-OMe	OMe	Me	Z	H	2	—	71.3 ± 18.8	32.6 ± 1.8
**61**	L-Ala-OMe	OMe	Me	H	H	2	—	78.3 ± 25.0	27.0 ± 1.7
**62a**	Bn	NHBn	H	Boc	H	1	42.5 ± 16.2	70.0 ± 18.6	7.1 ± 0.3
**63a**	Bn	NHBn	H	Boc	Me (R)	1	15.2 ± 11.8	100	5.7 ± 1.2
**63b**	Bn	NHBn	H	Boc	Me (S)	1	13.0 ± −9.0	100	8.3 ± 1.1
**64**	Bn	NHBn	H	Boc	H	2	26.9 ± 9.2	70.4 ± 15.2	11.5 ± 2.7
**65**	CH_2_(4-Py)	NHCH_2_(4-Py)	H	Boc	H	1	7.4 ± 9.5	39.3 ± 14.7	55.5 ± 2.3
**66**	(3-Py)	NH(3-Py)	H	Boc	H	1	22.8 ± 3.7	64.6 ± 13.2	5.7 ± 3.4
**67a**	(4-Py)	NH(4-Py)	H	Boc	H	1	36.3 ± 10.8	43.5 ± 9.8	38.13 ± 6.3
**67b**	(4-Py)	NH(4-Py)	H	Boc	H	1	31.7 ± 4.5	52.3 ± 7.6	7.26 ± 1.9

As shown in Table 1, the first three Phe-derived β–lactam compounds, **39**, **40** and **41** are structurally quite similar, sharing a phenyl group in R^1^, H at positions R^4^ and R^6^ and a *tert*-butoxycarbonyl group (Boc) as substituent R^5^. Differences among them are located at the ester moieties R^2^ and R^3^. According to the evaluation, the presence of benzyl esters both on R^2^ and R^3^ (**41**) confers to the molecule a high blocking effect against TRPM8 (95% at 5 µM), with an IC_50_ value in the low micromolar range. This activity decreases considerably when the R^2^ benzyl group was substituted by a small methyl group (**40**) or drastically when the methyl modification was done on both positions R^2^ and R^3^ (**39**). The importance of the two hydrophobic benzyl esters is reinforced with the results from derivative **42**. Similarly to **40**, it incorporates conservative hydrophobic substitutions on R^3^, with a *tert*-butyl (^*t*^Bu) group, and a benzyloxycarbonyl (Z) moiety on R^5^. The activity of **42** was slightly higher compare to **40**, 82% and 75% of TRPM8 blockade, respectively, but far from that of the potent **41**. Substituting the phenyl group of **40** and **42** by hydrogen (R^1^ = H) in **43** and **44** causes a dramatic decrease in activity, suggesting that an aromatic ring on this position is essential for the blocking effect. Regarding the R^6^ substituent, the incorporation of a methyl group at position C3 of compound **41**, to give analogue **45**, has little influence on the activity, because both compounds displayed similar potency.

To explore the relevance of the N-alkyl chain length for the activity we prepared and evaluated some Glu-derived compounds. Comparison of homologues **41** and **46**, with exactly the same substituents but different length of the N-alkyl chain (2 and 3 carbons, respectively), indicates that a small increment on the chain length reduces the blockade activity up to 4-fold. Glu derivative **47**, which maintains the 3 carbon chain but replaces the benzyl ester on R^3^ by a methyl group, while keeping conservative modification at R^2^ (^*t*^Bu instead Bn) and R^5^ (Z instead of Boc), showed poorer blockade of TRPM8 activity compared to **46**, in agreement with their lower homologues (**42** versus **41**). Compound **48**, the analogue of **47** that incorporates a small modification at R^4^ (a Me group instead of H), was slightly more effective (Table 1). The blocking activity of analogue **49** (R^5^ = H), resulting from the removal of the Z group in **48**, was almost inexistent, indicating a crucial importance of the R^5^ hydrophobic group. As above, no activity was observed for compound **50**, Ala analogues of **47**, corroborating the significance of the R^1^ phenyl group. Similarly, removal of both benzyl ester groups (R^2^, R^3^ = H) to the corresponding free carboxylic acids resulted in poorer active compounds (**51** and **53**).

Peptide derivatives, initially prepared to help in the configuration assignment of the parent compounds, were also tested for their ability to block TRPM8 and TRPV1 channels (Table 2). It is noteworthy that, in general, the substitution of the benzyl ester by amino acid residues on R^2^ and/or R^3^ leads to worst TRPM8 blockers, while keeping poor or inexistent activity at TRPV1. Thus, isomeric derivatives **56a** and **56b**, incorporating a big hydrophobic group (L-Phe-OMe) on R^2^ and R^3^ instead of the O-benzyl group, showed one order of magnitude reduced effect on TRPM8 respect to compound **41**. Although they satisfied the hydrophobic requirements on R^2^ and R^3^, the results might suggest that the overall size of these groups is also important, and perhaps that the presence of the methyloxycarbonyl moiety at the distal position is not well tolerated within the supposed hydrophobic pocket occupied by the Bn group. Additionally, the change of the ester oxygen (O) by an NH group might also have a negative influence in the activity, as confirmed for simple amide derivatives. As expected, the introduction of a less hydrophobic Ala residue on isomers **57a** and **57b**, small analogues of **56a** and **56b**, resulted in a strong reduction of the blocking activity. Similar outcomes were obtained with dipeptide derivatives **58**, **59** and **60**. Thus, substituting the R^2^ group of **47** and **48** by L-Ala-OMe (**58**, **60**) or D-Ala-OMe (**59**) led to a decrease of the activity in relation to parent compounds.

Eight derivatives were designed modifying the positions R^2^ and R^3^ to include an amide instead of an ester group, as a strategy to increase the metabolic stability of compounds within this series. Thus, compounds **62**–**64**, analogues of **41**, **45**, and **46**, where the benzyl esters on R^2^ and R^3^ were substituted by benzyl amides, were synthesized and evaluated. This O → NH change was detrimental for the activity, because it increased the IC_50_ value from 0.42 μM in **41** to 7.1 μM in **62**. Similarly, the introduction of benzyl amide residues on R^2^ and R^3^ reduced the activity of the parent diester **45** (IC_50_ = 0.8 μM), up to 8.1 μM for the *SSS*-stereoisomer **63b**, and in a lower extent to 5.7 μM for **63a**, having *RRS* configuration. This modification in the higher homologue **46** afforded derivative **64**, also with reduced activity compared to the diester and its shorter analogue **62**. It is well known that the bioisosteric change of a phenyl group by a pyridine moiety may serve to increase the aqueous solubility of compounds because it can be protonated. According to this, in an attempt to improve the solubility of these highly hydrophobic compounds, pyridine derivatives **65**, **66** and **67** were designed, synthesized from diacid **51**, and evaluated. The substitution of the benzyl group on R^2^ and R^3^ of **62** by a highly similar (4-pyridine)methyl moiety gave to compound **65**, showing a strong reduction of the activity compared to **62** and to the corresponding diester partner **41**. Interestingly, shorter analogues in which the benzyl group was directly substituted by either a 3-pyridine ring in **66** or a 4-pyridine moiety in **67a,b** recovered significant blockade activity, comparable to that of **62**. As expected, compound **66** showed improved solubility respect to **62** and **41** (>5- and >50-fold, respectively, see Table 4S in SI).

All together, these results support the premise that high TRPM8-blocking activity within this series requires hydrophobic moieties on R^1^, R^2^, R^3^ and R^5^ and a short N-alkyl chain, and also suggested that these compounds should interact with the receptor in a large binding pocket, able to accommodate different structures and sterereochemistries, and that the forces maintaining the interaction are probably mainly hydrophobic.

Regarding to the activity of these compounds on TRPV1, almost all of them were inactive over these channels (Tables 1 and 2), with only significant antagonist properties for a few compounds at the higher concentration evaluated (50 μM). No signs of agonist activity for this family of compounds were found in the TRP channels assayed (see Fig. 1S in SI).

#### Compounds **41** and **45** potently and selectively blocks TRPM8 activity

The initial screening and the SAR analysis identified Asp-derivatives **41** and **45** as the most potent TRPM8 channel blockers within the synthesized compounds. In addition, these β-lactam derivatives did not display apparent cytotoxicity in the MTT assay (see Fig. 2S in SI). The inhibitory activity of these candidates was further demonstrated electrophysiologically in HEK-CR1 expressing TRPM8 channel by patch clamp technique in whole cell configuration.

**Figure 3:**
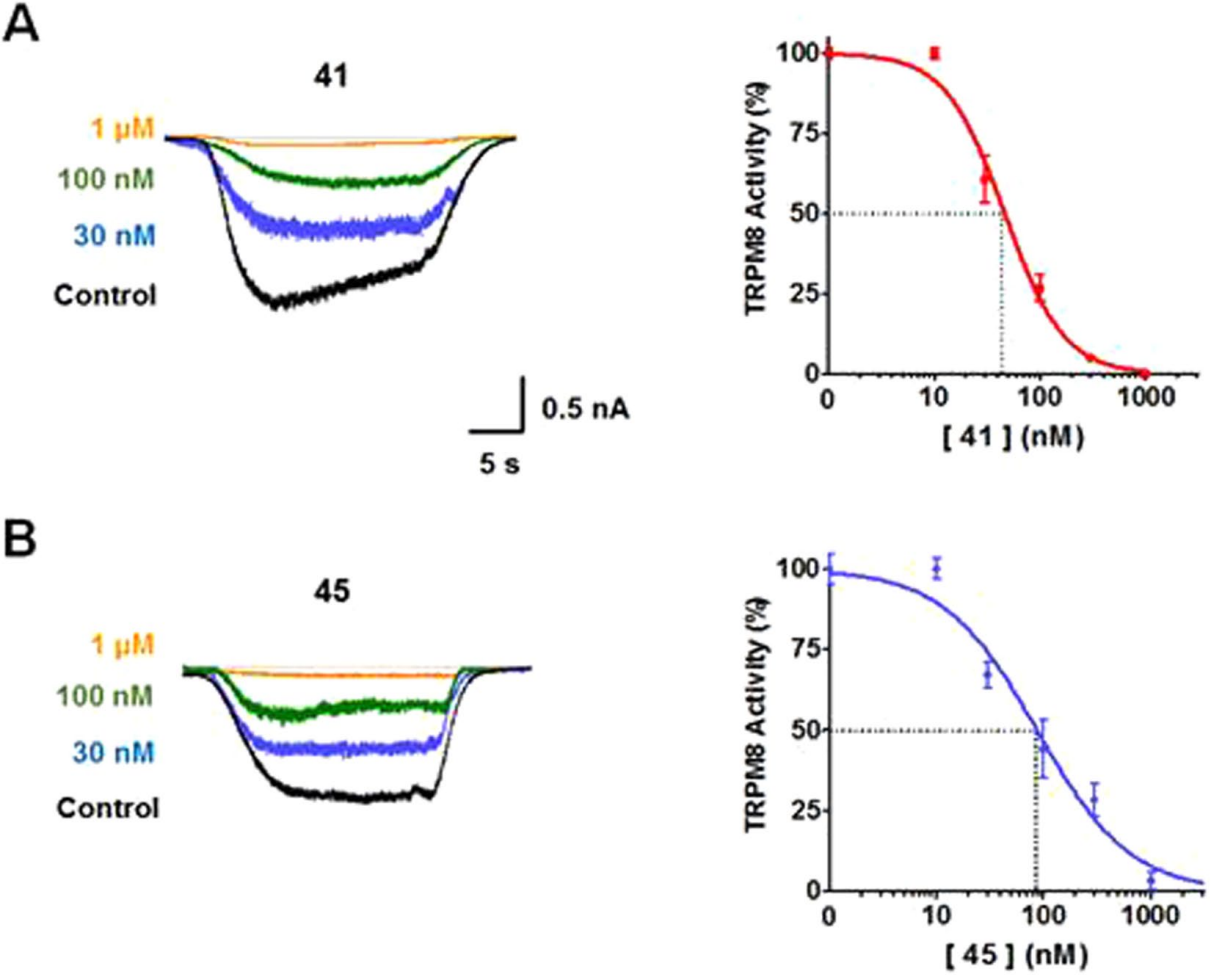
Compounds **41** and **45** strongly inhibited TRPM8-mediated currents. **Left**: Representative whole-cell voltage clamp recordings from TRPM8-expressing HEK-CR1 cells and compound **41** (**A**) and **45** (**B**). Voltage was held at −60 mV. Pre-application of compounds (20 s) was followed by co-application with 500 µM menthol (Control) for 20s. Current traces of different colours denote the different concentrations of compounds tested. **Right:** Dose response of compound **41** (**A**, red), **45** (**B**, blue) TRPM8 blocked activity. Solid line depicts the fitting to a Hill equation. Data are given as mean ± SEM, with n > 4 cells per data point.

As depicted in Fig. 3, perfusion with 500 µM menthol activated a large TRPM8 inward current at −60 mV (Fig. 3, control). The pre-application (20 s) of compound **41** (Fig. 3A) or **45** (Fig. 3B) at different concentrations, followed by co-application with 500 µM menthol, revealed a dose-dependent inhibition with almost totally absence of current at 1 µM in both compounds. The dose-response curves for compounds **41** and **45** were fit to obtain the IC_50_ values and the Hill coefficient. The extent of TRPM8 activity shown in Fig. 3 corresponds to the ratio of channel activity between the second (P2) and the first (P1) pulses (ratio P2/P1) of menthol in order to take into account the channel desensitization (supplementary information Fig. 3S)^36, 37^.

Against TRPM8 activity evoked by menthol, derivative **41** blocked potently the receptor, reporting an IC_50_ of 46 ± 6 nM with n_H_ of −1.5 ± 0.3 (Fig. 3A). The dose-response of the 3-methyl analogue **45** reported an IC_50_ of 83 ± 11 nM with n_H_ = −1.1 ± 0.2 (Fig. 3B), a slight decrease in the potency compared to compound **41** (Table 5S). Both compounds (**41** and **45**) were potent TRPM8 blockers, with activity in the nanomolar range, which position them within the most potent blockers of TRPM8 channel described to date.

Remarkably, co-application of separated diastereoisomers **41a** and **41b** (1 μM concentration) with 500 µM menthol resulted in a significant and quite similar reduction of current through the TRPM8 channel, slightly higher in the case of isomer **41a** (see supporting data, Fig. 4S). It seems that both isomers are able to tightly interact with the TRPM8 protein, suggesting a roomy binding pocket for the accommodation of the different hydrophobic substituents, in spite of their relative arrangement within each diastereoisomer.

As demonstrated by electrophysiology experiments, compound **41** bind to TRPM8 channels in a reversible manner (see Fig. 5S in SI). Four minutes after the compound has been removed from the bath, the channel can be activated again by menthol eliciting similar currents to prior compound application.

Patch clamp experiments in cells expressing hTRPV1, TRPA1, Kv1.1 and NaV1.6 indicated that compounds **41** and **45** exhibited high specificity and selectivity for TRPM8 channels (Table 5S in Supporting data).

#### Compounds **41** and **45** blocks all type of TRPM8 activation

TRPM8 is a polymodal channel, gated by chemical and physical stimuli. It has already been demonstrated that compounds **41** and **45** were able to block menthol-mediated TRPM8 activity. Hence, the possible inhibition of voltage- and cold-mediated TRPM8 activation was explored using patch clamp in whole cell configuration.

**Figure 4:**
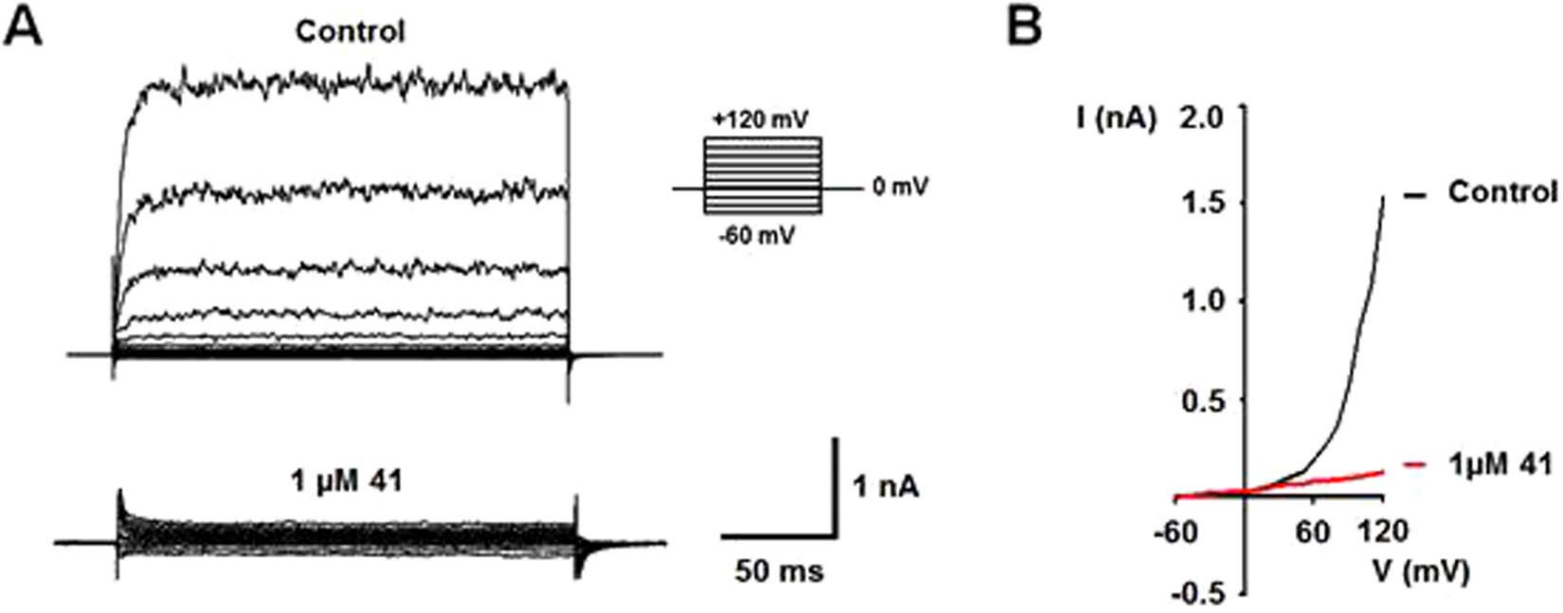
Compound **41** abolished the voltage-evoked response on TRPM8. (**A**) Representative families of TRPM8 ionic currents in absence (Control) or presence of 1 μM **41** elicited with a voltage step protocol consisting of 200-ms depolarizing pulses from −60 to +120 mV in steps of 20 mV (inset). Holding potential −60 mV, time between pulses = 5s. (**B)** I-V relationships of TRPM8 channel activity in the absence (black) and presence of 1 µM **41** (red).

In the absence of compound **41**, TRPM8 channel is activated by voltage steps, showing outward currents at positive voltages (Fig. 4A, top), which were notably reduced in the presence of 1 µM **41** (Fig. 4A, bottom). Figure 4B illustrates the current-voltage relationship (I-V) of voltage evoked TRPM8 response in absence of compound **41** (black), characterized by absence of current at negative potential and reversion near 0 mV to show strong outward rectifying currents at positive voltages. When 1 µM of **41** was applied (Fig. 4B, red), a strong reduction on the voltage-mediated TRPM8 activity at depolarizing voltages (+120 mV) was observed (90 ± 2%).

**Figure 5:**
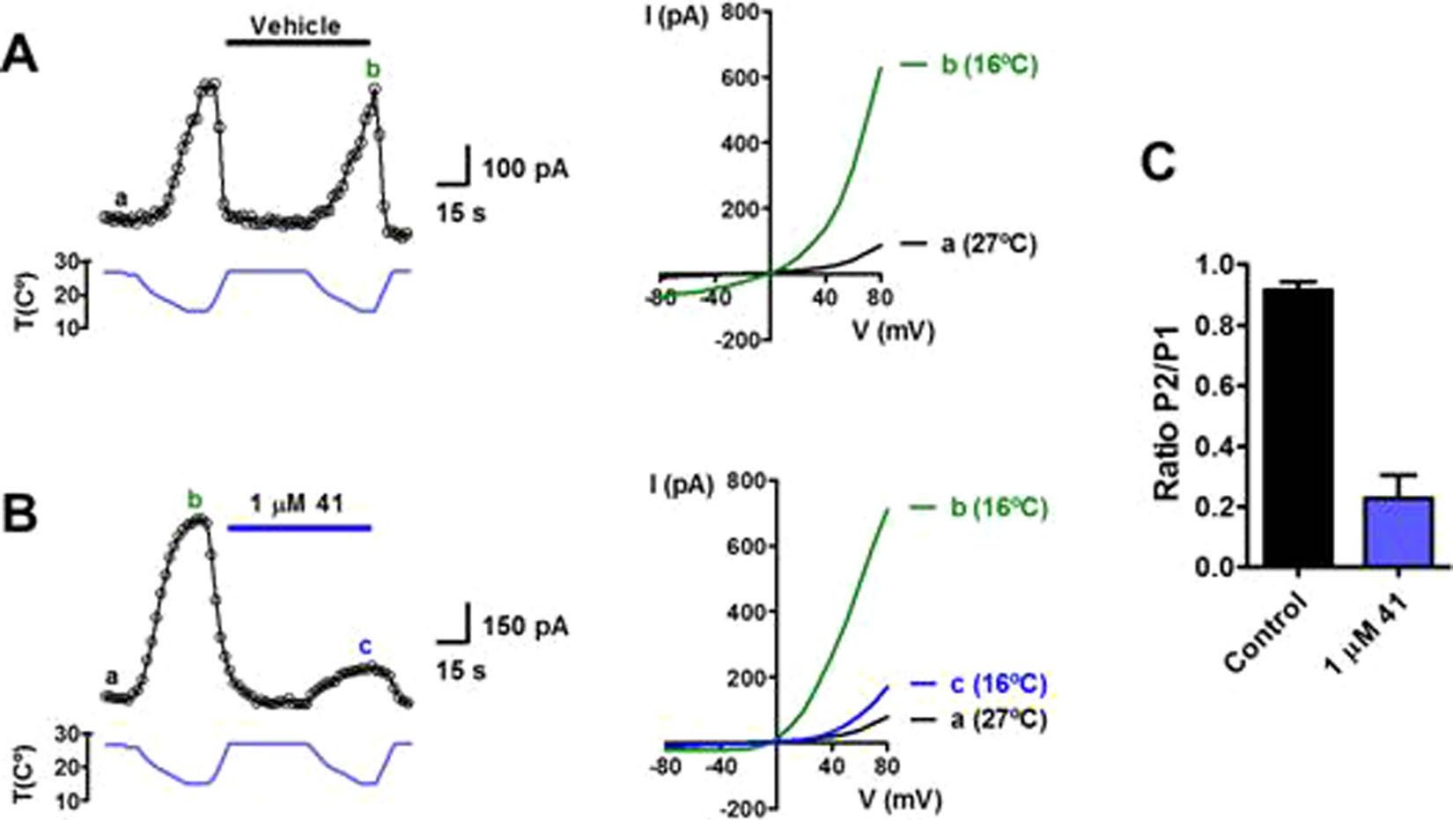
Compound **41** abolished the cold thermal activity response on TRPM8. Left: Representative families of TRPM8 ionic currents at +80mV in absence (**A**) or presence of 1µM **41** (**B**) activated by cold temperature (16 °C) obtained with a 300-ms ramp from −80 to +80 mV in intervals of 2 s during 3 minutes. Holding potential −60 mV. **Right:** I-V relationships of TRPM8 channel in basal conditions (a, black line), activated by cold in the absence (b, green line) and presence of 1 µM **41** (c, blue line). (**C**) Ratio currents between pulse 2 and pulse 1 induced by menthol in the absence (black bar) or in the presence (blue bar) of compound **41**.

It was also addressed the question of whether compound **41** might reduce the thermal activation of TRPM8, produced when mild cold is applied (Fig. 5). In absence of **41**, the application of two pulses of buffer at 16 °C evoked similar currents though the TRPM8 channel, and no desensitization processes were observed (Fig. 5A). In contrast, the application of β–lactam based compound **41** (Fig. 5B) at 1 µM together with the second thermal pulse revealed a significant decrease in the current intensity.

Similar results were found for the 3-Me analogue **45**, which was also able to reduce significantly the outward currents triggered by voltage and low temperature (see Fig. 6S in SI).

These results not only support the ability of compounds **41** and **45** to block the polymodal activation of TRPM8 channels, but also suggest that these compounds could be negative allosteric modulators, inhibiting the activation of the channel by interfering with its gating process.

#### Molecular modeling of the interaction between compound **41** and TRPM8 channel

Computational studies were performed to investigate the possible binding mode of compound **41** to TRPM8. Since the three-dimensional structure of TRPM8 has not been solved yet, a closed state, homology model of the TRPM8 was built, based on the recently published 3,4 Å-resolution structure of TRPV1^38^. The starting conformations of compound **41** (configuration *SSS*) were obtained by minimization. Then, series of docking simulations with the software implemented in Yasara^39, 40^, were ran to investigate whether the TRPM8 tetramer was able to bind the newly-synthesized small molecule, and to test the influence of this binding on the channel conformation. After 500 trials of simulations, the docking studies predicted two major solutions, site 1 and site 2 (Fig. 7S, SI).

**Figure 6:**
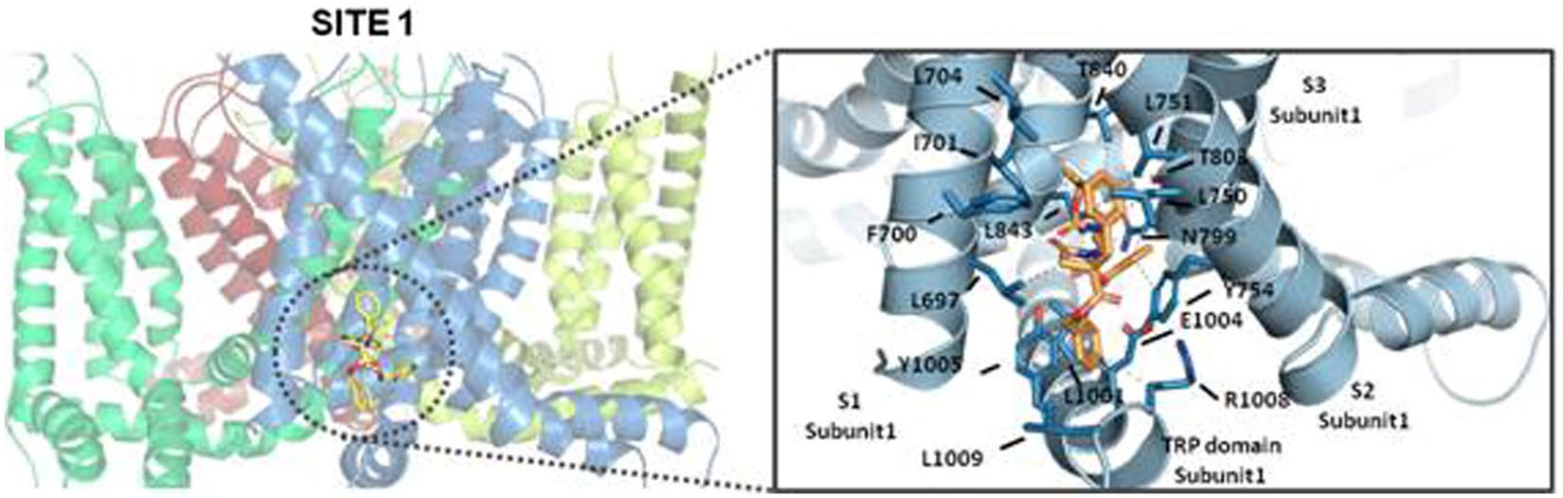
Binding sites 1 proposed for compound **41** into the TRPM8 receptor. Parts of the S1, S2 and S3 domains as well as the TRP domain of TRPM8 are depicted (right). The hydrophobic interactions between the compound and protein residues are represented as grey dots.

The most favourable in terms of energy was site 1 (binding energy 141.4 kcal/mol, Table 3), localized in a hydrophobic pocket formed by the transmembrane segments S1, S2, S3 and the TRP domain (Figs 6 and 8S). On this site, compound **41** displays a network of hydrophobic interactions with wide contact surface. Namely, the phenyl ring of the benzyl substituent R^1^ is surrounded by the side-chain of residues F700 and L704 of transmembrane helix S1, and L750 and L751 of helix S2. The phenyl ring of the 4-CO_2_Bn ester (R^2^) occupies a second pocket defined by L697 (S1), and L1009 and Y1005 of the TRP domain. In addition, the hydrocarbonated part of E1004 and R1008 side chains, both at the TRP domain, also contribute to this second hydrophobic pocket. Residue L697 establishes a hydrophobic interaction with the CH_2_ of the β–lactam ring. Two leucine residues of TRPM8, L843 (S4) and L1001 (TRP domain), the β–CH_2_ of N799, along with the phenol ring of Y754 contribute to fix the phenyl ring of the other benzyl ester (R^3^) within a third hydrophobic box. Finally, the position of the ^*t*^Bu group (R^5^) is stabilized by Van der Waals forces with I701 side-chain (S1), and γ–CH_3_ groups of T803 (S3) and T840 (S4).

**Table 3:** Theoretical binding energy for the interaction of β–lactam derivatives at the predicted binding sites of TRPM8, and for other TRP channels of known structure.

**Compound**	**Site**	**TRPM8**	**TRPV1**	**TRPA1**	**TRPV2**	**TRPV6**
**41**	**1**	141.4	101.2	65.7	33.0	29.2
	**2**	94.3	84.1	41.5	55.2	37.1
**45**	**1**	98.8	61.3	59.1	39.9	50.4
	**2**	92.2	37.7	25.2	42.6	22.2

Binding energy expressed as the energy needed to break away the complex (kcal/mol).

This site is different, but relatively close, to that occupied by agonists like menthol^41^, icilin^39^, and isoxazole derivatives^42^, as well as that proposed for the SKF96365 antagonist^43^, all of them having Y745 as a key interacting residue. However, it is very similar to that described for tryptamine-based ligands^44^, although in our case we did not find a π-π-stacking interaction with Y754. In agreement with tryptamine antagonists, our complex maintains H-bonds among Y754, E1004 and R1008 side-chains, which were disrupted upon binding of a tryptamine-based agonist.

The molecular modelling analysis also showed another energetically probable option, **site 2** (binding energy 94.3 kcal/mol, Table 3, Fig. 9S). On this solution, compound **41** was localized in a hydrophobic pocket formed by the transmembrane segments S3-S4-S5 from a subunit and the S6 from a contiguous monomer. The docked compound **41** exhibited hydrophobic interactions among the phenyl group of the R^1^ substituent and side-chains of F869, F872, and L873 residues (all situated on S5, subunit 1). Benzyl ester R^2^ establishes Van der Waals contacts with the indole ring of W798 (S3 of subunit 1), L864 and I865 side-chains, both located in S5 of subunit 1. Similarly, the phenyl ring of substituent R^3^ interacts with residues located at the S5 of subunit 1 (L864 and F868) and at the S6 of the contiguous monomer (I962 and L965, Fig. 8S). The ^*t*^Bu group of the Boc moiety shows close contacts with D802 (S3) and Y836 (S4) side-chains of subunit 1.

In order to determine which residues, among the suggested by docking studies, are more important for ligand binding, we have performed a computational alanine scanning of the key amino acids mentioned above for TRPM8 and compound **41** interaction. The mutations of individual amino acids followed by binding energy determinations showed that residues Y754, L697 and E1004 are the most important ones for the binding of compound **41** to site 1. In the case of site 2, the *in silico* mutagenesis studies revealed that W798, Y836 and F869 were essential residues for binding of compound **41** (See Table 6S in SI).

Once determined these important residues in TRPM8 as part of **41** binding site, we located the equivalent residues in other TRP channels, as determined by superposition of their structures. The results are shown in Table 7S. Side chain differences indicated important changes in the residues from site 1 and 2. For instance, in site 1 the aromatic Y754 of TRPM8 become a positively charged amino acid in TRPV1 and TRPA1; the hydrophobic L697 become polar in TRPV1, TRPA1 and TRPV2, and the negative charge E1004 become hydrophobic in TRPA1 or TRPV6. Similarly, the aromaticity of site 2 in TRPM8 disappears in two of the three positions tested in all TRPs.

To further predict the selectivity of compound **41** and **45** for TRPM8 versus TRPV1, we also performed computational studies on TRPV1 (see supporting information for details, Figs 10S and 11S). The TRPV1 sites (1 and 2) are larger and more open than in TRPM8. Accordingly the network of hydrophobic interactions is significantly reduced, especially in site 1. As a result, the theoretical binding energy calculated for both sites of TRPV1 is notably lower than that found for TRPM8 (Table 3). The hydrogen bond between the ester oxygen of the 3-CO_2_Bn and the side-chain NH of N57 (S1) could account for the higher binding energy of **41** at TRPV1 site 1 respect to that of site 2, regardless of the lower number of hydrophobic interactions in the first case. Similar results were observed for other TRP channels, such as TRPA1, TRPV2 and TRPV6 (Table 3). The binding energies of both compounds in sites 1 and 2 were lower than those obtained for TRPM8, indicating a marked preference for TRPM8. It is observed a notably low binding energy of derivatives **41** and **45** for TRPV2 and TRPV6.

The high number of hydrophobic interactions found in the energetically most favourable solution (TRPM8, site 1) could explain the good affinity of the molecule for the receptor and the high potency of compound **41** (Fig. 6). We propose that the physical proximity of this hypothetical binding site to the TRP box region and the interactions with the S1-S2-S3-S4-TRP domain could preserve the closed state of the channel. Moreover, it has been reported that structural changes in the S2 helix can severely affect the activity of the channel^45^. Regarding the importance of the TRP domain, recent papers have remarked the central role of this region in the gating mechanism of TRPM8 channels. Thus, activating stimuli seems to disrupt interactions in the proximal 980–992 region of the TRP domain, favouring channel opening^46^. Interestingly, other TRP channel such as TRPV1 or TRPV4 also showed a similar gating mechanism, suggesting a conserved function for this region^47^. Furthermore, the SAR results (Tables 1 and 2) revealed that substitutions on R^1^ (**44**, **45**, **50**), and on R^2^, R^3^ (**39**, **51**, **62**, **65**) that reduced the hydrophobic character of compounds, and hence the number of hydrophobic interactions, diminished the activity of these compounds. Altogether, these results support the hypothesis of a stabilization of the closed state of TRPM8 by the highly hydrophobic β–lactam analogues on site 1.

The recently reported structure of TRPV1 solved by cryo-electron microscopy describes an electron density assigned to capsaicin located pocket between helices S3 and S4^38^, in agreement with previous reports on vanilloid binding^48, 49^. Moreover, it has been reported that capsaicin contact with S4-S5 and S6 from an adjacent subunit produces a structural rearrangement that stabilized the TRPV1 channel in the open state^50^. We hypothesized that compound **41** in site 2 (Fig. 8S), could be acting on TRPM8 in a similar way as capsaicin in TRPV1. Despite the fact that capsaicin is an agonist of TRPV1 channel and **41** is a selective TRPM8 blocker, these differences suggest that the binding pocket of capsaicin could be a conserved site for regulation of TRP channels. In fact, the synthetic analogue of capsaicin, capsazepine^51^, a competitive antagonist of capsaicin, support this hypothesis, where different interactions in the same binding pocket formed by S3-S4-S5 and S6 from an adjacent subunit are able to confer agonist or antagonist effect to ligands.

Taken together, these computational studies on the TRPM8 channel suggest the existence of more than one binding site where ligands can exert their negative allosteric modulation, although for compound **41** the difference in binding energy indicates site 1 as the most probable interaction pocket.

## Conclusions

In conclusion, we showed that compounds **41** and **45** are strong and selective TRPM8 blockers and represent the first potent members of a novel class of TRP channels modulators based on a privileged β-lactam scaffold. This central core, traditionally used in the synthesis of antibiotics, is nowadays earning new and promising therapeutic applications. Unlike most of the TRPM8 antagonists described to date, compounds **41** and **45** have revealed an important selectivity versus other TRP members as well as other ion channels. Functionally, compounds **41** and **45** are TRPM8 modulators that block all modes of activation by a putative negative allosteric modulation. Computational studies with **41** suggest two possible binding sites for these β-lactam derivatives. These drug binding sites seems to be conserved among thermoTRP channels, although they differ in the network of potential interactions, thus providing new opportunities to design and develop better receptor blockers for the thermoTRP channel family. Because of its potency and selectivity, together with the simplicity of its synthesis, compounds **41** and **45** represent new hits for TRPM8 modulation, which are now being further modified to discover new analogues with improved properties for prospective therapeutic application in pain and cancer. Future *in vivo* studies with ameliorated compounds will unveil the therapeutic potential of these novel TRPM8 blockers, either for systemic or topical applications.

## Methods

### Chemistry

For experimental details and description of all new synthetic intermediates, and the characterization of most β–lactam derivatives see the supporting data.

#### Synthesis of 2-azetidinones

BTPP (2.4 mmol, 0.75 mL) or Cs_2_CO_3_ (3.2 mmol, 1.04g) was added to a solution of *N*-alkyl-*N*-chloroalkanoyl derivatives **29–38** (1.6 mmol) in dry CH_3_CN (4 mL), under Ar atmosphere. The reaction mixture was stirred until the starting material disappeared. The solvent was removed and the residue was extracted with EtOAc, and washed with H_2_O and brine, successively. Finally, the organic phase was dried over Na_2_SO_4_, filtered, and concentrated. The residue was purified by flash chromatography on silica gel, using the eluent mixture indicated in each case.

4*R,S*-Benzyl-4-benzyloxycarbonyl-1-[(3′*S*-benzyloxycarbonyl*-3-tert*-butoxycarbonylamino-3′-benzyloxycarbonyl)prop-1′-yl]-2-oxoazetidine (**41**). Syrup. Yield: 4% (from **29**, B:BTPP), 64 % (from **30**, B:BTPP). Eluent: EtOAc:Hexane (1:1). HPLC: t_R_ = 17.22 min (gradient of 5% to 100% of A, in 20 min). HPLC (Chiral C column): t_R_ = 20.15, 22.18 min (Isocratic 9/91 (Acetone/Hexane). Diastereoisomers ratio M, m = 53:47 from **29** (86:14, from **30**). Diastereoisomers were separated by semipreparative HPLC (Chiral C column). Eluent: Acetone:Hexane, 2:98, Isomer 4*S* t_R_ = 9.78 min, Isomer 4*R* t_R_ = 10.75 min.

Isomer 4*R* (**41a**). Syrup. HPLC: t_R_ = 17.22 min (gradient of 5% to 100% of A, in 20 min). [α_D_] = +4.82 ^1^H NMR (400 MHz, CDCl_3_): δ 7.35–7.02 (m, 15H, Ar), 5.17 (d, 1H, *J* = 12.4 Hz, OCH_2_), 5.16 (s, 2H, OCH_2_), 5.11 (d, 1H, *J* = 12.4 Hz, OCH_2_), 5.01 (m, 1H, 3-NH), 4.25 (m, 1H, 3′-H), 3.25(d, 1H, *J* = 14.2 Hz, 4-CH_2_), 3.24 (m, 2H, 1′-H), 3.15 (d, 1H, *J* = 14.8 Hz, 3-H), 3.14 (d, 1H, *J* = 14.2 Hz, 4-CH_2_), 2.88 (d, 1H, *J* = 14.8 Hz, 3-H), 2.18 (m, 1H, 2′-H), 1.97 (m, 1H, 2′-H), 1.44 (s, 9H, CH_3_^*t*^Bu).^13^C NMR (75 MHz, CDCl_3_): 172.0 (COO), 171.2 (COO), 166.5 (CON), 155.6 (OCON), 135.5, 134.94, 134.5, 129.8, 129.0, 128.9, 128.8, 128.7, 128.5, 127.7 (C Ar), 80.3 (C ^*t*^Bu), 67.9, 67.5 (OCH_2_), 63.3 (C4), 52.1 (C3′), 46.0 (C3), 40.1 (C1′), 39.0 (4-CH_2_), 31.1 (C2′), 28.5 (CH_3_^*t*^Bu). MS (ES)^+^: 587.46 [M+H]^+^. Exact Mass calculated for C_34_H_38_N_2_O_7_: 586.26790, found: 586.26869.

Isomer 4*S* (**41b**). Syrup. HPLC: t_R_ = 17.22 min (gradient of 5% to 100% of A, in 20 min). [α_D_] = +103.65 ^1^H NMR (400 MHz, CDCl_3_): δ 7.35–7.03 (m, 15H, Ar), 5.14 (s, 4H, OCH_2_), 5.03 (d, 1H, *J* = 6.6 Hz, 3-NH), 4.21 (m, 1H, 3′-H), 3.25 (d, 1H, *J* = 14.0 Hz, 4-CH_2_), 3.14 (d, 1H, *J* = 14.8 Hz, 3-H), 3.13 (m, 3H, 1′-H, 4-CH_2_), 2.86 (d, 1H, *J* = 14.8 Hz, 3-H), 2.20 (m, 1H, 2′-H), 1.92 (m, 1H, 2′-H), 1.44 (s, 9H, CH_3_^*t*^Bu). ^13^C NMR (75 MHz, CDCl_3_): 171.9 (COO), 171.2 (COO), 166.4 (CON), 155.9 (OCON), 135.4, 134.8, 134.4, 129.8, 129.0, 128.9, 128.7, 128.6, 128.5, 127.6 (Ar), 80.2 (C ^*t*^Bu), 67.8, 67.4 (OCH_2_), 63.1 (C4), 51.9 (C3′), 45.8 (C3), 39.8 (C1′), 38.9 (4-CH_2_), 30.8 (C2′), 28.4 (CH_3_^*t*^Bu). MS (ES)^+^: 587.46 [M+H]^+^. Exact Mass calculated for C_34_H_38_N_2_O_7_: 586.26790, found: 586.26866.

4*R,S*-Benzyl-4-benzyloxycarbonyl-1-[(3′*S*-benzyloxycarbonyl-3′-*tert*-butoxycarbonylamino)prop-1′-yl]-(3*R,S)*-methyl-2-oxoazetidine (**45**). Syrup. Yield: 61% (from **34**, B:BTPP). Eluent: EtOAc:Hexane (1:2). HPLC: t_R_ = 17.58 min (gradient of 5% to 100% of A, in 20 min). Diastereoisomers ratio M, m = 58:42.^1^H NMR (400 MHz, CDCl_3_): δ 7.37–7.04 (m, 15H, Ar), 5.22 (s, 2H, OCH_2_), 5.12 (s, 2H, OCH_2_), 5.03 (d, 1H, *J* = 6.6 Hz, 3-NH, M), 4.98 (d, 1H, *J* = 6.6 Hz, 3-NH, m), 4.14 (m, 1H, 3′-H), 3.51 (d, 1H, *J* = 15.1 Hz, 4-CH_2_), 3.45 (d, 1H, *J* = 14.5 Hz, 4-CH_2_), 3.03 (m, 4H, 4-CH_2,_ 1′-H, 3-H), 2.24 (m, 1H, 2′-H), 1.97 (m, 1H, 2′-H), 1.43 (s, 9H, CH_3_^*t*^Bu), 1.08 (d, 3H, *J* = 7.6 Hz, 3-CH_3,_ M), 1.07 (d, 3H, *J* = 7.6 Hz, 3-CH_3,_ m). ^13^C NMR (75 MHz, CDCl_3_): 172.0 (COO), 171.2 (COO), 171.1 (COO), 169.7 (CON), 169.6 (CON), 155.5 (OCON), 135.5, 135.0, 134.9, 134.8, 129.8, 129.15, 129.1, 129.00, 128.95, 128.9, 128.8, 128.6, 128.5, 128.4, 128.3, 127.7, 127.6 (Ar), 79.9 (C ^*t*^Bu), 69.0 (C4), 67.7 (C4), 67.2, 67.1 (OCH_2_), 54.0 (C3), 53.8 (C3), 52.1 (C3′), 40.9 (4-CH_2_), 40.7 (4-CH_2_), 40.1 (C1′), 40.0 (C1′), 30.8 (C2′), 30.7 (C2′), 28.4 (CH_3_^*t*^Bu), 10.4 (3-CH_3_), 10.3 (3-CH_3_). MS (ES)^+^: 601.52 [M+H]^+^. Exact Mass calculated for C_35_H_40_N_2_O_7_: 600.28355, found: 600.28432.

#### Synthesis of dipeptide and amide derivatives

A solution of the corresponding 4-carboxy β–lactam derivative (0.33 mmol) and the corresponding amino acid derivative or amine (0.66 or 0.33 mmol) in dry THF (4 mL) was successively treated with PyBOP (0.66 mmol, 0.34g) and TEA (0.18 mL, 1.32 mmol) at room temperature. The stirring was continued until complete disappearance of the starting material (1–2 days). The isomers ratio was determined by HPLC on the crude reaction mixtures. To characterize the obtained peptide or amide derivatives, the solvent was evaporated, and the residue was dissolved in EtOAc and washed with citric acid (10%), NaHCO_3_ (10%), and brine. The organic layer was dried (Na_2_SO_4_) and evaporated, leaving a residue which was purified on a silica gel column or by semipreparative HPLC, as specified in each case (see supporting data).

### Biological evaluation

#### High-throughput screening with calcium microfluorography

For fluorescence assays, cells expressing TRP channels (SH-SY5Y-TRPV1 and HEKCR1-TRPM8) were seeded in 96-well plates (Corning Incorporated, Corning, NY) at a cell density of 40,000 cells 2 days before treatment. The day of treatment the medium was replaced with 100 µL of the dye loading solution Fluo-4 NW supplemented with probenecid 2.5 mM. Then the compounds dissolved in DMSO were added at the desired concentrations and the plate(s) were incubated at 37 °C in a humidified atmosphere of 5% CO_2_ for 60 minutes.

The fluorescence was measured using instrument settings appropriate for excitation at 485 nm and emission at 535 nm (POLARstar Omega BMG LABtech). A baseline recording of 4 cycles was recorded prior to stimulation with the agonist (10 µM capsaicin for TRPV1 and 100 µM menthol for TRPM8). The corresponding antagonist

(10 µM Ruthenium Red forTRPV1 and 10 µM **68** for TRPM8) was added for the blockade. The changes in fluorescence intensity were recorded during 15 cycles more. DMSO, at the higher concentration used in the experiment, was added to the control wells. The blocking percentage was calculated as follow (Eq. 1):

1 $${\boldsymbol{ \% }}\,{\boldsymbol{TRP}}\,{\boldsymbol{Blocked}}\,{\boldsymbol{Response}}=(1-\frac{Fo-Fi}{FCo-FCi})\ast 100$$

where **Fo** is the fluorescence after the addition of menthol in the presence of the compound, **Fi** is the fluorescence before the addition of menthol in the presence of the compound, **FCo** is the fluorescence after the addition of menthol in the absence of the compound, **FCi** is the fluorescence before the addition of menthol in the absence of the compound. To guarantee that our results were useful we only used assays with a Z-factor higher than 0.5.

The statistical Z-factor to determine the quality of the HTS experiment as was calculated using the equation 2:

2 $${\boldsymbol{Z}} \mbox{-} {\boldsymbol{factor}}=1-\frac{3\ast (S{D}_{max}+S{D}_{{\rm{\min }}})}{Mea{n}_{max}-Mea{n}_{min}}$$

where: Mean_max_ is the mean of the maximum fluorescence in the presence of agonist, SD_max_ is the standard deviation of the maximum fluorescence in the presence of agonists, Mean_min_ is the mean of the maximum fluorescence in the presence of agonist and antagonist and SD_min_ is the standard deviation of the maximum fluorescence in the presence of agonist and antagonist.

#### Patch clamp electrophysiology

Electrophysiological recording was carried out 1–3 d after cells seeded. Membrane currents and voltages were recorded by patch clamp using the whole-cell configuration. For whole-cell recordings of HEK-hTRPV1 and HEK-TRPM8 cells, pipette solution contained (in mM) 140 CsCl, 5 EGTA, and 10 HEPES, adjusted to pH 7.2 with CsOH, and bath solution contained (in mM) 140 NaCl, 5 KCl, 2 CaCl_2_, 2 MgCl_2_, 5 EGTA, 10 D-glucose, 10 Mannitol and 10 HEPES, adjusted to pH 7.4 with NaOH. In calcium-free bath solution, CaCl_2_ was replaced with 5mM EGTA. For potassium and sodium recordings, CsCl was substituted by KCl and the pH was adjusted to pH 7.2 with KOH. Patch pipettes were prepared from thin-walled borosilicate glass capillaries (World Precision Instruments, Sarasota, FL, USA), pulled with a horizontal puller (P-97, Sutter Instruments, Novato, CA, USA) to have a tip resistance of 2–4 MΩ when filled with internal solutions. Data were sampled at 10 kHz (EPC10 amplifier with PatchMaster 2.53 software, HEKA Electronics, Lambrecht, Germany) and low-pass filtered at 3 kHz for analysis (PatchMaster 2.53 and GraphPad Prism 5, Graphpad Software, USA). The series resistance was <10 MΩ and to minimize voltage errors was compensated to 60–80%. All measurements were performed at 24–25°C. The temperature was adjusted with a water-cooled Peltier device placed at the inlet of the chamber and controlled by a feedback device (Automatic Temperature Controller TC-324B, Warner Instruments). Cold-sensitivity was investigated with a temperature decrease to 16–18 °C.

#### Molecular modeling

The molecular model for TRPM8 was modeled using the structures of the TRPV1 ion channel in the closed state (Protein Data Bank code 3J5P) determined by electron microscopy at 3.4-Å resolution. Figures were drawn with open source Pymol (The PyMOL Molecular Graphics System, Version 1.8 Schrödinger, LLC) at http://www.pymol.org. Sequence alignment between rat TRPV1 and TRPM8 was performed with ClustalO from the European Bioinformatic Institute (EBI, http://www.ebi.ac.uk). After visual inspection, the transmembrane alignments were adjusted manually. The homology modelling was performed with the standard homology modelling protocol implemented in Yasara (version 16.12.29)^39, 40^. The visualization and edition of the molecules were also done with Yasara (http://www.yasara.org). The computational analysis of selectivity was performed with known structures of TRP channels, having the following PDB codes: TRPV1, 3j5p.pdb; TRPA1, 3j9p.pdb; TRPV2, 5hi9; and TRPV6, 5iwk.pdb. Structural alignments of TRP channels were accomplished with MUSTANG (Konagurthu AS, Whisstock JC, Stuckey PJ and Lesk AM (2006) Proteins, 64,559-574) software implemented in Yasara. The mutagenesis to determine the residues involved in ligand binding were accomplished with Yasara by doing a search through rotamer space to find the best side-chain conformation. See detailed experimental procedures within SI.

### Data Availability

All data generated or analyzed during this study are included in this published article (and its Supplementary Information files).

## Electronic supplementary material

Supporting Data
