# Supplementary material for: Synthesis, high-throughput screening and pharmacological characterization of β–lactam derivatives as TRPM8 antagonists
Source: Sci Rep. 2017 Sep 7;7:10766. doi: 10.1038/s41598-017-10913-x (PMC5589751; doi:10.1038/s41598-017-10913-x)

Synthesis, high-throughput screening and pharmacological characterization of β–lactam derivatives as TRPM8 antagonists

Robero de la Torre-Martínez, M. Angeles Bonache, Pedro J. LLabrés, Beatriz Balsera, Asia Fernández-Carvajal, Gregorio Fernández-Ballester, Antonio Ferrer-Montiel, M. Jesús Pérez de Vega, Rosario González-Muñiz*

Supplementary Information

Index

1. Chemistry. General aspects…………………………………………… 2S
2. Preparation of synthetic intermediates……. …………………………. 2S
3. Characterisation of β-lactam derivatives……………………………….. 22S
4. Tables and Figures related to compound properties, molecular modelling and biological activities ………………………………… 40S
5. NMR spectra of final β–lactam derivatives…………………………… 52S

Chemistry

1. General methods. All reagents were of commercial quality. Solvents were dried and purified by standard methods. ^{1}H NMR spectra were recorded at 300 or 400 MHz in $CDCl_3$ or DMSO-d_6. ^{13}C NMR spectra were registered at 75 or 100 MHz. Electrospray mass spectra (positive mode) were also recorded. Analytical TLC was performed on aluminium sheets with a 0.2 mm layer of silica gel F254. Silica gel 60 (230-400 mesh) was used for column chromatography. Silica gel SPE cartridges were also used for compound purification. Analytical HPLC was performed on an Eclipse Plus C_{18} (4.6 x 150 mm, 5 μM) column, with a flow rate of 1.5 mL/min, using a tuneable UV detector set at 254 nm. Mixtures of MeCN (solvent A) and 0.05% TFA in H_2O (solvent B) were used in the mobile phase. The solvent mixtures are specified in each case. All tested compounds possess a purity of ≥95%.

2. Preparation of Synthetic Intermediates

Synthesis of Ns-L-Xaa-OR^2

Triethylamine (TEA) (17.15 mmol, 2.4 mL) was added to a solution of HCl.H-L-Xaa-OR^2 (17.15 mmol, 5 g) in CH_2Cl_2 (115 mL). The mixture was stirred for 20 min. Then, TEA (22.25 mmol, 3.1 mL) and 2-nitrobenzenesulfonyl chloride (NsCl) (22.25 mmol, 4.9 g) were added to the reaction mixture at 0ºC. Stirring was continued at room temperature for 2 h. After completion, the solvent was removed and the residue was extracted with EtOAc and washed with citric acid (10%), $NaHCO_3$ (10%) and brine, successively. Finally, the organic phase was dried over Na_2SO_4, filtered, and concentrated. The residue was purified by flash chromatography on silica gel as specified in each case.

Ns-L-Phe-OMe[1] (1)

[1] Albanese, D.; Lardini, D.; Lupi, V.; Penso, M. *Eur. J. Org. Chem.* **2000**, 1443-1449.

Ns-L-Phe-OBn (2)

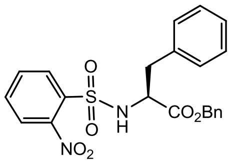

Syrup. Yield: 89%. Eluent: EtOAc:Hexane (3:1). HPLC: t_R=15.33 min (gradient from 5% to 100% of A, in 20 min). ^{1}H NMR (300 MHz, $CDCl_3$): δ 8.00-6.91 (m, 14H, Ar), 6.07 (d, 1H, *J*=9.0 Hz, NH), 4.92 (d, 1H, *J*=12.1 Hz, OCH_2), 4.87 (d, 1H, *J*=12.1 Hz, OCH_2), 4.52 (m, 1H, α-Phe), 3.13 (m, 2H, β-Phe). ^{13}C NMR (75 MMHz, $CDCl_3$): δ 171.5 (COO), 147.2, 136.6, 136.1, 135.1, 134.4, 132.8, 128.9, 128.6, 128.2, 127.7, 127.6, 127.1, 125.9, 124.2 (Ar), 66.4 (OCH_2), 58.7 (Cα), 35.9 (Cβ). MS $(ES)^+$: 441.21 $[M+H]^+$.

Ns-L-Phe-O^{t}Bu (2b)[2] (3)

Ns-L-Ala-OMe (2d)[3] (4)

Ns-L-Ala-O^{t}Bu (**2f**)[4] **(5)**

Reduction of R^4,R^5-Asp-OR^3 or R^4,R^5-Glu-OR^3 to the corresponding alcohols

Isobutyl chloroformate (^{i}BuOCOCl) (12.38 mmol, 1.60 mL) and 4-methylmorpholine (12.38 mmol, 1.36 mL) were added to a solution of R^4,R^5-Asp-OR^3 or R^4,R^5-Glu-OR^3 (12.38 mmol) in THF (12 mL) at -15ºC. The reaction mixture was stirred for 10 min. Then, the precipitated 4-methylmorpholine hydrochloride was filtered in vacuum, and washed with THF. The organic layer was treated with a solution of $NaBH_4$ (18.57 mmol, 0.7 g) in H_2O (6 mL) and the reaction was stirred for 1 h at room temperature. The solvent was removed and the residue was extracted with EtOAc and washed with

[2] Turner, J.J.; Wilschut, N.; Overkleft, H. S.; Klaffke, W.; Van der Marel, G.A.; Van Boom, J.H. *Tetrahedron Lett*. **1999**, *40*, 7039-7042.
[3] Biron, E.; Kessler, H. *J. Org. Chem*. **2005**, *70*, 5183-5189.
[4] Chapman R. N; Dimartino, G.; Arora, P. S., *J. Am. Chem. Soc*. **2004**, *126*, 12252-12253.

citric acid (10%), $NaHCO_3$ (10%) and brine, successively. Finally, the organic phase was dried over Na_2SO_4, filtered, and concentrated. The residue was purified by flash chromatography on silica gel as specified.

Boc-L-Hse-OBn (commercial) (6)

Benzyl (2*S*)-2-*tert*-Butoxycarbonylamino-5-hydroxypentanoate[5] (7)

Z-L-Hse-O^{t}Bu (commercial) (8)

Methyl (2*S*)-2- Benzyloxycarbonylamino-5-hydroxypentanoate[6] (9)

Methyl (2*S*)-2-(N-Benzyloxycarbonyl-N-methyl)amino-5-hydroxypentanoate (10)

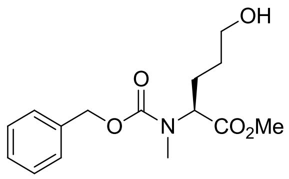

Syrup. Yield: 58%. Eluent: EtOAc:Hexane (1:1). Roamers' ratio M,m =2:1. ^{1}H NMR (300 MHz, $CDCl_3$, major rotamer): δ 7.40 (s, 5H, Ph), 5.20 (s, 2H, OCH_2), 4.90 (dd, 1H, *J*=10.5, 4.9 Hz, 2-H), 4.41 (m, 2H, 5-H), 3.75 (s, 3H, OMe), 2.92 (s, 3H, NMe), 2.08 (m, 2H, 3-H), 1.85 (m, 1H, 4-H), 1.62 (m, 1H, 4-H). MS $(ES)^+$: 318.14 $[M+Na]^+$.

Synthesis of N-alkyl-N-Ns-Xaa-OR^2 derivatives

The corresponding alcohol derivative **6-10** (3.8 mmol) and PPh_3 (3.8 mmol, 1 g) was added into a solution of the appropriate Ns-L-Xaa-OR^2 (**1-5**, 3.8 mmol) in dry THF (33 mL). The reaction mixture was treated, under Ar atmosphere, with diisopropyl azodicarboxylate (DIAD) (3.8 mmol, 0.75 mL). The reaction mixture was stirred overnight at room temperature. Then, the solvent was removed and the residue was purified by flash chromatography on silica gel, using the eluents indicated in each case.

---

[5] Jiang, S.; li, P.; Lai, C.C.; Kelley, J.A.; Roller, P.P. *J. Org. Chem*. **2006**, *71*, 7307-7314.
[6] Feichtinger, K.; Sings, H. L.; Baker, T.J.;Mathews, K.; Goodman, M. *J. Org. Chem*. **1998**, *63*, 8432-8439.

_N_-[(3_S_-Benzyloxycarbonyl-3-_tert_-butoxycarbonylamino)prop-1-yl]-Ns-L-Phe-OMe (11)

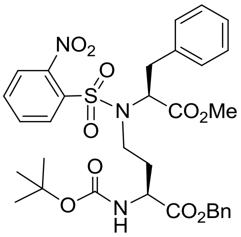

Syrup. Yield: 50% [from Ns-L-Phe-OMe (**1**) and Boc-L-Hse-OBn (**6**)]. Eluent: EtOAc:Hexane (1:2). HPLC: t_R=16.80 min (gradient of 5% to 100% of A, in 20 min). ^{1}H NMR (300 MHz, $CDCl_3$): δ 7.76-7.18 (m, 14H, Ar), 5.20 (d, 1H, *J*=12.2 Hz, OCH_2), 5.16 (d, 1H, *J*=12.2 Hz, OCH_2), 5.15 (brs, 1H, 3-NH), 4.93 (dd, 1H, *J*=8.3, 7.1 Hz, α-Phe), 4.26 (m, 1H. 3-H), 3.54 (m, 1H, 1-H), 3.53 (s, 3H, OMe), 3.40 (m, 1H, 1-H), 3.33 (dd, 1H, *J*= 14.5, 7.1 Hz, β-Phe), 2.92 (dd, 1H, *J*=14.5, 8.3 Hz, β-Phe), 2.17 (m, 1H, 2-H), 1.99 (m, 1H, 2-H), 1.45 (s, 9H, CH_3 ^{t}Bu). ^{13}C NMR (75 MHz, $CDCl_3$): 171.8 (COO), 170.9 (COO), 155.6 (OCON), 148.3, 136.0, 135.3, 133.7, 132.9, 131.7, 131.0, 129.1, 128.9, 128.8, 128.7, 128.6, 127.2, 124.2 (C, Ar), 80.3 (C ^{t}Bu), 67.6 (OCH_2), 61.5 (Cα-Phe), 52.5 (OMe), 52.00 (C3), 43.0 (C1), 36.6 (Cβ-Phe), 33.3 (C2), 28.5 (CH_3 ^{t}Bu). MS $(ES)^+$: 656.38 $[M+H]^+$.

_N_-[(3_S_-Benzyloxycarbonyl-3-_tert_-butoxycarbonylamino)prop-1-yl]-Ns-L-Phe-OBn (12)

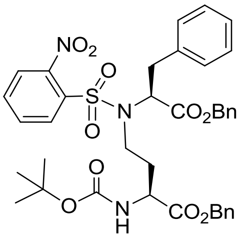

Syrup. Yield: 38% [from Ns-L-Phe-OBn (**2**) and Boc-L-Hse-OBn (**7**)]. Eluent: EtOAc:Hexane (1:3). HPLC: t_R=18.23 min (gradient of 5% to 100% of A, in 20 min). ^{1}H NMR (400 MHz, $CDCl_3$): δ 7.72-7.12 (m, 19H, Ar), 5.17 (s, 2H, OCH_2), 5.12 (d,

1H, *J*=7.7 Hz, 3-NH), 4.98 (d, 1H, *J*=13.8 Hz, OCH_2), 4.91 (d, 1H, *J*=13.8 Hz, OCH_2), 4.90 (m, 1H, α-Phe), 4.25 (m, 1H, 3-H), 3.55 (m, 1H, 1-H), 3.37 (m, 2H, β-Phe, 1-H), 2.94 (dd, 1H, *J*=14.8, 8.0 Hz, β-Phe), 2.16 (m, 1H, 2-H), 1.96 (m, 1H, 2-H), 1.45 (s, 9H, CH_3 ^{t}Bu). ^{13}C NMR (75 MHz, $CDCl_3$): 171.7 (COO), 170.1 (COO), 155.5 (OCON), 148.1, 135.9, 135.3, 134.9, 133.6, 132.7, 131.5, 130.8, 129.2, 128.9, 128,85, 128.8, 128.7, 128.6, 128.5, 128.5, 127.1, 124.1 (C, Ar), 80.2 (C ^{t}Bu), 67.52 (OCH_2), 67.5 (OCH_2), 61.5 (Cα-Phe), 52.00 (C3), 43.0 (C1), 36.8 (Cβ-Phe), 33.2 (C2), 28.4 (CH_3 ^{t}Bu). MS $(ES)^+$: 732.33 $[M+H]^+$.

***N*-[(3*S*-Benzyloxycarbonylamino-3-*tert*-butoxycarbonyl)prop-1-yl]-Ns-L-Phe-OMe (13)**

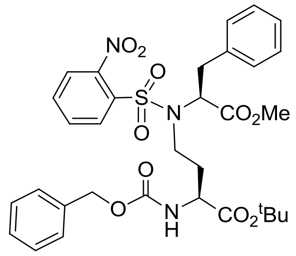

Syrup. Yield: 52% [from Ns-L-Phe-OMe (**1**) and Z-L-Hse-O^{t}Bu (**8**)]. Eluent: EtOAc:Hexane (1:2). HPLC: t_R=16.97 min (gradient of 5% to 100% of A, in 20 min). ^{1}H NMR (400 MHz, $CDCl_3$): δ 7.77 (d, 1H, *J*=7.8 Hz, Ar), 7.66-7.49 (m, 3H, Ar), 7.39-7.21 (m, 10H, Ph, Z), 5.43 (d, 1H, *J*=7.7 Hz, 3-NH), 5.17 (d, 1H, *J*=12.3 Hz, OCH_2), 5.10 (d, 1H, *J*=12.3 Hz, OCH_2), 4.93 (m, 1H, α-Phe), 4.18 (m, 1H, 3-H), 3.54 (s, 3H, OMe), 3.51 (dd, 1H, *J*= 11.8, 3.8 Hz, 1-H), 3.42 (dd, 1H, *J*= 11.8, 5.4 Hz, 1-H), 3.36 (m, 1H, β-Phe), 2.95 (dd, 1H, *J*=14.4, 8.4 Hz, β-Phe), 2.15 (m, 1H, 2-H), 1.98 (m, 1H, 2-H), 1.47 (s, 9H, CH_3 ^{t}Bu). ^{13}C NMR (75 MHz, $CDCl_3$): 170.8 (COO), 170.6 (COO), 156.0 (OCON), 148.2, 135.9, 133.7, 131.9, 130.9, 129.1, 128.7, 128.6, 128.2, 128.1, 127.1, 124.1 (C, Ar), 82.8 (C ^{t}Bu), 67.0 (OCH_2), 61.3 (Cα-Phe), 52.8 (C3), 52.5 (OMe), 42.9 (C1), 36.5 (Cβ-Phe), 33.4 (C2), 28.0 (CH_3 ^{t}Bu). MS $(ES)^+$: 678.51 $[M+Na]^+$.

***N*-[(3*S*-Benzyloxycarbonyl-3-*tert*-butoxycarbonylamino)prop-1-yl]-Ns-L-Ala-OMe (14)**

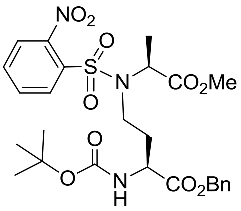

Syrup. Yield: 6% [from Ns-L-Ala-OMe (**4**) and Boc-L-Hse-OBn (**7**)]. Eluent: EtOAc:Hexane (1:2). HPLC: t_R=17.03 min (gradient of 5% to 100% of A, in 20 min). ^{1}H NMR (400 MHz, $CDCl_3$): δ 7.96 (d, 1H, *J*= 7.8 Hz, Ar-Ns), 7.86-7.56 (m, 3H, Ar-Ns), 7.36 (s, 5H, Ph), 5.21 (d, 1H, *J*= 12.3 Hz, OCH_2), 5.18 (br s, 1H, 3-NH), 5.16 (d, 1H, *J*= 12.3 Hz, OCH_2), 4.76 (m, 1H, α-Ala), 4.27 (m, 1H, 3-H), 3.57 (s, 3H, OMe), 3.51 (m, 1H, 1-H), 3.22 (m, 1H, 1-H), 2.23 (m, 1H, 2-H), 2.09 (m, 1H, 2-H), 1.47 (d, 3H, *J*= 7.4 Hz, β-Ala), 1.43 (s, 9H, CH_3 ^{t}Bu). ^{13}C NMR (100 MHz, $CDCl_3$): 171.8 (COO), 165.2 (COO), 155.5 (OCON), 148.1, 135.3, 133.7, 131.7, 131.1, 128.8, 128.7, 128.6, 128.5, 124.2 (Ar), 80.3 (C ^{t}Bu), 67.6 (OCH_2), 56.2 (Cα-Ala), 52.5 (OMe), 51.9 (C3), 42.7 (C1), 33.7 (C2), 28.4 (CH_3 ^{t}Bu), 16.8 (Cβ-Ala). MS (ES)$^+$: 602.41 [M+Na]$^+$.

***N*-[(3*S*-Benzyloxycarbonylamino-3-*tert*-butoxycarbonyl)prop-1-yl]-Ns-L-Ala-OMe (15)**

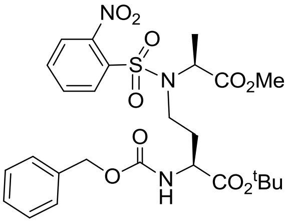

Syrup. Yield: 58% [from Ns-L-Ala-OMe (**4**) and Z-L-Hse-O^{t}Bu (**8**)]. Eluent: EtOAc:Hexane (1:2). HPLC: t_R=15.49 min (gradient of 5% to 100% of A, in 20 min). ^{1}H NMR (400 MHz, $CDCl_3$): δ 8.00 (dd, 1H, *J*= 7.9, 1.4 Hz, Ar-Ns), 7.67 (dd, 1H, *J*= 7.6, 1.4 Hz, Ar-Ns), 7.63-7.58 (m, 2H, Ar-Ns), 7.37-7.31 (m, 5H, Ph, Z), 5.40 (d, 1H,

J=7.1 Hz, NH, Z), 5.15 (d, 1H, J=12.0 Hz, OCH_2), 5.09 (d, 1H, J=12.0 Hz, OCH_2), 4.76 (q, 1H, J=7.4 Hz, α-Ala), 4.19 (m, 1H, 3-H), 3.58 (s, 3H, OMe), 3.48 (m, 1H, 1-H), 3.23 (m, 1H, 1-H), 2.14 (m, 2H, 2-H), 1.47 (m, 12H, CH_3 ^{t}Bu, β-Ala). ^{13}C NMR (75 MHz, $CDCl_3$): 171.7 (COO), 170.6 (COO), 156.0 (OCON), 148.0, 136.4, 133.7, 131.7, 131.0, 128.6, 128.3, 128.1, 124.2 (C, Ar), 82.8 (C ^{t}Bu), 67.0 (OCH_2), 56.1 (Cα-Ala), 52.8 (C3), 52.5 (OMe), 42.7 (C1), 33.8 (C2), 28.0 (CH_3 ^{t}Bu), 16.8 (Cβ-Ala). MS $(ES)^+$: 602.48 $[M+Na]^+$.

***N*-[(4*S*-Benzyloxycarbonyl-4-*tert*-butoxycarbonylamino)but-1-yl]-Ns-L-Phe-OBn (16)**

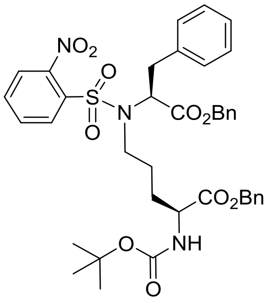

Syrup. Yield: 62% [from Ns-L-Phe-OBn (**1**) and Boc-L-Hse-OBn (**7**)]. Eluent: EtOAc:Hexane (1:2). t_R=18.60 min (gradient of 5% to 100% of A, in 20 min). ^{1}H NMR (300 MHz, $CDCl_3$): δ 7.87-6.84 (m, 19H, Ar), 5.11 (d, 1H, J=12.0 Hz, OCH_2), 5.06 (d, 1H, J=12.0 Hz, OCH_2), 4.93 (m, 1H, NH), 4.90 (d, 1H, J=12.1 Hz , OCH_2), 4.85 (d, 1H, J=12.2 Hz, OCH_2), 4.81 (m, 1H, α-Phe), 4.21 (m, 1H, 4-H), 3.40 (ddd, 1H, J=15.3, 9.9, 5.2 Hz, 1-H), 3.27 (dd, 1H, J=14.1, 8.0 Hz, β-Phe), 3.17 (m, 1H, 1-H), 2.92 (dd, 1H, J=14.0, 7.1 Hz, β-Phe), 1.60 (m, 4H, 3-H, 2-H), 1.44 (s, 9H, CH_3 ^{t}Bu). ^{13}C NMR ($CDCl_3$): δ 172.4, 170.1 (COO), 155.5 (OCON), 148.2, 136.3, 135.4, 134.9, 133.5, 133.0, 131.5, 131.0, 129.3, 128.8, 128.7, 128.6, 128.5, 128.4, 127.1, 124.1 (Ar), 80.1 (C, ^{t}Bu), 67.5, 67.3 (OCH_2), 61.5 (Cα-Phe), 53.1 (C4), 46.1 (C1), 36.9 (Cβ-Phe), 30.1 (C3), 28.4 (CH_3 ^{t}Bu), 26.4 (C2). MS $(ES)^+$: 746.29 $[M+H]^+$.

***N*-[(4*S*-Benzyloxycarbonylamino-4-methyloxycarbonyl)-but-1-yl]-Ns-L-Phe-O^{t}Bu (17)**

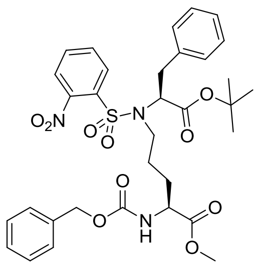

Syrup. Yield: 68% [from Ns-L-Phe-O^{t}Bu (**3**) and **9**]. HPLC-MS: t_R=8.53 min (gradient of 30% to 95% of A, in 10 min). ^{1}H NMR (400 MHz, $CDCl_3$): δ 7.84 (d, 1H, *J*=7.8 Hz, Ar-Ns), 7.63 (m, 1H, Ar-Ns), 7.56 (m, 2H, Ar-Ns), 7.36-7.20 (m, 10H, Ar), 5.33 (d, 1H, *J*=8.2 Hz, 4-NH), 5.11 (s, 2H, OCH_2), 4.75 (t, 1H, *J*=7.6 Hz, α-Phe), 4.33 (m, 1H, 4-H), 3.74 (s, 3H, OMe), 3.55-3.48 (m, 1H, 1-H), 3.35-3.27 (m, 1H, 1-H, β-Phe), 3.99 (dd, 1H, *J*= 14.5, 7.5 Hz, β-Phe), 1.87-1.80 (m, 1H, 3-H), 1.79-1.58 (m, 3H, 3-H, 2-H), 1.23 (s, 9H, CH_3 ^{t}Bu). MS (ES)$^+$: 670.48 [M+H]$^+$.

***N*-[4*S*-[(N-Benzyloxycarbonyl-N-methyl)amino-4-metoxycarbonyl]but-1-yl]-Ns-L-Phe-O^{t}Bu (18)**

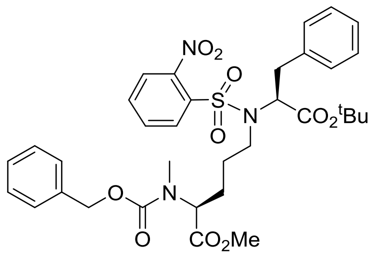

Syrup. Yield: 91% [from Ns-L-Phe-O^{t}Bu (**2**) and **10**]. Eluent: EtOAc:Hexane (1:1). HPLC: t_R=17.61 min (gradient of 5% to 100% of A, in 20 min). Roamers' ratio M,m =2:1. ^{1}H NMR (400 MHz, $CDCl_3$, major rotamer): δ 7.87 (dd, 1H, *J* = 7.8, 1.2 Ar-Ns), 7.60 (m, 1H, Ar-Ns), 7.53 (m, 2H, Ar-Ns), 7.26 (m, 10H, Ph, Z), 5.16 (d, 1H, *J*=12.5 Hz, OCH_2), 5.12 (d, 1H, *J*=12.5 Hz, OCH_2), 4.79 (t, 1H, *J*=7.8 Hz, α-Phe), 4.74 (dd, 1H, *J* = 10.5, 4.8 Hz, 4-H), 3.69 (s, 3H, OMe), 3.55 (m, 1H, 1-H), 3.31(dd, 1H, *J*=14.3,

7.9 Hz, β-Phe), 3.24 (m, 1H, 1-H), 2.97 (dd, 1H, *J*=14.3, 7.9 Hz, β-Phe), 2.83 (s, 3H, NMe), 1.92 (m, 1H, 3-H), 1.71 (m, 3H, 3-H, 2-H), 1.21 (s, 9H, CH_3 *ᵗ*Bu). ^{13}C NMR (75 MHz, $CDCl_3$): 171.8 (COO), 169.2 (COO), 157.0 (OCON), 148.3, 136.7, 133.5, 131.7, 129.2, 128.7, 128.6, 127.9, 126.9, 123.9 (C, Ar), 82.4 (C ^{t}Bu), 67.6 (OCH_2), 61.8 (Cα-Phe), 57.9 (C4), 52.3 (OMe), 45.8 (C1), 36.9 (Cβ-Phe), 30.6 (NMe), 27.7 (CH_3 ^{t}Bu), 27.1 (C3), 26.1 (C2). MS $(ES)^+$: 706.68 $[M+Na]^+$.

***N*-[(4*S*-Benzyloxycarbonylamino-4-methyloxycarbonyl)-but-1-yl]-Ns-L-Ala-O^{t}Bu (19)**

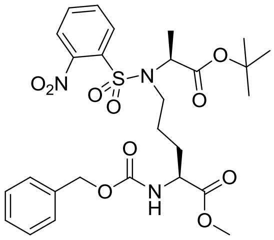

Syrup. Yield: 63% (from Ns-L-Ala-O^{t}Bu (**5**) and **9**). HPLC-MS: t_R=7.62 min (gradient of 30% to 95% of A, in 10 min). ^{1}H NMR (400 MHz, $CDCl_3$): δ 8.05 (d, 1H, *J*=6.5 Hz, Ar- Ns), 7.64 (m, 2H, Ar- Ns), 7.57 (m, 1H, Ar- Ns), 7.36-7.30 (m, 5H, Ar), 5.34 (d, 1H, *J*=7.9 Hz, 4-NH), 5.11 (s, 2H, OCH_2), 4.62 (q, 1H, *J*=7.2 Hz, α-Ala), 4.35 (q, 1H, J = 7.2 Hz, 4-H), 3.74 (s, 3H, OMe), 3.52-3.44 (m, 1H, 1-H), 3.16-3.09 (m, 1H, 1-H), 1.86-1.64 (m, 4H, 3-H, 2-H), 1.45 (d, 3H, *J*=7.3 Hz, β-Ala), 1.32 (s, 9H, CH_3 ^{t}Bu). MS $(ES)^+$: 594.32 $[M+H]^+$.

Removal of the Ns group

K_2CO_3 (3.3 mmol, 0.455 g) was added to a solution of the corresponding N-alkyl-N-Ns-Xaa derivatives **11-19** (1.1 mmol) in CH_3CN (20 mL). Thiophenol (2.2 mmol, 0.22 mL) was added to the mixture and the reaction was stirred overnight. The solvent was removed and the residue was extracted with EtOAc and washed with H_2O and brine,

successively. Finally, the organic phase was dried over dry Na_2SO_4, filtered, and concentrated. The residue was purified by flash chromatography on silica gel, using the eluent mixture indicated in each case.

***N*-[(3*S*-Benzyloxycarbonyl-3-*tert*-butoxycarbonylamino)prop-1-yl]-L-Phe-OMe (20)**

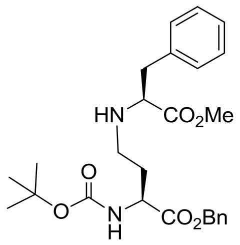

Syrup. Yield: 85% (from **11**). Eluent: EtOAc:Hexane (1:1). HPLC: t_R=11.98 min (gradient of 5% to 100% of A, in 20 min). ^{1}H NMR (300 MHz, $CDCl_3$): δ 7.38-7.16 (m, 10H, Ar), 5.81 (d, 1H, *J*=7.6 Hz, 3-NH), 5.22 (d, 1H, *J*=12.5 Hz, OCH_2), 5.14 (d, 1H, *J*=12.5 Hz, OCH_2), 4.41(m, 1H, 3-H), 3.66 (s, 3H, OMe), 3.50 (t, 1H, *J*=6.7 Hz, α-Phe), 2.94 (m, 2H, β-Phe), 2.75 (m, 1H, 1-H), 2.51 (m, 1H, 1-H), 1.96 (m, 1H, 2-H), 1.82 (m, 1H, 2-H), 1.48 (s, 9H, CH_3 ^{t}Bu).^{13}C NMR (75 MHz, $CDCl_3$): 172.9 (COO), 170.7 (COO), 155.9 (OCON), 136.1, 128.7, 128.5, 127.4, 127.1, 126.9 (C, Ar), 79.9 (C ^{t}Bu), 65.1 (OCH_2), 55.2 (Cα-Phe), 52.5 (OMe), 52.0 (C3), 41.5 (C1), 35.1 (Cβ-Phe), 28.6 (C2), 28.3 (CH_3 ^{t}Bu). MS $(ES)^+$: 471.02 $[M+H]^+$.

***N*-[(3*S*-Benzyloxycarbonyl-3-*tert*-butoxycarbonylamino)prop-1-yl]-L-Phe-OBn (21)**

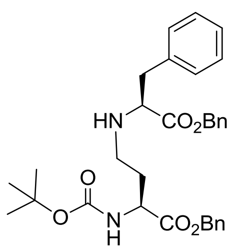

Syrup. Yield: 90% (from **12**). Eluent: EtOAc:Hexane (1:1). HPLC: t_R=15.47 min (gradient of 5% to 100% of A, in 20 min). ^{1}H NMR (400 MHz, $CDCl_3$): δ 7.33-7.07 (m, 15H, Ar), 5.75 (d, 1H, *J*=7.7 Hz, 3-NH), 5.17 (d, 1H, *J*=12.4 Hz, OCH_2), 5.10 (d, 1H,

J=12.4 Hz, OCH_2), 5.04 (s, 2H, OCH_2), 4.37 (m, 1H, 3-H), 3.50 (m, 1H, α-Phe), 2.94 (dd, 1H, *J*=13.5, 6.6 Hz , β-Phe), 2.86 (dd, 1H, *J*=13.5, 7.3 Hz, β-Phe), 2.72 (m, 1H, 1-H), 2.47 (m, 1H, 1-H), 1.92 (m, 1H, 2-H), 1.79 (m, 1H, 2-H), 1.44 (s, 9H, CH_3 ^{t}Bu). ^{13}C NMR (75 MHz, $CDCl_3$): 174.2 (COO), 172.6 (COO), 155.6 (OCON), 137.0, 135.62, 135.6, 129.3, 128.7, 128.6, 128.55, 128.5, 128.4, 126.8 (C, Ar), 79.8 (C, ^{t}Bu), 67.0, 66.6 (OCH_2), 62.7 (Cα-Phe), 52.5 (C3), 44.2 (C1), 39.8 (Cβ-Phe), 31.7 (C2), 28.5 (CH_3 ^{t}Bu). MS (ES)$^+$: 547.92 $[M+H]^+$.

***N*-[(3*S*-Benzyloxycarbonylamino-3-*tert*-butoxycarbonyl)prop-1-yl]-L-Phe-OMe (22)**

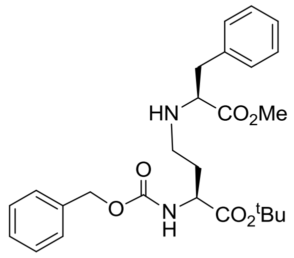

Syrup. Yield: 78% (from **13**). Eluent: EtOAc:Hexane (1:1). HPLC: t_R=10.73 min (gradient of 5% to 100% of A, in 20 min). ^{1}H NMR (400 MHz, $CDCl_3$): δ 7.36-7.14 (m, 10H, Ph, Z), 5.95 (d, 1H, *J*=7.5 Hz, 3-NH), 5.10 (s, 2H, OCH_2), 4.27 (m, 1H, 3-H), 3.63 (s, 3H, OMe), 3.51 (t, 1H, *J*=6.5 Hz, α-Phe), 2.95 (m, 2H, β-Phe), 2.73 (m, 1H, 1-H), 2.53 (m, 1H, 1-H), 1.97 (m, 1H, 2-H), 1.77 (m, 2H, 2-H), 1.44 (s, 9H, CH_3 ^{t}Bu). ^{13}C NMR (75 MHz, $CDCl_3$): 174.5 (COO), 171.3 (COO), 156.0 (OCON), 136.9, 136.4, 129.0, 128.3, 127.9, 126.6 (Ar), 81.7 (C ^{t}Bu), 66.6 (OCH_2), 62.6 (Cα-Phe), 53.2 (C3), 51.5 (OMe), 44.1 (C1), 39.5 (Cβ-Phe), 31.7 (C2), 27.8 (CH_3 ^{t}Bu). MS (ES)$^+$: 471.37 $[M+H]^+$.

***N*-[(3*S*-Benzyloxycarbonyl-3-*tert*-butoxycarbonylamino)prop-1-yl]-L-Ala-OMe (23)**

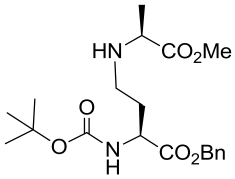

Syrup. Yield: 43% (from **14**). Eluent: EtOAc:Hexane (2:1). HPLC: t_R=12.15 min (gradient of 5% to 100% of A, in 20 min). ^{1}H NMR (400 MHz, $CDCl_3$): δ 7.34 (s, 5H, Ph), 5.76 (d, 1H, *J*=6.0 Hz, 3-NH), 5.18 (d, 1H, *J*=16.0 Hz, OCH_2), 5.11 (d, 1H, *J*=16.0 Hz, OCH_2), 4.39 (m, 1H, 3-H), 3.69 (s, 3H, OMe), 3.28 (q, 1H, *J*=7.0 Hz, α-Ala), 2.71 (m, 1H, 1-H), 2.49 (m, 1H, 1-H), 1.87 (m, 2H, 2-H), 1.43 (s, 9H, $CH_{3,}$ ^{t}Bu,), 1.24 (d, 1H, *J*=7.0 Hz, β-Ala). ^{13}C NMR (100 MHz, $CDCl_3$): 175.9 (COO), 172.6 (COO), 155.69 (OCON), 135.6, 128.7. 128.4, 128.3, 127.1 (C, Ar), 79.9 (C ^{t}Bu), 67.0 (OCH_2), 56.7 (Cα-Ala), 52.5 (C3), 51.9 (OMe), 43.9 (C1), 31.9 (C2), 28.4 (CH_3 ^{t}Bu), 19.1 (Cβ-Ala). MS $(ES)^+$: 395.35 $[M+H]^+$.

***N*-[(3*S*-Benzyloxycarbonylamino-3-*tert*-butoxycarbonyl)prop-1-yl]-L-Ala-OMe (24)**

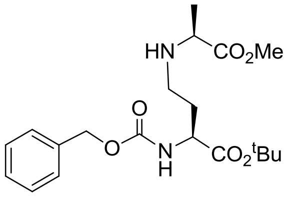

Syrup. Yield: 78% (from **15**). Eluent: EtOAc:Hexane (2:1). HPLC: t_R=9.14 min (gradient of 5% to 100% of A, in 20 min). ^{1}H NMR (400 MHz, $CDCl_3$): δ 7.33 (m, 5H, Z), 5.97 (d, 1H, *J*=7.6 Hz, 3-NH), 5.12 (d, 1H, *J*=12.0 Hz, OCH_2), 5.06 (d, 1H, *J*=12.0 Hz, OCH_2), 4.30 (m, 1H, 3-H), 3.69 (s, 3H, OMe), 3.32 (q, 1H, *J*=7.0 Hz, α-Ala), 2.73 (m, 1H, 1-H), 2.53 (m, 1H, 1-H), 1.97 (m, 1H, 2-H), 1.79 (m, 1H, 2-H), 1.44 (s, 9H, CH_3 ^{t}Bu), 1.26 (d, 3H, *J*=7.0 Hz, β-Ala). ^{13}C NMR (75 MHz, $CDCl_3$): 175.9 (COO),

171.5 (COO), 156.2 (OCON), 136.6, 128.6, 128.15, 128.1 (Ar), 82.0 (C *t*Bu), 66.8 (OCH_2), 56.4 (Cα-Ala), 53.4 (C3), 51.9 (OMe), 44.0 (C1), 32.1 (C2), 28.0 (CH_3 *t*Bu), 19.0 (Cβ-Ala). MS $(ES)^+$: 395.42 $[M+H]^+$.

***N*-[(4*S*-Benzyloxycarbonyl-4-*tert*-butoxycarbonylamino)but-1-yl]-H-L-Phe-OBn (25)**

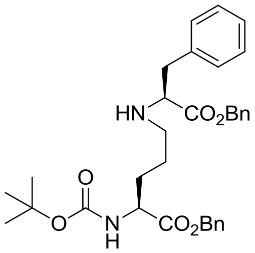

Syrup. Yield: 81% (from **16**). Eluent: EtOAc:Hexane (1:2). HPLC: t_R=17.63 min (gradient of 5% to 100% of A, in 20 min). ^{1}H NMR (300 MHz, $CDCl_3$): δ 7.74-6.87 (m, 15H, Ar), 5.62 (d, 1H, *J*=8.4 Hz, 4-NH), 5.22 (d, 1H, *J*=12.4 Hz, OCH_2), 5.16 (d, 1H, *J*=12.4 Hz, OCH_2), 5.09 (s, 2H, OCH_2), 4.34 (m, 1H, 4-H), 3.55 (t, 1H, *J*=7.5 Hz, α-Phe), 3.00 (m, 2H, β-Phe), 2.64 (m, 1H, 1-H), 2.44 (m, 1H, 1-H), 1.77 (m, 2H, 3-H), 1.46 (m, 9H, *t*Bu). ^{13}C NMR (75 MHz, $CDCl_3$): δ 174.5, 172.7 (COO), 155.6 (OCON), 137.1, 135.6, 129.4, 128.9, 128.7, 128.6, 128.55, 128.5, 128.4, 128.35, 126.8 (Ar), 79.9 (C *t*Bu), 67.0, 66.6 (OCH_2), 62.9 (Cα-Phe), 53.5 (C4), 47.5 (C1), 39.8 (Cβ-Phe), 30.4 (C2), 28.5 (CH_3 *t*Bu), 25.8 (C3). MS $(ES)^+$: 561 $[M+H]^+$.

***N*-[(4*S*-Benzyloxycarbonylamino-4-metoxycarbonyl)but-1-yl]-L-Phe-O^{t}Bu (26)**

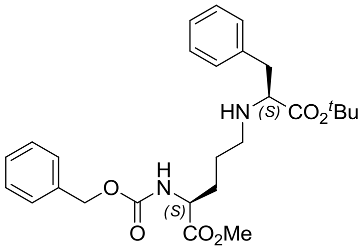

Syrup. Yield: 89% (from **17**). HPLC: t_R= 5.29 min (gradient of 15% to 95% of A, in 10 min). HPLC-MS: t_R=6.77 (gradient of 15% to 95% of A, in 10 min). ^{1}H NMR (300

MHz, $CDCl_3$): δ 7.26-7.08 (m, 10H, Ar), 6.03 (d, 1H, *J*=7.8 Hz, 4-NH), 5.03 (s, 2H, OCH_2), 4.05 (m, 1H, 4-H), 3.66 (s, 3H, OMe), 3.25 (dd, 1H, *J*=7.7, 6.5 Hz, α-Phe), 2.87 (dd, 1H, *J*=13.4, 6.4 Hz, β-Phe), 2.75 (dd, 1H, *J*=13.4, 7.7 Hz, β-Phe), 2.56 (m, 1H, 1-H), 2.35 (m, 1H, 1-H), 1.71 (m, 1H, 3-H), 1.48-1.38 (m, 3H, 3-H, 2-H), 1.25 (s, 9H, CH_3 ^{t}Bu). MS $(ES)^+$: 485.32 $[M+H]^+$.

_N_-[4_S_-[(N-Benzyloxycarbonyl-N-methyl)amino-4-metoxycarbonyl]but-1-yl]-L-Phe-O^{t}Bu (27)

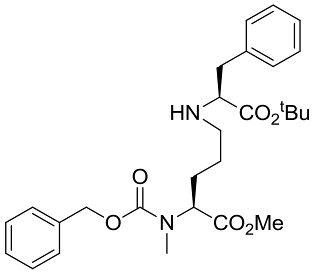

Syrup. Yield: 88% (from **18**). Eluent: EtOAc:Hexane (1:1). HPLC: t_R=10.85 min (gradient of 5% to 100% of A, in 20 min). Roamers' ratio M,m =2:1. ^{1}H NMR (400 MHz, $CDCl_3$, major rotamer): δ 7.37-7.19 (m, 10H, Ph, Z), 5.15 (s, 2H, OCH_2), 4.78 (dd, 1H, *J*=10.8, 5.1 Hz, 3-H), 3.70 (s, 3H, OMe), 3.41(t, 1H, *J*=7.0 Hz, α-Phe), 2.93 (m, 2H, β-Phe), 2.83 (s, 3H, NMe), 2.63 (m, 2H, 1-H), 1.94 (m, 2H, 3-H), 1.73 (m, 2H, 2-H), 1.33 (s, 9H, CH_3 ^{t}Bu). ^{13}C NMR (75 MHz, $CDCl_3$): 173.8 (COO), 172.1 (COO), 157.1 (OCON), 137.5, 129.5, 128.6, 128.3, 128.1, 127.9, 127.8, 126.6 (C, Ar), 81.3 (C ^{t}Bu), 67.6 (OCH_2), 63.3 (Cα-Phe), 58.4 (C4), 52.2 (OMe), 47.4 (C1), 36.8 (Cβ-Phe), 30.4 (NMe), 28.1 (CH_3 ^{t}Bu), 26.6 (C2), 26.4 (C3). MS $(ES)^+$: 499.44 $[M+H]^+$.

_N_-[(4_S_-Benzyloxycarbonylamino-4-metoxycarbonyl)but-1-yl]-L-Ala-O^{t}Bu (28)

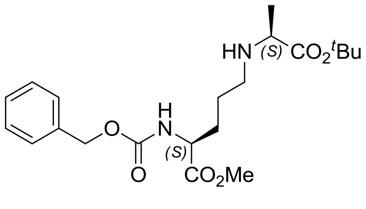

Syrup. Yield: 77% (from **19**). HPLC-MS: t_R= 5.20 min (gradient of 15% to 95% of A, in 10 min). ^{1}H NMR (300 MHz, $CDCl_3$): δ 7.26 (s, 5H, Ph), 6.15 (d, 1H, *J*=7.7 Hz, 4-NH), 5.01 (s, 2H, OCH_2), 4.28 (m, 1H, 4-H), 3.65 (s, 3H, OMe), 3.06 (q, 1H, *J*=6.9 Hz, α-Ala), 2.58 (m, 1H, 1-H), 2.33 (dd, 1H, 1-H), 1.76 (m, 2H, 3-H), 1.45 (m, 2H, 2-H), 1.37 (s, 9H, CH_3 ^{t}Bu), 1.14 (d, 3H, *J* = 6.9 Hz, β-Ala). MS $(ES)^+$: 409.33 $[M+H]^+$.

Synthesis of N-alkyl-N-2-chloroalkanoyl Xaa derivatives

Propylene oxyde (34.3 mmol, 2.4 mL) was added to a solution of **20-28** (2.28 mmol) in dry THF (9 mL). Chloroacetyl chloride (2.75 mmol, 0.22 mL) or (R,*S*)-2-chloropropionyl chloride (2.75 mmol, 0.27 mL) was added to the reaction mixture at 0ºC, and stirred overnight t room temperature. The solvent was removed and the residue was purified by flash chromatography on silica gel, using the eluent mixture indicated in each case.

N-Chloroacetyl-*N*-[(3*S*-benzyloxycarbonyl-3-*tert*-butoxycarbonylamino)prop-1-yl]-L-Phe-OMe (29)

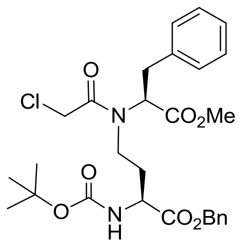

Syrup. Yield: 92% (from **20**). Eluent: EtOAc:Hexane (1:1). HPLC: t_R=15.70 min (gradient of 5% to 100% of A, in 20 min). Roamers' ratio M,m =9:1. ^{1}H NMR (400 MHz, $CDCl_3$, major rotamer): δ 7.35-7.09 (m, 10H, Ph), 5.18 (m, 1H, 3-NH), 5.16 (d, 1H, *J*=12.0 Hz, OCH_2), 5.07 (d, 1H, *J*=12.0 Hz, OCH_2), 4.16 (m, 1H, 3-H), 3.92 (m, 1H, α-Phe), 3.91 (d, 1H, *J*=12.5 Hz, CH_2Cl), 3.86 (d, 1H, *J*=12.5 Hz, CH_2Cl), 3.69 (s, 3H, OMe), 3.30 (m, 2H, β-Phe), 3.13 (m, 1H, 1-H), 2.70 (m, 1H, 1-H), 1.90 (m, 1H, 2-H), 1.52 (m, 1H, 2-H), 1.41 (s, 9H, CH_3 ^{t}Bu). ^{13}C NMR (75 MHz, $CDCl_3$, major

rotamer): 171.6 (COO), 170.4 (COO), 166.7 (CON), 155.4 (OCON), 137.8, 135.1, 129.4, 128.9, 128.75, 128.7, 127.0 (C, Ar), 80.5 (C ^{t}Bu), 67.6 (OCH_2), 63.3 (Cα-Phe), 52.6 (OMe), 51.8 (C3), 47.0 (CH_2Cl), 41.0 (C1), 34.4 (Cβ-Phe), 31.6 (C2), 28.4 (CH_3 ^{t}Bu). MS (ES)$^+$: MS (ES)$^+$: 569.30 [M+Na]$^+$.

N-Chloroacetyl-*N*-[(3*S*-benzyloxycarbonyl-*3-tert*-butoxycarbonylamino)prop-1-yl]-L-Phe-OBn (30)

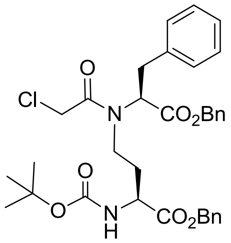

Syrup. Yield: 99% (from **21**). Eluent: EtOAc:Hexane (1:2). HPLC: t_R=17.80 min (gradient of 5% to 100% of A, in 20 min). Roamers' ratio M,m =5:2. ^{1}H NMR (400 MHz, $CDCl_3$, major rotamer): δ 7.35-7.08 (m, 15H, Ar), 5.30 (d, 1H, *J*=8.0 Hz, 3-NH), 5.15 (m, 2H, OCH_2), 5.13 (d, 1H, *J*=12.1 Hz, OCH_2), 5.04 (d, 1H, *J*=12.1 Hz, OCH_2), 4.11 (m, 1H, α-Phe), 3.95 (m, 1H, 3-H), 3.88 (d, 1H, *J*=12.4 Hz, CH_2Cl), 3.83 (d, 1H, *J*=12.4 Hz, CH_2Cl), 3.31 (m, 2H, 1-H), 3.10 (dd, 1H, *J*=15.6, 5.3 Hz, β-Phe), 2.73 (m, 1H,. β-Phe), 1.86 (m, 2H, 2-H), 1.40 (s, 9H, CH_3 ^{t}Bu). ^{13}C NMR (75 MHz, $CDCl_3$): 171.5 (COO), 169.7 (COO), 166.6 (CON), 155.4 (OCON), 137.7, 135.6, 135.0, 129.4, 128.9, 128.7, 128.6, 128.4, 127.0 (C Ar), 80.5 (C ^{t}Bu), 67.6 (OCH_2), 67.4 (OCH_2), 63.5 (Cα-Phe), 51.7 (C3), 47.0 (CH_2Cl), 41.0 (C1), 34.4 (Cβ-Phe), 31.6 (C2), 28.4 (CH_3 ^{t}Bu). MS (ES)$^+$: 624.42 [M+H]$^+$

N-Chloroacetyl-*N*-[(3*S*-benzyloxycarbonylamino-3-*tert*-butoxycarbonyl)prop-1-yl]-L-Phe-OMe (31)

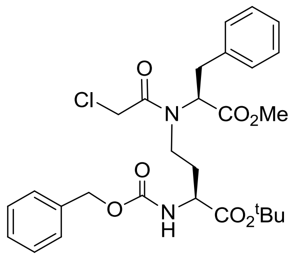

Syrup. Yield: 90% (from **22**). Eluent: EtOAc:Hexane (1:1). HPLC: t_R=15.66 min (gradient of 5% to 100% of A, in 20 min). Roamers' ratio M,m =3:1. ^{1}H NMR (400 MHz, $CDCl_3$, major rotamer): δ 7.37-7.13 (m, 10H, Ph), 5.46 (d, 1H, *J*=7.2 Hz, 3-NH), 5.13 (d, 1H, *J*=12.0 Hz, OCH_2), 5.04 (d, 1H, *J*=12.0 Hz, OCH_2), 4.09 (m, 1H, 3-H), 3.99 (m, 1H, α-Phe), 3.99 (d, 1H, *J*=12.0 Hz, CH_2Cl), 3.91 (d, 1H, *J*=12.0 Hz, CH_2Cl), 3.73 (s, 3H, OMe), 3.30 (m, 2H, β-Phe), 3.20 (m, 1H, 1-H), 2.74 (m, 1H, 1-H), 1.88 (m, 1H, 2-H), 1.59 (m, 1H, 2-H), 1.42 (s, 9H, CH_3 ^{t}Bu). ^{13}C NMR (75 MHz, $CDCl_3$): 171.3, 170.3 (COO), 167.7 (CON), 156.0 (OCON), 137.6, 129.3, 128.7, 128.6, 128.4, 128.2, 126.9 (Ar), 83.0 (C ^{t}Bu), 67.2 (OCH_2), 63.2 (Cα-Phe), 52.9 (OMe), 52.6 (C3), 47.0 (CH_2Cl), 40.9 (C1), 34.3 (Cβ-Phe), 31.5 (C2), 28.3 (CH_3 ^{t}Bu). MS $(ES)^+$: 569.51 $[M+Na]^+$.

N-Chloroacetyl-*N*-[(3*S*-benzyloxycarbonyl-3-*tert*-butoxycarbonylamino)prop-1-yl]-L-Ala-OMe (32)

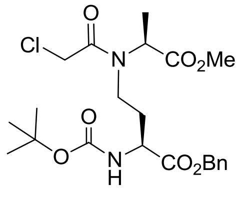

Syrup. Yield: 80% (from **23**). Eluent: EtOAc:Hexane (1:2). HPLC: t_R=13.15 min (gradient of 5% to 100% of A, in 20 min). Roamers' ratio M,m =5:3. ^{1}H NMR (400 MHz, $CDCl_3$, major rotamer): δ 7.36 (s, 5H, Ph), 5.47 (d, 1H, *J*=7.6 Hz, 3-NH), 5.26 (d, 1H, *J*=12.0 Hz, OCH_2), 5.14 (d, 1H, *J*=12.0 Hz, OCH_2), 4.35 (m, 1H, 3-H), 4.16 (q, 1H, J = 7.0 Hz, α-Ala), 4.09 (s, 2H, CH_2Cl), 3.68 (s, 3H, OMe), 3.55 (m, 1H, 1-H), 3.39 (t,

1H, *J*=8.0 Hz, 1-H), 2.00 (m, 2H, 2-H), 1.44 (d, 3H, *J*=7.0 Hz, β-Ala), 1.43 (s, 9H, CH_3, ^{t}Bu). ^{13}C NMR (75 MHz, $CDCl_3$): 171.55, 171.5 (COO), 166.5 (CON), 155.6 (OCON), 135.0, 129.0. 128.9, 128.8 (C, Ar), 80.8 (C ^{t}Bu), 67.8 (OCH_2), 55.5 (Cα-Ala), 53.0 (C3), 52.5 (OMe), 44.7 (CH_2Cl), 41.1 (C1), 30.7 (C2), 28.4 (CH_3 ^{t}Bu), 14.5 (Cβ-Ala). MS $(ES)^+$: 493.35 $[M+Na]^+$.

N-Chloroacetyl-*N*-[(3*S*-benzyloxycarbonylamino-3-*tert*-butoxycarbonyl)prop-1-yl]-L-Ala-OMe (33)

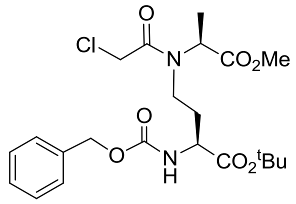

Syrup. Yield: 92% (from **24**). Eluent: EtOAc:Hexane (1:1). HPLC: t_R=13.54 min (gradient of 5% to 100% of A, in 20 min). Roamers' ratio M,m =2:1. ^{1}H NMR (400 MHz, $CDCl_3$, major rotamer): δ 7.35 (m, 5H, Ph), 5.58 (d, 1H, *J*=7.0 Hz, 3-NH), 5.14 (d, 1H, *J*=12.0 Hz, OCH_2), 5.08 (d, 1H, *J*=12.0 Hz, OCH_2), 4.24 (m, 2H, 3-H, α-Ala), 4.12 (d, 1H, *J*=12.5 Hz, CH_2Cl), 4.02 (d, 1H, *J*=12.5 Hz, CH_2Cl), 3.70 (s, 3H, OMe), 3.42 (m, 2H, 1-H), 2.22 (m, 1H, 2-H), 1.99 (m, 1H, 2-H), 1.48 (s, 9H, CH_3 ^{t}Bu), 1.47 (d, 3H, *J*=6.4 Hz, β-Ala). ^{13}C NMR (75 MHz, $CDCl_3$): 171.4 (COO), 170.4 (COO), 166.6 (CON), 156.0 (OCON), 136.1, 133.2, 130.0, 128.6, 128.5, 128.3, 128.2, 128.0 (Ar), 83.1 (C ^{t}Bu), 67.1 (OCH_2), 55.4 (Cα-Ala), 52.8 (C3), 52.4 (OMe), 44.6 (CH_2Cl), 41.0 (C1), 32.5 (C2), 27.9 (CH_3 ^{t}Bu), 14.4 (Cβ-Ala). MS $(ES)^+$: 493.42 $[M+Na]^+$.

***N*-[(3*S*-Benzyloxycarbonyl-3-*tert*-butoxycarbonylamino)prop-1-yl]-N-(2'R,*S*-chloropropanoyl)-L-Phe-OBn (34)**

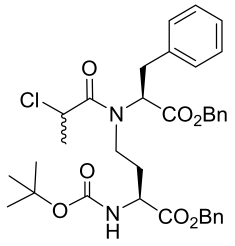

Syrup. Yield: 68% (from **21**). Eluent: EtOAc:Hexane (1:3). HPLC: t_R=10.64 min (gradient of 5% to 100% of A, in 20 min). Diastereoisomers ratio M,m =5:4. ^{1}H NMR (400 MHz, $CDCl_3$, major isomer, rotamers ratio 9:1, major rotamer): δ 7.35-7.04 (m, 15H, Ar), 5.14 (d, 1H, J=12.4 Hz, OCH_2), 5.13 (s, 2H, OCH_2), 5.03 (d, 1H, *J*=12.1 Hz, OCH_2), 4.93 (d, 1H, *J*=6.5 Hz, 3-NH), 4.31 (q, 2H, *J*=6.5 Hz, 1'-H), 4.06 (m, 1H, 3-H), 3.95 (m, 1H, α-Phe), 3.33 (dd, 1H, *J*=14.1, 5.3, β-Phe), 3.26 (m, 1H, β-Phe), 3.00 (m, 2H, 1-H), 1.73 (m, 2H, 2-H), 1.59 (d, 3H, *J*=6.4 Hz, 2'-H), 1.41 (s, 9H, CH_3 ^{t}Bu). ^{13}C NMR (75 MHz, $CDCl_3$): 171.8 (COO), 170.0 (COO), 169.4 (CON), 155.4 (OCON), 138.1, 135.8, 135.4, 129.6, 129.1, 129.0, 128.9, 128.7, 127.4, 127.2 (Ar), 80.8 (C ^{t}Bu), 67.8, 67.7 (OCH_2), 63.8 (Cα-Phe), 51.7 (C3), 49.7 (CHCl), 46.6 (C1), 34.8 (Cβ-Phe), 31.8 (C2), 28.6 (CH_3 ^{t}Bu), 21.6 (C2'). MS (ES)$^+$: 637.45 [M+H]$^+$.

N-Chloroacetyl-*N*-[(4*S*-Benzyloxycarbonyl-4-*tert*-butoxycarbonylamino)but-1-yl]-L-Phe-OBn (35)

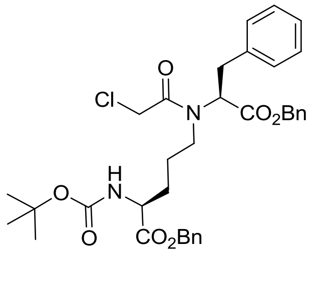

Syrup. Yield: 64% (from **25**). Eluent: EtOAc:Hexane (1:3). Roamers' ratio M:m=6:1. HPLC: t_R=17.68 min (gradient of 5% to 100% of A, in 20 min). ^{1}H NMR (300 MHz, $CDCl_3$, major rotamer): δ 7.38-7.12 (m, 15H, Ar), 5.18-5.04 (m, 4H, OCH_2), 4.86 (d, 1H, *J*=8.4 Hz, 4-NH), 4.18 (m, 1H, 4-H), 3.94 (d, 1H, *J*=12.5 Hz, CH_2Cl), 3.87 (d, 1H, *J*=12.5 Hz, CH_2Cl), 3.82 (dd, 1H, *J* = 8.3, 6.9 Hz, α-Phe), 3.36 (m, 2H, β-Phe), 3.12 (dt,

1H, J=15.4, 7.9 Hz, 1-H), 2.51 (dt, 1H, J=15.3, 8.1 Hz, 1-H), 1.61 (m, 1H, 3-H), 1.43 (m, 10H, ^{t}Bu, 3-H), 1.24 (m, 2H, 2-H). ^{13}C NMR (75 MHz $CDCl_3$): 172.0, 169.7 (COO), 166.5 (CON), 155.4 (OCON), 137.9, 135.6, 135.5, 129.5, 129.6, 128.9, 128.8, 128.75, 128.7, 128.6, 128.5, 128.4, 127.0 (Ar), 80.3 (C ^{t}Bu), 67.4, 67.3 (OCH_2), 63.5 (Cα-Phe), 52.7 (C4), 50.1 (CH_2Cl), 41.2 (C1), 34.4 (Cβ-Phe), 29.7 (C3), 28.4 (CH_3 ^{t}Bu), 24.3 (C2). MS $(ES)^+$: 637.27 $[M+H]^+$.

N-Chloroacetyl-*N*-[(4*S*-Benzyloxycarbonylamino-4-metoxycarbonyl)but-1-yl]-L-Phe-O^{t}Bu (36)

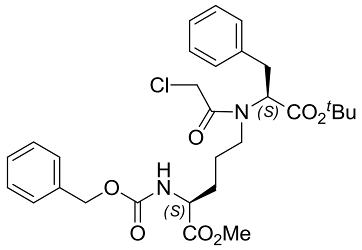

Syrup. Yield: 94% (from **26**). HPLC: t_R = 7.68 min (gradient of 30% to 95% of A, in 10 min). Roamers' ratio M,m = 2:1. ^{1}H NMR (300 MHz, $CDCl_3$, major rotamer): δ 7.36-7.13 (m, 10H, Ar), 5.27 (d, 1H, J=8.3 Hz, 4-NH), 5.10 (s, 2H, OCH_2), 4.27 (m, 1H, 4-H), 3.98 (m, 2H, CH_2Cl), 3.80 (m, 1H, α-Phe), 3.71 (s, 3H, OMe), 3.30 (m, 2H, β-Phe), 3.17 (m, 2H, 1-H), 3.61 (m, 1H, 1-H), 1.85 (m, 1H, 3-H), 1.68 (m, 3H, 3-H, 2-H), 1.45 (s, 9H, CH_3 ^{t}Bu). MS $(ES)^+$: 561.23 $[M+H]^+$.

N-Chloroacetyl-*N*-[4*S*-(N-benzyloxycarbonyl-N-methyl)amino-4-metoxycarbonyl]but-1-yl]-L-Phe-O^{t}Bu (37)

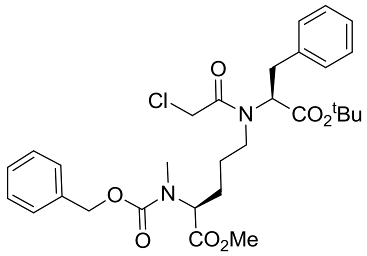

Syrup. Yield: 94% (from **27**). Eluent: EtOAc:Hexane (1:1). HPLC: t_R=16.21 min (gradient of 5% to 100% of A, in 20 min). Roamers' ratio M,m =5:2. ^{1}H NMR (400

MHz, $CDCl_3$, major rotamer): δ 7.38-7.12 (m, 10H, Ph), 5.16 (s, 2H, OCH_2), 4.68 (dd, 1H, *J*=10.9, 5.2 Hz, α-Phe), 4.39 (m, 1H, 4-H), 3.99 (d, 1H, *J*=12.2 Hz, CH_2Cl), 3.94 (d, 1H, *J*=12.5 Hz, CH_2Cl), 3.69 (s, 3H, OMe), 3.28 (m, 2H, 1-H), 2.89 (m, 1H, β-Phe), 2.77 (s, 3H, NCH_3), 2.52 (m, 1H, β-Phe), 1.75-1.60 (m, 4H, 2-H, 3-H), 1.44 (s, 9H, CH_3 ^{t}Bu). ^{13}C NMR (75 MHz, $CDCl_3$): 171.5 (COO), 168.9 (COO), 166.2 (CON), 157.1 (OCON), 138.4, 136.5, 129.4, 128.77, 128.7, 128.0, 126.8 (Ar), 82.0 (C ^{t}Bu), 67.8 (OCH_2), 64.2 (Cα-Phe), 57.6 (C4), 52.4 (OMe), 50.1 (CH_2Cl), 41.1 (C1), 34.4 (Cβ-Phe), 30.4 (NCH_3), 28.0 (CH_3 ^{t}Bu), 25.6 (C2), 24.9 (C3). MS (ES)$^+$: 576.42 [M+H]$^+$.

N-Chloroacetyl-*N*-[(4S-benzyloxycarbonylamino-4-metoxycarbonyl)but-1-yl]-L-Ala-O^{t}Bu (38)

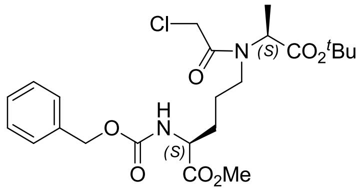

Syrup. Yield: 70% (from **28**). HPLC: t_R=6.18 min (gradient of 30% to 95% of A, en 10 min). Roamers' ratio M,m = 2:1 ^{1}H NMR (300 MHz, $CDCl_3$, major rotamer): δ 7.35 (s, 5H, Ph), 5.44 (br s, 1H, 4-NH), 5.11 (s, 2H, OCH_2), 4.40 (m, 1H, 4-H), 4.12 (s, 2H, CH_2Cl), 4.04 (m, 1H, α-Ala), 3.76 (s, 3H, OMe), 3.46 (m, 1H, 1'-H), 3.28 (m, 1H, 1'-H), 1.86 (m, 1H, 3'-H), 1.72 (m, 3H, 3'-H, 2'-H), 1.43 (s, 9H, CH_3 ^{t}Bu), 1.28 (d, 3H, *J*=6.3 Hz, β-Ala). MS (ES)$^+$: 485.16 [M+H]$^+$.

3. Characterization of β–lactam derivatives

4*R,S*-Benzyl-4-metoxycarbonyl-1-[(3'*S*-*tert*-butoxycarbonylamino-3'-methyloxycarbonyl)prop-1'-yl]-2-oxoazetidine (39). Syrup. Yield: 5% (from **29,** B:BTPP). Eluent: EtOAc:Hexane (1:1). HPLC: t_R=12.52, 12.65 min (gradient of 5% to 100% of A, in 20 min). HPLC (Chiral C column): t_R= 17.17, 18.55 min (Isocratic 9/91

(Acetone/Hexane). Diastereoisomers ratio M,m = 86:14. ^{1}H NMR (400 MHz, $CDCl_3$, major diastereosiomer): δ 7.33-7.12 (m, 5H, Ph), 5.19 (br s, 1H, 3'-NH), 4.26 (m, 1H, 3'-H), 3.76 (s, 3H, OMe), 3.74 (s, 3H, OMe), 3.15 (d, 1H, *J*=13.9 Hz, 4-CH_2), 3.23 (m, 2H, 1'-H), 3.18 (d, 1H, *J*=14.0 Hz, 4-CH_2), 3.14 (d, 1H, *J*=15.0 Hz, 3-H), 2.90 (d, 1H, *J*=15.0 Hz, 3-H), 2.24 (m, 1H, 2'-H), 2.02 (m, 1H, 2'-H), 1.45 (s, 9H, CH_3, ^{t}Bu). ^{13}C NMR (100 MHz, $CDCl_3$): 172.0 (COO), 166.5 (CON), 155.4 (OCON), 134.6, 129.8, 128.9, 128.8, 127.8 (C Ar), 80.3 (C ^{t}Bu), 63.1 (C4), 52.9 (OMe), 52.7 (OMe), 51.9 (C3'), 45.8 (C3), 40.0 (4-CH_2), 38.9 (C1'), 31.0 (C2'), 28.5 (CH_3 ^{t}Bu). MS $(ES)^+$: 435.32 $[M+H]^+$. Exact Mass calculated for $C_{22}H_{30}N_2O_7$: 434.20530; found: 434.20662.

4*R,S*-Benzyl-4-metoxycarbonyl-1-[(3'*S*-benzyloxycarbonyl-*3'-tert*-butoxycarbonylamino)prop-1'-yl]-2-oxoazetidine (40). Syrup. Yield: 56% (from **29,** B:BTPP). Eluent: EtOAc:Hexane (1:1). HPLC: t_R=17.22 min (gradient of 5% to 100% of A, in 20 min). HPLC (Chiral C column): t_R= 19.02, 22.12 min (Isocratic 9/91 (Acetone/Hexane). Diastereoisomers ratio M,m =80:20. ^{1}H NMR (400 MHz, $CDCl_3$, major diastereosiomer): δ 7.36-7.08 (m, 10H, Ph), 5.15 (br s, 3H, 3-NH, OCH_2), 4.26 (m, 1H, 3'-H), 3.71 (s, 3H, OMe), 3.26 (d, 1H, *J*=14.0 Hz, 4-CH_2), 3.20 (m, 2H, 1'-H), 3.13 (d, 1H, *J*=14.5 Hz, 4-CH_2), 3.12 (d, 1H, *J*=14.7 Hz, 3-H), 2.88 (d, 1H, *J*=14.7 Hz, 3-H), 2.23 (m, 1H, 2'-H), 1.97 (m, 1H, 2'-H), 1.43 (s, 9H, CH_3 ^{t}Bu). ^{13}C NMR (100 MHz, $CDCl_3$): 171.9 (COO), 166.4 (CON), 155.5 (OCON), 135.4, 134.5, 129.7, 128.9, 128.7, 128.6, 127.6 (C Ar), 80.1 (C ^{t}Bu), 67.4 (OCH_2), 63.0 (C4), 52.8 (OMe), 52.0 (C3'), 45.7 (C3), 39.7 (C1'), 38.7 (4-CH_2), 31.0 (C2'), 28.3 (CH_3 ^{t}Bu). MS $(ES)^+$: 511.25 $[M+H]^+$. Exact Mass calculated for $C_{28}H_{34}N_2O_7$: 510.2366; found: 510.2382.

4*R,S*-Benzyl-4-methoxycarbonyl-1-[(3'*S*-benzyloxycarbonylamino-3'-*tert*-butoxycarbonyl)prop-1'-yl]-2-oxoazetidine (42). Syrup. Yield: 52% (from **31**, B:Cs_2CO_3). Eluent: EtOAc:Hexane (1:1). HPLC: t_R= 9.06, 9.31 min (gradient of 5% to

100% of A, in 20 min). HPLC (Chiral C column): t_R= 22.22, 26.12 min (Isocratic 9/91 (Acetone/ Hexane). Diastereoisomers ratio M,m =90:10. ^{1}H NMR (400 MHz, $CDCl_3$, major diastereosiomer): δ 7.37-7.11 (m, 10H, Ph), 5.44 (brs, 1H, 3-NH), 5.13 (d, 1H, *J*=12.0 Hz, OCH_2), 5.09 (d, 1H, *J*=12.0 Hz, OCH_2), 4.20 (m, 1H, 3'-H), 3.74 (s, 3H, OMe), 3.22 (m, 3H, 1'-H, 4-CH_2), 3.16 (d, 1H, *J*=12.0 Hz, 4-CH_2), 3.14 (d, 1H, *J*=14.8 Hz, 3-H), 2.89 (d, 1H, *J*=14.8 Hz, 3-H), 2.23 (m, 1H, 2'-H), 2.04 (m, 1H, 2'-H), 1.46 (s, 9H, CH_3 ^{t}Bu).^{13}C NMR (75 MHz, $CDCl_3$): 171.9 (COO), 170.8 (COO), 166.9 (CON), 156.0 (OCON), 136.4, 129.75, 129.7, 128.9, 128.6, 128.3, 128.2, 127.7 (Ar), 82.6 (C ^{t}Bu), 67.0 (OCH_2), 63.1 (C4), 52.9 (OMe, C3'), 45.8 (C3), 39.9 (4-CH_2), 38.7 (C1'), 31.1 (C2'), 28.1 (CH_3 ^{t}Bu). MS (ES)$^+$: 511.55 [M+Na]$^+$. Exact Mass calculated for $C_{28}H_{34}N_2O_7$: 510.23660; found: 510.23824.

4*R*,*S*-Methyl-4-methoxycarbonyl-1-[(3'-benzyloxycarbonyl-3'*S*-*tert*-butoxycarbonylamino)prop-1'-yl]-2-oxoazetidine (43). Syrup. Yield: 25% (from **32**, B:BTPP). Eluent: EtOAc:Hexane (1:1). HPLC: t_R=12.64 min (gradient of 5% to 100% of A, in 20 min). Diastereoisomers ratio M,m =91:9. ^{1}H NMR (400 MHz, $CDCl_3$, major diastereosiomer): δ 7.35 (s, 5H, Ph), 5.24 (d, 1H, *J*=7.8 Hz, 3-NH), 5.17 (s, 2H, OCH_2), 4.30 (m, 1H, 3'-H), 3.74 (s, 3H, OMe), 3.26 (t, 2H, *J*=7.9 Hz, 1'-H), 3.18 (d, 1H, *J*=14.8 Hz, 3-H), 2.79 (d, 1H, *J*=14.8 Hz, 3-H), 2.20 (m, 1H, 2'-H), 2.00 (m, 1H, 2'-H), 1.43 (s, 12H, 4-CH_3 ^{t}Bu). ^{13}C NMR (75 MHz, $CDCl_3$): 172.8 (COO), 172.0 (COO), 166.3 (OCON), 135.4, 128.8, 128.6, 128.5 (Ar), 80.3 (C ^{t}Bu), 67.4 (OCH_2), 59.0 (C4), 58.9 (C3'), 52.8 (OMe), 48.9 (C3), 37.9 (C1'), 30.9 (C2'), 28.4 (CH_3 ^{t}Bu), 20.5 (4-CH_3). MS (ES)$^+$: 435.32 [M+H]$^+$. Exact Mass calculated for $C_{22}H_{30}N_2O_7$: 434.20531; found: 434.20519.

4*R*,*S*-Methyl-4-methoxycarbonyl-1-[(3'*S*-benzyloxycarbonylamino-3'-*tert*-butoxycarbonyl)prop-1'-yl]-2-oxoazetidine (44). Syrup. Yield: 31% (from **33**,

B:Cs_2CO_3). Eluent: EtOAc:Hexane (2:1). HPLC: t_R=7.84 min (gradient of 5% to 100% of A, in 20 min). Diastereoisomers ratio M,m= 69:31. ^{1}H NMR (400 MHz, $CDCl_3$, major diastereosiomer): δ 7.35 (s, 5H, Ph), 5.48 (d, 1H, *J*=7.9 Hz, 3-NH), 5.29 (s, 2H, OCH_2), 4.24 (m, 1H, 3'-H), 3.76 (s, 3H, OMe), 3.50 (m, 1H, 1'-H), 3.26 (m, 1H, 1'-H), 3.18 (d, 1H, *J*=14.9 Hz, 3-H), 2.78 (d, 1H, *J*=14.9 Hz, 3-H), 2.16 (m, 1H, 2'-H), 1.99 (m, 1H, 2'-H), 1.46 (s, 12H, CH_3 ^{t}Bu, 4-CH_3). ^{13}C NMR (75 MHz, $CDCl_3$): 172.7 (COO), 170.7 (COO), 166.1 (CON), 156.0 (OCON), 136.4, 128, 128.7, 128.25, 128.2 (C, Ar), 82.7 (C ^{t}Bu), 67.1 (OCH_2), 59.0 (C4), 52.9 (OMe, C3'), 48.9 (C3), 37.9 (C1'), 31.3 (C2'), 28.1 (CH_3 ^{t}Bu), 20.8 (4-CH_3). MS (ES)$^+$: 457.44 [M+Na]$^+$. Exact Mass calculated for $C_{22}H_{30}N_2O_7$: 434.20530; found: 434.20506.

4*R*,*S*-Benzyl-4-benzyloxycarbonyl-1-[(4'*S*-benzyloxycarbonyl-*4-tert*-butoxycarbonylamino)but-1'-yl]-2-oxoazetidine (46). Syrup. Yield: 71% (from **35**). Eluent: EtOAc:Hexane (1:4). HPLC: t_R=17.18 min (gradient of 5% to 100% of A, in 20 min). Diastereoisomers ratio M,m = 83:17. ^{1}H NMR (400 MHz, $CDCl_3$, major diastereoisomer): δ 7.40-6.90 (m, 15H, Ar), 5.17-4.95 (m, 5H, OCH_2, 4-NH), 4.22 (m, 1H, 4'-H), 3.24 (d, 1H, *J*=14.0 Hz, 4-CH_2), 3.23 (d, 1H, *J*=14.0 Hz, 4-CH_2), 3.11 (d, 1H, *J*=14.8 Hz, 3-H), 3.04 (m, 2H, 1'-H), 2.82 (d, 1H, *J*=14.8 Hz, 3-H), 1.60 (m, 2H, 3'-H), 1.52 (m, 2H, 2'-H), 1.36 (s, 9H, CH_3 ^{t}Bu). ^{13}C NMR (75 MHz, $CDCl_3$): δ 1725, 171.1 (COO), 166.4 (C2), 155.5 (OCON), 135.5, 134.9, 134.6, 129.7, 128.9, 128.8, 128.7, 128.7,128.5, 128.4, 127.6 (Ar), 80.0 (C ^{t}Bu), 67.7, 67.2 (OCH_2), 63.0 (C4), 53.4 (C4'), 45.8 (C3), 41.7 (C3), 40.1 (4-CH_2), 30.3 (C3'), 28.5 (CH_3 ^{t}Bu), 24.6 (C2'). MS (ES)$^+$: 623.09 [M+Na]$^+$. Exact Mass calculated for $C_{35}H_{40}N_2O_7$: 600.28355; found: 600.28414.

4*R*,*S*-Benzyl-N-[(4'S-benzyloxycarbonylamino-4'-methoxycarbonyl)but-1'-yl]-4-*tert*-butoxycarbonyl-2-oxoazetidine (47). Syrup. Yield: 78% (from **36**, B: Cs_2CO_3).

HPLC-MS: t_R=5.51 min (gradient of 50% a 95% of A, en 15 min). HPLC (Chiral C column): t_R=19.85, 22.39 min (Isocratic: 11/89, Acetone/Hexane), Diastereoisomers ratio M,m = 83:17. ^{1}H NMR (300 MHz, $CDCl_3$, major diastereoisomer): δ 7.35-7.07 (m, 10H, Ar), 5.46 (d, 1H, *J*=7.8, 4-NH), 5.04 (s, 2H, OCH_2), 4.30 (m, 1H, 4'-H), 3.67 (m, 3H, OCH_3), 3.22 (d, 1H, *J*=14.0, 4-CH_2), 3.13-3.01 (m, 3H, 1'-H, 4-CH_2, 3-H), 3.80 (d, 1H, *J*=14.7, 3-H), 1.80-1.56 (m, 4H, 2'-H, 3'-H), 1.35 (s, 9H, CH_3 ^{t}Bu). ^{13}C NMR (100 MHz, $CDCl_3$): major isomer 172.7 (COO), 170.4 (COO), 166.9 (C2), 156.1 (OCON), 136.4, 135.0, 129.8, 128.8, 128.6, 128.3, 128.2, 127.5 (Ar), 83.1 (C ^{t}Bu), 67.1 (OCH_2), 63.5 (C4), 53.7 (C4'), 52.6 (OMe), 45.9 (C3), 41.5 (C1'), 40.0 (4-CH_2), 29.8 (C3'), 28.0 (CH_3 ^{t}Bu), 24.6 (C2'). MS (ES)$^+$: 525.24 $[M+H]^+$.

4*R*,*S*-Benzyl-1-[4'*S*-(*N*-benzyloxycarbonyl-*N*-methyl)amino-4'-methoxycarbonyl]but-1-yl]-4-*tert*-butoxycarbonyl-2-oxoazetidine (48). Syrup. Yield: 77% (from **37**, B: BTPP). Eluent: EtOAc:Hexane (1:1). HPLC: t_R=16.26 min (gradient of 5% to 100% of A, in 20 min). HPLC (Chiral C column): t_R= 16.82, 17.83 min (Isocratic 10/90 Acetone/Hexane). Diastereoisomers ratio M,m = 71:29. Roamers' ratio M,m =60:40. ^{1}H NMR (400 MHz, $CDCl_3$, major rotamer): δ 7.36-7.13 (m, 10H, Ar), 5.15 (s, 2H, OCH_2), 4.79 (dd, 1H, *J*=10.1, 4.4 Hz , 4'-H), 3.71 (s, 3H, OMe), 3.29 (d, 1H, *J*=15.1 Hz, 4-CH_2), 3.25-3.10 (m, 4H, 1'-H, 3-H, 4-CH_2), 3.06 (d, 1H, *J*=14.0 Hz, □-Ḥ 2.85 (s, 3H, NMe), 1.94-1.71 (m, 4H, 2'-H, 3'-H), 1.41 (s, 9H, CH_3 ^{t}Bu). ^{13}C NMR (75 MHz, $CDCl_3$): 171.8 (COO), 170.3 (COO), 166.9 (CON), 157.1 (OCON), 136.7, 135.1, 129.8, 128.7, 128.6, 128.1, 128.0, 127.9, 127.5 (Ar), 83.0 (C ^{t}Bu), 67.6 (OCH_2), 63.4 (C4), 58.4 (C4'), 52.3 (OMe), 45.8 (C3), 41.4 (C1'), 40.0 (4-CH_2), 30.7 (NMe), 27.9 (CH_3 ^{t}Bu), 26.6 (C3'), 25.5 (C2'). MS (ES)$^+$: 539.67 $[M+H]^+$. Exact Mass calculated for $C_{30}H_{38}N_2O_7$: 538.26790; found: 538.26910.

4*R,S*-Benzyl-1-[4'*S*-[(*N*-methyl)amino-4'-methoxycarbonyl]but-1'-yl]-4-*tert*-butoxycarbonyl-2-oxoazetidine (49). A solution of **48** (0.15 mmol, 82 mg) in MeOH (15 mL) was hydrogenated at room temperature and 15 psi of pressure for 4 h, using 10% Pd-C as catalyst. After filtration of the catalyst, the solvent was evaporated, and the resulting residue was purified on a silica gel column using MeOH:DCM (1:10). Syrup. Yield: (48 mg, 78%). HPLC: t_R=8.87 min (gradient of 5% to 100% of A, in 20 min). Diastereoisomers ratio M,m=73:27. ^{1}H NMR (400 MHz, $CDCl_3$, major diastereosiomer): δ 7.31-7.17 (m, 5H, Ph), 3.74 (s, 3H, OMe), 3.32 (d, 1H, *J*=13.9 Hz, 4-CH_2), 3.21 (m, 3H, 1'-H, 4'-H), 3.16 (d, 1H, *J*=14.8 Hz, 3-H), 3.10 (d, 1H, *J*=13.9 Hz, 4-CH_2), 2.86 (d, 1H, *J*=14.8 Hz, 3-H), 2.39 (s, 3H, NMe), 1.69 (m, 4H, 3'-H, 2'-H), 1.42 (s, 9H, CH_3 ^{t}Bu). ^{13}C NMR (75 MHz, $CDCl_3$): 174.8 (COO), 169.8 (COO), 166.4 (CON), 134.6, 129.3, 128.2, 127.0 (Ar), 82.5 (C ^{t}Bu), 62.9 (C4), 62.3 (C4'), 51.4 (OMe), 45.3 (C3), 41.2 (C1'), 39.7 (4-CH_2), 34.3 (NMe), 30.3 (C3'), 27.5 (CH_3 ^{t}Bu), 24.7 (C2'). MS $(ES)^+$: 405.49 $[M+Na]^+$. Exact Mass calculated for $C_{22}H_{32}N_2O_5$: 404.23112; found: 404.23245.

4'*S*-N-[(4'-Benzyloxycarbonylamino-4'-methoxycarbonyl)but-1'-yl]-4*R,S*-*tert*-butyloxycarbonyl-4-methyl-2-oxoazetidine (50). Syrup. Yield: 58% (from **38**, B: Cs_2CO_3). HPLC-MS: t_R=3.06 min (gradient of 50% to 95% of A, in 15 min). Diastereoisomers ratio M, m = 67:33. ^{1}H NMR (300 MHz, $CDCl_3$): major isomer δ 7.37-7.31 (m, 5H, Ph), 5.54 (d, 1H, *J*= 7.1 Hz, 4-NH), 5.11 (s, 2H, OCH_2), 4.37 (m, 1H, 4'-H), 3.75 (s, 3H, OMe), 3.20 (m, 2H, 1'-H), 3.14 (d, 1H, *J*=14.5 Hz, 3-H), 2.76 (d, 1H, *J*=14.5 Hz, 3-H), 1.90 (m, 1H, 3'-H), 1.80-1.63 (m, 3H, 3'-H, 2'-H), 1.59 (s, 3H, 4-CH_3), 1.45 (s, 9H, CH_3 ^{t}Bu). MS $(ES)^+$: 449.16 $[M+H]^+$, 471.46 $[M+Na]^+$.

Hydrogenolysis of benzyl esters to free carboxylic acid derivatives.

A solution of the corresponding 4-benzylcarbonyl β–lactam derivative (0.15 mmol) in MeOH (15 mL) was hydrogenated at room temperature and 15 psi of pressure for 4 h, using 10% Pd-C as catalyst. After filtration of the catalyst, the solvent was evaporated and the reaction product was characterized without further purification.

4*R*,*S*-Benzyl-4-carboxy-1-[3'*S*-tert-butoxycarbonylamino-3'-carboxy)prop-1'-yl]-2-oxoazetidine (51). Syrup. Yield: 99% (from **41**). HPLC: t_R=9.17 min (gradient of 5% to 100% of A, in 20 min). Diastereoisomers ratio M,m = 60:40. ^{1}H NMR (400 MHz, $CDCl_3$, major diastereoisomer): δ 9.40 (s, 2H, OH), 7.38-7.06 (m, 5H, Ph), 5.68 (d, 1H, *J*=8.5 Hz, 3'-NH), 4.24 (m, 1H, 3'-H), 3.54-2.73 (m, 6H, 1'-H, 3-H, 4-CH_2), 2.17 (m, 2H, 2'-H), 1.43 (s, 9H, ^{t}Bu). ^{13}C NMR (75 MHz, $CDCl_3$): δ 175.7, 174.6 (COO), 168.3 (C2), 156.3 (OCON), 134.1, 130.0, 128.9, 127.7, (Ar), 80.8 (C ^{t}Bu), 63.4 (C4), 51.8 (C3'), 44.8 (C3), 38.8 (C1', 4-CH_2), 30.6 (C2'), 28.4 (CH_3 ^{t}Bu). MS $(ES)^+$: 407.20 $[M+H]^+$.

4*R*,*S*-Benzyl-1-[4'*S*-tert-butoxycarbonylamino-4'-carboxy)but-1'-yl]-4-carboxy-2-oxoazetidine (53). Syrup. Yield: 74% (from **46**). HPLC: t_R = 9.46 min (gradient of 5% to 100% of A, in 20 min). Diastereoisomers ratio M,m = 60:40. ^{1}H NMR (300 MHz, $CDCl_3$, major diastereoisomer): δ 10.09 (s, 2H, OH), 7.24-7.07 (m, 5H, Ar), 5.41 (d, 1H, *J*=8.1 Hz, 4'-NH), 4.18 (m, 1H, 4'-H), 3.50-2.62 (m, 6H, 1'-H, 3-H, 4-CH_2), 1.88-1.56 (m, 4H, 3'-H, 2'-H), 1.36 (s, 9H, ^{t}Bu). ^{13}C NMR (75 MHz, $CDCl_3$): δ 176.2, 174.5 (COO), 168.1 (C2), 156.1 (OCON), 134.6, 129.9, 128.8, 127.5 (Ar), 80.6 (C ^{t}Bu), 63.1 (C4), 53.1 (C4'), 45.1 (C3), 41.9 (C1'), 38.9 (4-CH_2), 29.9 (C3'), 28.4 (CH_3 ^{t}Bu), 24.0 (C2'). MS $(ES)^+$: 421.26 $[M+H]^+$.

Synthesis of dipeptide derivatives.

A solution of the corresponding 4-carboxy β–lactam derivative (0.33 mmol) and H-L-Ala-OMe, or H-L-Phe-OMe, or H-D-Ala-OMe (0.66 or 0.33 mmol) in dry THF (4 mL)

was successively treated with PyBOP (0.66 mmol, 0.34g) and TEA (0.18 mL, 1.32 mmol) at room temperature. The stirring was continued until complete disappearance of the starting material (1-2 days). The isomers ratio was determined by HPLC on the crude reaction mixtures. To characterize the obtained dipeptide derivatives, the solvent was evaporated, and the residue was dissolved in EtOAc and washed with citric acid (10%), $NaHCO_3$ (10%), and brine. The organic layer was dried (Na_2SO_4) and evaporated, leaving a residue which was purified on a silica gel column or by semipreparative HPLC, as specified in each case.

4*R*-Benzyl-1-[3'S-*tert*-butoxycarbonylamino-3'-[*N*-[(1''*S*-methoxycarbonyl-2'-phenyl)ethyl]carbamoyl]prop-1'-yl]-4-[*N*-[(1'''*S*-methoxycarbonyl-2'phenyl)ethyl]carbamoyl]-2-oxoazetidine (56a). Syrup. Yield: 32% (from **51** and H-L-Phe-OMe). Purificated by semipreparative HPLC (gradient of 50% to 60% of A, in 30 min). [α_D] = +14.92. Purificated by semipreparative HPLC (gradient of 50% a 60% of A, en 30 min). HPLC: t_R=15.47 min (gradient of 5% to 100% of A, in 20 min). ^{1}H NMR (400 MHz, $CDCl_3$): δ 8.44 (d, 1H, *J*= 7.6 Hz, NHCO), 7.56 (d, 1H, *J*= 7.6 Hz, NHCO), 7.32-7.14 (m, 15H, Ar), 5.66 (d, 1H, *J*= 7.1 Hz, 3'-NH), 5.04 (m, 1H, 1''-H), 4.76 (m, 1H, 1'''-H), 4.29 (m, 1H, 3'-H), 3.73 (s, 3H, OMe), 3.70 (s, 3H, OMe), 3.63 (m, 2H, 1'-H), 3.38 (m, 2H, 4-CH_2), 3.32 (m, 2H, 2''-H), 3.16 (dd, 1H, *J*=13.9, 5.8 Hz, 2'''-H), 3.04 (m, 1H, 1'-H), 2.96 (dd, 1H, *J*=13.5, 8.2 Hz, 2'''-H), 2.60 (dd, 1H, *J*=14.8 Hz, 3-H), 2.10 (dd, 1H, *J*=14.8 Hz 3-H), 1.92 (m, 2H, 2'-H), 1.41 (s, 9H, CH_3 ^{t}Bu). ^{13}C NMR (75 MHz, $CDCl_3$): 172.2 (COO), 171.8 (COO), 171.3 (CON), 171.1 (CON), 169.7 (C2), 155.65 (OCON), 137.6, 136.4, 135.00, 130.3, 129.4, 129.3, 128.85, 128.7, 128.6, 127.4, 127.15, 127.0 (C, Ar), 79.9 (C ^{t}Bu), 63.45 (C4), 54.2, 53.4 (C1'' and C1'''), 52.5 (OMe), 52.4 (OMe), 51.9 (C3'), 46.9 (C3), 40.2 (C1'), 37.9 (4-CH_2), 36.6,

36.4 (C2'' and C2'''), 33.4 (C2'), 28.5 (CH_3 ^{t}Bu). MS (ES)$^+$: 729.53 [M+H]$^+$. Exact Mass calculated for $C_{40}H_{48}N_4O_9$: 728.34213; found: 728.34220.

4*S*-Benzyl-1-[3'S-*tert*-butoxycarbonylamino-3'-[*N*-[(1''*S*-methoxycarbonyl-2'-phenyl)ethyl]carbamoyl]prop-1'-yl]-4-[*N*-[(1'''*S*-methoxycarbonyl-2'phenyl)ethyl]carbamoyl]-2-oxoazetidine (56b). Syrup. Yield: 12% (from **51** and H-L-Phe-OMe). [α_D] = +160.34. HPLC: t_R=15.13 min (gradient of 5% to 100% of A, in 20 min). ^{1}H NMR (400 MHz, $CDCl_3$): δ 7.49 (m, 2H, NHCO), 7.28-7.14 (m, 15H, Ar), 5.38 (d, 1H, *J*= 6.6 Hz, 3'-NH), 4.91(m, 1H, 1''-H), 4.83(m, 1H, 1'''-H), 4.33 (m, 1H, 3'-H), 3.75 (s, 3H, OMe), 3.69 (s, 3H, OMe), 3.34 (m, 2H, 1'-H), 3.31 (m, 2H, 2''-H), 3.17 (m, 2H, 2'''-H, 4-CH_2), 3.07 (m, 2H, 2'''-H, 4-CH_2), 2.93 (m, 1H, 1'-H), 2.76 (d, 1H, *J*=14.7 Hz, 3-H), 2.46 (d, 1H, J=14.7 Hz, 3-H), 1.86 (m, 1H, 2'-H), 1.60 (m, 1H, 2'-H), 1.43 (s, 9H, CH_3 ^{t}Bu). ^{13}C NMR (75 MHz, $CDCl_3$): 172.7 (COO), 171.8 (COO), 171.6 (CON), 167.4 (C2), 155.8 (OCON), 136.3, 136.2, 130.1, 129.4, 129.3, 128.85, 128.8, 128.7, 127.4, 127.3, 127.2 (C, Ar), 80.3 (C ^{t}Bu), 63.3 (C4), 53.6 (C1'' and C1'''), 52.8 (OMe), 52.4 (OMe), 52.0 (C3'), 47.5 (C3), 38.7 (4-CH_2), 38.1 (C1'), 37.9, 37.3 (C2'' and C2'''), 31.3 (C2'), 28.4 (CH_3 ^{t}Bu). MS (ES)$^+$: 729.53 [M+H]$^+$. Exact Mass calculated for $C_{40}H_{48}N_4O_9$: 728.34213; found: 728.34342.

4*R*-Benzyl-1-[3'*S*-*tert*-butoxycarbonylamino-3'-[*N*-[(1''*S*-methoxycarbonyl)ethyl]carbamoyl]prop-1'-yl]-4-[*N*-[(1'''*S*-methoxycarbonyl) ethyl]carbamoyl]-2-oxoazetidine (57a). Syrup. Yield: 28% (form **51** and H-L-Ala-OMe). Eluent: MeOH:DCM (1:30). HPLC: t_R= 12.65 min (gradient of 15% to 95% of A, in 5 min). [α_D] = 17.98. ^{1}H NMR (400 MHz, $CDCl_3$): δ 8.38 (d, 1H, *J*=7.3 Hz, 1'''-NH), 7.53 (d, 1H, *J*= 7.5 Hz, 1''-NH), 7.31-7.18 (m, 15H, Ar), 5.67 (d, 1H, *J*=7.4 Hz, 3'-NH), 4.70 (q, 1H, *J*= 7.5 Hz, 1'''-H), 4.52 (q, 1H, *J*=7.3 Hz, 1''-H), 4.34 (m, 1H, 3'-H), 3.72 (s, 4H, OMe, 1'-H), 3.69 (s, 3H, OMe), 3.48 (d, 1H, *J*=14.8 Hz, 4-CH_2), 3.38

(d, 1H, *J*=14.8 Hz, 4-CH_2), 3.18 (m, 1H, 1'-H), 2.98 (d, 1H, *J*=15.1 Hz, 3-H), 2.90 (d, 1H, *J*=15.1 Hz, 3-H), 2.22 (m, 1H, 2'-H), 2.04 (m, 1H, 2'-H), 1.52 (d, 3H, *J*=7.2 Hz, 2'''-H), 1.38 (d, 3H, *J*=7.3 Hz, 2''-H), 1.37 (s, 9H, CH_3 ^{t}Bu). ^{13}C NMR (75 MHz, $CDCl_3$): 173.4 (COO), 172.9 (COO), 171.1 (CON'''), 170.8 (CON''), 169.6 (C2), 155.7 (OCON), 135.0, 130.4, 128.9, 127.5 (Ar), 79.9 (C ^{t}Bu), 63.6 (C4), 52.6 (OMe), 52.55 (OMe), 52.5 (C3'), 48.7 (C1'''), 48.3 (C1''), 46.9 (C3), 40.3 (C1'), 37.2 (4-CH_2), 33.6 (C2'), 28.5 (CH_3 ^{t}Bu), 17.7 (C2''), 17.1 (C2'''). MS (ES)$^+$: 577.47 [M+H]$^+$. Exact Mass calculated for $C_{28}H_{40}N_4O_9$: 576.27953; found: 576.27722.

4*S*-Benzyl-1-[3'*S*-*tert*-butoxycarbonylamino-3'-[*N*-[(1''*S*-methoxycarbonyl)ethyl]carbamoyl]prop-1'-yl]-4-[*N*-[(1'''*S*-methoxycarbonyl) ethyl]carbamoyl]-2-oxoazetidine (57b). Syrup. Yield: 12% (form **51** and H-L-Ala-OMe). Eluent: MeOH:DCM (1:30). HPLC: t_R=12.62 min (gradient of 15% to 95% of A, in 5 min). From a mixture of diastereoisomers M,m =59:41. ^{1}H NMR (400 MHz, $CDCl_3$, major diastereoisomer): δ 7.62 (sa, 1H, NHCO), 7.31-7.15 (m, 15H, Ar), 7.04 (sa, 1H, NHCO), 5.43 (d, 1H, *J*= 6.6 Hz, 3'-NH), 4.52 (m, 2H, 1''-H, 1'''-H), 4.34 (m, 1H, 3'-H), 3.73 (s, 3H, OMe), 3.71 (s, 3H, OMe), 3.52 (m, 1H, 1'-H), 3.38 (m, 2H, 4-CH_2), 3.25 (m, 1H, 1'-H), 3.17 (m, 1H, 1'-H), 3.02 (d, 1H, *J*=14.7 Hz, 3-H), 2.97 (d, 1H, *J*=14.7 Hz, 3-H), 2.05 (m, 1H, 2'-H), 1.84 (m, 1H, 2'-H), 1.41 (s, 9H, CH_3 ^{t}Bu), 1.40 (d, 3H, *J*=6.8 Hz, 2'''-H), 1.32 (d, 3H, *J*=7.2 Hz, 2''-H). ^{13}C NMR (75 MHz, $CDCl_3$): 173.6 (COO), 173.1 (COO), 171.2 (CON), 171.1 (CON), 167.4 (C2), 155.7 (OCON), 134.9, 130.0, 128.9, 127.5 (Ar), 80.2 (C^{t}Bu), 63.6 (C4), 52.8 (OMe), 52.5 (OMe), 52.4 (C3'), 48.5 (C1'''), 48.3 (C1''), C1'''), 47.1 (C3), 39.1 (C1'), 37.1 (4-CH_2), 32.1 (C2'), 28.4 (CH_3 ^{t}Bu), 18.1 (C2''), 17.6 (C2'''). MS (ES)$^+$: 577.47 [M+H]$^+$.

4*R*,*S*-Benzyl-1-[(4'*S*-benzyloxycarbonylamino-4'-methoxycarbonyl)but-1'-yl]-4-[N-[(1''*S*-methoxycarbonyl)ethyl]carbamoyl]-2-oxoazetidine (58). Syrup. Yield: 27%

(from **47**, by treatment with TFA (to **54**), and subsequent coupling with H-L-Ala-OMe). HPLC-MS: t_R=12.57 min (gradient of 2% to 95% of A, in 15 min). Diatereoisomers ratio M,m=82:18. ^{1}H NMR (300 MHz, $CDCl_3$, major diastereoisomer): δ 7.10-7.27 (m, 10H, Ar), 6.30 (d, 1H, *J*=7.1 Hz, 4'-NH), 5.56 (d, 1H, *J*=8.0 Hz, 1''-NH), 5.03 (s, 2H, OCH_2), 4.48 (quint, 1H, *J*=7.1 Hz , 4'-H), 4.30 (m, 1H, 1"-H), 3.67 (m, 3H, OCH_3), 3.63 (m, 3H, OCH_3), 3.22-3.07 (m, 4H, 4-H, 1'-H), 2.91 (s, 2H, 4-CH_2), 1.79-1.60 (m, 4H, 2'-H, 3'-H), 1.38 (d, 3H, *J*=7.2 Hz , 2"-H, m), 1.25 (d, 3H, *J*=7.2 Hz , 2"-H, M). MS (ES)$^+$: 554.19 [M+H]$^+$.

4*R,S*-Benzyl-1-[(4'*S*-benzyloxycarbonylamino-4'-methoxycarbonyl)but-1'-yl]-4-[N-[(1''*R*-methoxycarbonyl)ethyl]carbamoyl]-2-oxoazetidine (59). Syrup. Yield: 44% (from **47**, by treatment with TFA (to **54**), and subsequent coupling with H-D-Ala-OMe). HPLC-MS: t_R=12.17 min (gradient of 2% to 95% of A, in 15 min). Diatereoisomers ratio M,m=67:33. ^{1}H NMR (300 MHz, $CDCl_3$): δ 7.27-7.09 (m, 10H, Ar), 6.44 (d, 1H, *J*=6.9 Hz, 4'-NH, M), 6.36 (d, 1H, *J*=7.0 Hz, 4'-NH, m), 5.50 (d, 1H, *J*=8.1 Hz, 1''-NH), 5.03 (s, 2H, OCH_2), 4.45 (m, 1H, 4'-H), 4.29 (m, 1H, 1"-H), 3.67 (s, 3H, OCH_3), 3.64 (s, 3H, OCH_3), 3.84-3.34 (m, 6H, 1'-H, 3-H, 4-CH_2), 1.90-1.50 (m, 4H, 2'-H, 3'-H), 1.32 (d, 3H, *J*=7.2 Hz , 2"-H, M), 1.31 (d, 3H, *J*=7.2 Hz , 2"-H, m). MS (ES)$^+$: 554.11 [M+H]$^+$.

4*R,S*-Benzyl-1-[4'*S*-[(*N*-benzyloxycarbonyl-*N*-methyl)amino-4'-methoxycarbonyl]but-1'-yl]-4-[N-[(1''*S*-metoxycarbonyl)ethyl]carbamoyl]-2-oxoazetidine (60). Syrup. Yield: 33% (from **48**, by treatment with TFA (to **55**), and subsequent coupling with H-L-Ala-OMe). Purified by semipreparative HPLC (gradient of 50% to 60% of A, in 30 min). HPLC: t_R = 9.51 min (gradient of 50% to 60% of A, in 15 min). Diatereoisomers ratio M,m = 74:26. Roamers' ratio M,m= 2:1 ^{1}H NMR (400 MHz, $CDCl_3$): δ 7.35-7.15 (m, 10H, Ph, Z), 6.31 (d, 1H, *J*=7.4 Hz, NHCO, M), 6.20 (d,

1H, *J*=7.4 Hz, NHCO, m), 5.14 (s, 2H, OCH_2), 4.81 (dd, 1H, *J*=9.8, 5.0 Hz, 4'-H), 4.56 (m, 1H, 1''-H), 3.72 (s, 3H, OMe, M), 3.71 (s, 3H, OMe, M), 3.64 (s, 3H, OMe, m), 3.37 (d, 1H, *J*=14.0 Hz, 3-H), 3.25 (m, 2H, 4-CH_2), 3.20 (d, 1H, *J*=14.0 Hz, 3-H), 2.98 (s, 2H, 1'-H), 2.86 (s, 3H, NCH_3), 1.94 (m, 1H, 2'H), 1.76 (m, 3H, 3'-H, 2'-H), 1.32 (d, 3H, *J*=7.1 Hz, 2''-H, M), 1.29 (d, 3H, *J*=7.1 Hz, 2''-H, m). ^{13}C NMR (75 MHz, $CDCl_3$): 173.1 (COO), 171.9 (COO, M), 171.2 (COO, m), 170.65 (CON, m), 170.6 (CON, m), 166.5 (C2, M), 166.3 (C2, m), 157.0 (OCON, M), 156.9 (OCON, m), 136.7, 135.2, 135.1, 130.0, 129.9, 129.0, 128.9, 128.7, 128.6, 128.2, 128.1, 127.9, 127.7, 127.6 (Ar), 67.7 (OCH_2, M), 67.6 (OCH_2, m), 63.7 (C4), 58.7 (C4', m), 58.3 (C4', M), 52.8 (OMe, m) 52.7 (OMe, M), 52.3 (OMe, M), 52.2 (OMe, m), 48.4 (C1'', M), 48.3 (C1'', m), 46.9 (C3, m), 46.7 (C3, M), 42.05 (C1', m), 42.0 (C1', M), 39.9 (4-CH_2, m), 39.8 (4-CH_2, M), 31.4 (NMe, m), 30.6 (NMe, M), 26.9 (C3', m), 26.5 (C3', M), 25.4 (C2', m), 25.2 (C2', M), 18.0 (C2''). MS (ES)$^+$: 590.40 [M+Na]$^+$.

4*R*,*S*-Benzyl-1-[(4'*S*-methylamino-4'-metoxycarbonyl)but-1'-yl]-4-[N-[(1''*S*-methoxycarbonyl)ethyl]carbamoyl]-2-oxoazetidine (61). A solution of compound **60** (0.2 mmol, 87 mg) in MeOH (15 mL) was hydrogenated at room temperature and 15 psi of pressure for 4 h, using 10% Pd-C as catalyst. After filtration of the catalyst, the solvent was evaporated, and the resulting residue was syrup. Yield: 78%. HPLC: t_R= 7.95 min (gradient of 2% to 95% of A, in 15 min). Diastereoisomers ratio M,m=73:27. ^{1}H NMR (400 MHz, $CDCl_3$): δ 7.31-7.17 (m, 5H, Ph), 6.64 (d, 1H, *J*=7.4 Hz, NHCO, M), 6.55 (d, 1H, *J*=7.4 Hz, NHCO, m), 4.56 (m, 2H, 4'-H, 1''-H,), 3.75 (s, 3H, OMe, M), 3.74 (s, 3H, OMe, m), 3.73 (s, 6H, OMe), 3.40 (d, 1H, *J*=14.2 Hz, 4-CH_2 M), 3.26 (m, 3H, 1'-H, 4-CH_2), 3.23 (d, 1H, *J*=14.1 Hz, 4-CH_2 M), 3.17 (m, 1H, 1'-H), 3.08 (d, 1H, J = 14.7 Hz, 3-H, m), 2.96 (s, 2H, 3-H M), 2.93 (d, 1H, J = 14.7 Hz, 3-H, m), 2.36 (s, 3H, NCH_3, m), 2.35 (s, 3H, NCH_3, M), 1.85-1.73 (m, 2H, 2'-H), 1.71-1.58 (m, 2H,

3’-H), 1.41 (d, 3H, *J*=7.2 Hz, 2’’-H, m), 1.33 (d, 3H, *J*=7.2 Hz, 2’’-H, M). ^{13}C NMR (75 MHz, $CDCl_3$): 175.1 (COO, M), 174.9 (COO, m), 173.1 (COO, M), 171.0 (COO, m), 171.0 (CON, m), 170.8 (CON, M), 166.8 (C2, m), 166.6 (C2, M), 135.0, 129.9, 129.8, 128.7, 128.6, 127.4 (Ar), 63.6 (C4, m), 63.5 (C4, M), 62.6 (C4’, m), 62.4 (C4’, M), 52.7 (OMe), 52.0 (OMe, m), 51.9 (OMe, M), 48.6 (C1’’, m), 48.4 (C1’’, M), 46.9 (C3, M), 46.8 (C3, m), 42.0 (C1’, m), 41.9 (C1’, M), 39.3 (4-CH_2, m), 39.3 (4-CH_2, M), 34.7 (NMe, m), 34.6 (NMe, M), 30.7 (C3’, M), 30.5 (C3’, m), 25.0 (C2’, m), 24.9 (C2’,M), 18.1 (C2’’, m), 18.0 (C2’’, M). MS $(ES)^+$: 434.38 $[M+H]^+$.

Synthesis of β-lactam amide derivatives.

PyBOP (0.62 mmol, 0.32 g), TEA (0.62 mmol, 0.086 mL) and the corresponding amine (0.62 mmol) were added to a solution of the carboxylic acid derivative **51-53** (0.155 mmol) in dry CH_2Cl_2 (2 mL) or dry DMF (2 mL). The reaction mixture was stirred overnight. The solvent was removed and the residue was extracted with EtOAc and washed with HCl 0.1N and then, the aqueous phase was basified with NaOH 1M and the solution was extracted with EtOAc and washed with citric acid (10%), $NaHCO_3$ (10%) and brine. Finally, the organic phase was dried over dry Na_2SO_4, filtered, and concentrated. The residue was purified by flash chromatography on silica gel, using the eluent mixture indicated in each case.

4*R*-Benzyl-4-[(N-Benzyl)carbamoyl]-1-[(3’*S*-*tert*-butoxycarbonylamino-3’-[(N-Benzyl)carbamoyl]prop-1’-yl]-2-oxoazetidine (62a). Syrup. Yield: 80% (from **51** and benzylamine). Eluent: EtOAc:Hexane (2:1). HPLC: t_R=13.79 min (gradient of 5% to 100% of A, in 20 min). ^{1}H NMR (400 MHz, $CDCl_3$): δ 7.62 (br s, 1H, NHCO), 7.31-7.12 (m, 15H, Ar), 6.11 (br s, 1H, NHCO), 5.33 (br s, 1H, 3’-NH), 4.44 (m, 2H, NCH_2), 4.40 (dd, 1H, *J*=14.4, 5.7 Hz, NCH_2), 4.30 (dd, 1H, *J*=14.4, 5.7 Hz, NCH_2), 4.23 (m, 1H, 3’-H), 3.47 (m, 1H, 1’-H), 3.39 (d, 1H, *J*=14.0 Hz, 4-CH_2), 3.32 (m, 2H, 1’-H, 4-

CH_2), 3.04 (d, 1H, *J*=14.6 Hz, 3-H), 2.95 (d, 1H, *J*=14.6 Hz, 3-H), 2.05 (m, 1H, 2'-H), 1.94 (m, 1H, 2'-H), 1.42 (s, 9H, CH_3 ^{t}Bu). ^{13}C NMR (75 MHz, $CDCl_3$): 171.5 (CON), 170.8 (CON), 167.4 (C2), 155.5 (OCON), 138.1, 137.4, 129.8, 128.95, 128.9, 128.8, 128.3, 127.95, 127.9, 127.6 (Ar), 81.0 (C ^{t}Bu), 64.2 (C4), 52.4 (C3'), 46.4 (C3), 44.0, 43.7 ($NHCH_2$), 40.6 (C1'), 39.3 (4-CH_2), 29.9 (C2'), 28.5 (CH_3 ^{t}Bu). MS (ES)$^+$: 585.55 $[M+H]^+$. Exact Mass calculated for$C_{34}H_{40}N_4O_5$: 584.29987; found: 584.30012.

4*R*-4-Benzyl-4-[(N-Benzyl)carbamoyl]-3*R*-methyl-1-[3'*S*-*tert*-butoxycarbonylamino-3'-[(N-Benzyl)carbamoyl]prop-1'-yl]-2-oxoazetidine (63a). Syrup. Yield: 55% (from **52** and benzylamine). Eluent: Acetone:DCM (1:25). [α_D] = -38.35. HPLC: t_R=14.18 min (gradient of 5% to 100% of A, in 20 min). ^{1}H NMR (400 MHz, $CDCl_3$): δ 8.32 (m, 1H, NHCO), 8.20 (m, 1H, NHCO), 7.45-7.19 (m, 15H, Ar), 5.99 (d, 1H, *J*=6.6 Hz, 3'-NH), 4.53 (m, 5H, NCH_2, 3'-H), 3.69 (d, 1H, *J*=14.9 Hz, 4-CH_2), 3.35 (m, 2H, 1'-H, 4-CH_2), 3.10 (m, 1H, 1'-H), 3.04 (q, 1H, *J*=7.5 Hz, 3-H), 2.30 (m, 1H, 2'-H), 1.94 (m, 1H, 2'-H), 1.39 (s, 9H, CH_3 ^{t}Bu), 1.01 (d, 3H, *J*=7.5 Hz, 3-CH_3).^{13}C NMR (75 MHz, $CDCl_3$): 173.2 (CON), 171.0 (CON), 170.0 (C2), 156.5 (OCON), 138.9, 138.2, 135.7, 130.2, 128.9, 128.7, 128.4, 128.2, 127.7, 127.4, 127.3, 127.1 (Ar), 80.2 (C ^{t}Bu), 69.1 (C4), 53.5 (C3), 51.9 (C3'), 43.8, 43.6 (NCH_2), 41.4 (C1'), 38.6 (4-CH_2), 34.1 (C2'), 28.4 (CH_3 ^{t}Bu), 9.6 (3-CH_3). MS (ES)$^+$: 599.31 $[M+H]^+$. Exact Mass calculated for $C_{35}H_{42}N_4O_5$: 598.31552; found: 598.31608.

4*S*-4-Benzyl-4-[(N-Benzyl)carbamoyl]-3*S*-methyl-1-[3'*S*-*tert*-butoxycarbonylamino-3'-[(N-Benzyl)carbamoyl]prop-1'-yl]-2-oxoazetidine (63b). Syrup. Yield: 37% (from **52** and benzylamine). Eluent: Acetone:DCM (1:15). HPLC: t_R=14.40 min (gradient of 5% to 100% of A, in 20 min). [α_D] = +32.10. ^{1}H NMR (400 MHz, $CDCl_3$): δ 7.96 (br s, 1H, NHCO), 7.30-7.20 (m, 15H, Ar), 6.81 (brs, 1H, NHCO), 5.17 (d, 1H, *J*=6.6 Hz, 3'-NH), 4.58 (dd, 1H, *J*= 14.9, 6.1 Hz, NCH_2), 4.36 (m,

2H, NCH_2), 4.34 (dd, 1H, *J*=14.9, 6.1 Hz, NCH_2), 4.14 (m, 1H, 3'-H), 3.76 (d, 1H, *J*=14.7 Hz, 4-CH_2), 3.11 (m, 4H, 1'-H, 4-CH_2, 3-H), 1.84 (m, 2H, 2'-H), 1.40 (s, 9H, CH_3 ^{t}Bu), 1.10 (d, 3H, *J*=7.5 Hz, CH_3). ^{3}C NMR (75 MHz, $CDCl_3$): 171.9 (CON), 170.0 (COM, C2), 155.5 (OCON), 138.0, 136.0, 130.2, 129.9, 128.9, 128.8, 128.7, 128.4, 128.3, 127.8, 127.6, 127.4 (Ar), 78.8 (C ^{t}Bu), 69.7 (C4), 54.6 (C3), 52.7 (C3'), 44.0, 43.6 (NCH_2), 40.7 (4-CH_2), 40.1 (C1'), 32.1 (C2'), 28.4 (CH_3 ^{t}Bu), 10.2 (3-CH_3). MS $(ES)^+$: 599.31 $[M+H]^+$. Exact Mass calculated for$C_{35}H_{42}N_4O_5$: 598.31552; found: 598.31608.

4*R,S*-Benzyl-1-[4'*S*-tert-butoxycarbonylamino-4'-[(N-Benzyl)carbamoyl]but-1'-yl]-4-[(N-Benzyl)carbamoyl]-2-oxoazetidine (64). Syrup. Yield: 27% (from **53** and benzylamine). Eluent: EtOAc:Hexane (4:1). HPLC: t_R=13.80 min (gradient of 5% to 100% of A, in 20 min). Diastereoisomers ratio M,m = 60:40. ^{1}H NMR (400 MHz, $CDCl_3$, major diastereoisomer): δ 7.27-6.88 (m, 15H, Ar), 5.95 (m, 1H, CONH), 5.32 (m, 1H, CONH), 4.52-4.06 (m, 5H, NCH_2, 4'-NH), 3.58 (m, 1H, 4'-H), 3.51-2.86 (m, 6H, 1'-H, 3-H, 4-CH_2), 1.91-1.62 (m, 4H, 3'-H, 2'-H), 1.35 (s, 9H, CH_3 ^{t}Bu). ^{13}C NMR (75 MHz, $CDCl_3$): δ 172.3, 171.0 (CON), 167.2 (C2), 156.1 (OCON), 138.3, 137.3, 135.0, 129.9, 129.8, 128.9, 128.8, 128.7, 128.1, 128.0, 127.7, 127.5, (Ar), 80.0 (C ^{t}Bu), 63.8 (C4), 52.5 (C4'), 46.2 (C3), 43.9, 43.5 (NCH_2), 41.1 (C1'), 39.6 (4-CH_2), 30.9 (C3'), 28.5 (CH_3 ^{t}Bu), 24.6 (C2'). MS $(ES)^+$: 599.43 $[M+H]^+$.

4*R*-Benzyl-4-[N-[(pyridin-4''-yl)methyl]carbamoyl-1-[(3'*S*-*tert*-butoxycarbonylamino-3'-[N-[(pyridin-4''-yl)methyl]carbamoyl]prop-1'-yl]-2-oxoazetidine (65a). Syrup. Yield: 37% (from **51** and pyridin-4-yl-methylamine). Eluent: CH_2Cl_2:MeOH (9:1). HPLC: t_R=1.67 min (gradient of 5% to 100% of A, in 20 min). ^{1}H NMR (400 MHz, DMSO-d_6): δ 8.65 (t, 1H, *J*=5.9 Hz, CONH), 8.46 (t, 1H, *J*=5.9 Hz, CONH), 8.43 y 7.31-7.04 (m, 14H, Ar, 3'-NH), 4.36-4.22 (m, 4H, NCH_2,),

3.94 (m, 2H, 3'-H), 3.42 (d, 1H, *J*= 13.7 Hz, 4-CH_2), 3.30-3.18 (m, 2H, 1'-H), 3.13 (d, 1H, *J*= 13.7 Hz, 4-CH_2), 3.12 (d, 1H, *J*= 14.7 Hz, 4-CH_2), 2.89 (d, 1H, *J*= 14.7 Hz, 4-CH_2), 2.07 (m, 1H, 2'-H), 1.88 (m, 1H, 2'-H), 1.39 (s, 9H, CH_3 ^{t}Bu). ^{13}C NMR (75 Mz, DMSO-d_6): δ 173.1, 170.1 (CON), 167.0 (C2), 156.3 (OCON), 149.8, 149.7, 149.4, 148.9, 135.6, 130.5, 129.0, 127.8, 122.9, 122.6 (Ar), 79.5 (C ^{t}Bu), 63.9 (C4), 53.6 (C3), 45.1 (C3'), 42.2, 41.7 (NCH_2), 39.5 (C1', 4-CH_2), 30.9 (C2'), 28.7 (CH_3 ^{t}Bu). MS $(ES)^+$: 587.40 $[M+H]^+$.

4*S*-Benzyl-4-[N-[(pyridin-4''-yl)methyl]carbamoyl]-1-[3'*S*-*tert*-butoxycarbonylamino-3'-[N-[(pyridin-4''-yl)methyl]carbamoyl]prop-1'-yl]-2-oxoazetidine (65b). Syrup. Yield: 20% (from **51** and pyridin-4-yl-methylamine). Eluent: CH_2Cl_2:MeOH 10%. HPLC: t_R=1.67 min (gradient of 5% to 100% of A, in 20 min). ^{1}H NMR (400 MHz, $CDCl_3$): δ 8.46 and 7.30-6.97 (m, 13H, Ar), 7.76 (m, 1H, CONH), 7.10 (m, 1H, CONH), 5.43 (d, 2H, *J*=7.9 Hz, 3'-NH), 4.48-4.21 (m, 4H, NCH_2), 3.57-3.17 (m, 7H, 1'-H, 3'-H, 3-H, 4-CH_2), 1.50 (m, 2H, 2'-H), 1.41 (s, 9H, ^{t}Bu). ^{13}C NMR ($CDCl_3$): δ 172.2, 171.3 (CON), 167.5 (C2), 155.7 (OCON), 149.9, 149.8, 147.5, 146.9, 135.2, 129.8, 129.0, 127.7, 122.8, 122.4 (Ar), 80.4 (C ^{t}Bu), 64.2 (C4), 52.6 (C3), 45.6 (C3'), 42.7, 42.4 (NCH_2), 40.2 (4-CH_2), 39.5 (C1'), 32.4 (C3'), 28.4 (CH_3 ^{t}Bu). MS $(ES)^+$: 587.33 $[M+H]^+$.

4*R*,*S*-Benzyl-4-[(N-pyridin-3-yl)carbamoyl]-1-[(3'*S*-*tert*-butoxycarbonylamino-3'-[(N-pyridin-3-yl)carbamoyl]prop-1'-yl]-2-oxoazetidine (66). Syrup. Yield: 26% (from **51** and 3-aminopyridine). Eluent: CH_2Cl_2:MeOH 10%. Diastereoisomers ratio M,m=71:29. HPLC: t_R=3.27 min (gradient of 5% to 100% of A, in 20 min). ^{1}H NMR (400 MHz, $CDCl_3$, major diastereoisomer): δ 9.60 (s, 1H, CONH), 9.13 (s, 1H, CONH), 8.74 (d, 1H, *J*=2.5 Hz, 2-Py), 8.62 (d, 1H, *J*=2.7 Hz, 2-Py), 8.36 (dd, 1H, *J*=4.8, 1.5 Hz, Py), 8.32,7.29 (m, 5H, Py), 7.33-7.14 (m, 5H, Ph), 5.82 (d, 1H, *J*=7.6 Hz, 3'-NH), 4.68

(m, 1H, 3'-H), 3.58-3.18 (m, 4H, 4-CH_2, 1'-H), 3.10 (d, 1H, *J*=15.2 Hz, 3-H), 3.00 (d, 1H, *J*=15.0 Hz, 3-H), 2.39 (m, 1H, 2'-H), 1.99 (m, 1H, 2'-H), 1.17 (s, 9H, CH_3 ^{t}Bu). ^{13}C NMR (300 MHz, $CDCl_3$): δ 170.3, 169.0 (CON), 167.6 (C2), 156.5 (OCON), 146.4, 145.9, 144.7, 141.4, 134.7, 133.9, 130.6, 130.2, 129.9, 129.2, 127.9, 126.9, 123.8, 123.4 (Ar), 80.8 (C ^{t}Bu), 63.9 (C4), 52.6 (C3'), 47.2 (C3), 41.8 (C1'), 37.3 (4-CH_2), 33.8 (C2'), 28.13 (CH_3 ^{t}Bu). MS $(ES)^+$: 559.40 $[M+H]^+$.

4*R*-Benzyl-4-[(N-pyridin-4-yl)carbamoyl]-1-[3'*S*-tert-butoxycarbonylamino-3'-[(N-pyridin-4-yl)carbamoyl]prop-1'-yl]-2-oxoazetidine (67a). Syrup. Yield: 31% (from **51** and 4-aminopyridine). Eluent: CH_2Cl_2:MeOH 10%. HPLC: t_R=3.26 min (gradient of 5% to 100% of A, in 20 min). [α_D] = -33.32. ^{1}H NMR (400 MHz, DMSO-d_6): δ 10.41 (s, 1H, CONH), 10.20 (s, 1H, CONH), 8.44 (m, 8H, Py), 7.61-7.20 (m, 6H, Ph, 3'-NH), 4.10 (m, 1H, 3'-H), 3.42 (d, 1H, *J*=14.0 Hz, 4-CH_2), 3.32 (d, 1H, *J*=14.0 Hz, 4-CH_2), 3.22 (d, 1H, *J*=14.8 Hz, 3-H), 3.01 (m, 2H, 1'-H), 2.98 (d, 1H, *J*=14.8 Hz, 3-H), 2.24 (m, 1H, 2'-H), 2.06 (m, 1H, 2'-H), 1.39 (s, 9H, CH_3 ^{t}Bu). ^{13}C NMR (75 MHz, CD_3OD): δ 173.6, 172.7 (CON), 169.0 (C2), 157.9 (OCON), 150.8, 150.7, 147.8, 147.5, 135.6, 131.3, 129.7, 128.6, 115.7, 115.2 (Ar), 80.8 (C ^{t}Bu), 66.4 (C4), 55.1 (C3'), 47.3 (C1'), 45.4 (C3), 40.5 (4-CH_2), 32.1 (C2'), 28.7 (CH_3 ^{t}Bu). MS $(ES)^+$: 559.40 $[M+H]^+$.

4*S*-Benzyl-4-[(N-(pyridin-4-yl)carbamoyl]-1-[3'*S*-*tert*-butoxycarbonylamino-3'-[(N-pyridin-4-yl)carbamoyl]prop-1'-yl]-2-oxoazetidine (67b). Syrup. Yield: 23% (from **51** and 4-aminopyridine). Eluent: CH_2Cl_2:MeOH 10%. HPLC: t_R=3.26 min (gradient of 5% to 100% of A, in 20 min). [αD] = +54.82 ^{1}H NMR (400 MHz, DMSO-d_6): δ 10.42 (s, 1H, CONH), 10.26 (s, 1H, CONH), 8.49 (m, 4H, Py), 7.65 (m, 2H, Py), 7.62 (m, 2H, Py), 7.36-7.26 (m, 6H, Ph, 3'-NH), 4.18 (m, 1H, 3'-H), 3.47 (d, 1H, *J*=14.1 Hz, 4-CH_2), 3.37 (d, 1H, *J*=14.1 Hz, 4-CH_2), 3.27 (d, 1H, *J*=14.8 Hz, 3-H), 3.08 (m, 2H, 1'-H), 2.96 (d, 1H, *J*=14.8 Hz, 3-H), 2.15 (m, 2H, 2'-H), 1.45 (s, 9H, CH_3 ^{t}Bu). ^{13}C NMR (75 MHz,

CD_3OD): δ 173.7, 172.9 (CON), 168.7 (C2), 158.0 (OCON), 150.8, 150.7, 147.9, 147.5, 131.7, 131.2, 129.7, 128.6, 115.9, 115.2 (Ar), 80.9 (C ^{t}Bu), 66.2 (C4), 54.9 (C3'), 45.3 (C3), 40.7 (C3'), 40.5 (4-CH_2), 31.8 (C2'), 28.7 (CH_3 ^{t}Bu). MS $(ES)^+$: 559.40 $[M+H]^+$.

Table 1S. Amino acid-derived β–lactams prepared and their diastereomeric excess

Compd.	R[1]	R[2]	R[3]	R[4]	R[5]	R[6]	n	*de*[a] %
**39**	Ph	Me	Me	H	Boc	H	1	72
**40**	Ph	Me	Bn	H	Boc	H	1	60
**41**	Ph	Bn	Bn	H	Boc	H	1	6 (72)[b]
**42**	Ph	Me	tBu	H	Z	H	1	80
**43**	H	Me	Bn	H	Boc	H	1	82
**44**	H	Me	tBu	H	Z	H	1	38
**45**	Ph	Bn	Bn	H	Boc	Me	1	16
**46**	Ph	Bn	Bn	H	Boc	H	2	66
**47**	Ph	tBu	Me	H	Z	H	2	66
**48**	Ph	tBu	Me	Me	Z	H	2	42
**49**	Ph	tBu	Me	Me	H	H	2	46
**50**	H	tBu	Me	H	Z	H	2	34

[a] Measured by ^{1}H NMR. [b] Obtained after cyclization of the dibenzyl ester derivative **30**.

Table 2S. Chemical shifts of β-$CH_3(CH_2)$ protons and t_R in HPLC of dipeptide derivatives used for the configurational assignment

Compd.	Config. 4, 1'	R[4]	R[5]	R[6]	R[7]	XR[3]	n	δ ppm	t_R min
**56a**	*R,S*	H	Boc	H	Bn(*S*)	L-Phe-OMe	1	2.60 2.10	15.47
**56b**	*S,S*	H	Boc	H	Bn(*S*)	L-Phe-OMe	1	2.76 2.46	15.13
**57a**	*R,S*	H	Boc	H	Me(*S*)	L-Ala-OMe	1	1.37	12.65
**57b**	*S,S*	H	Boc	H	Me(*S*)	L-Ala-OMe	1	1.41	12.62
**58**	*R,S* (*S,S*)[a]	H	Z	H	Me(*S*)	OMe	2	1.25 (1.38)[a]	12.57 (12.21)[a]
**59**	*R,R* (*R,S*)[a]	H	Z	H	Me(*R*)	OMe	2	1.32 (1.31)[a]	12.17 (12.59)[a]
**60**	*R,S* (*S,S*)[a]	Me	Z	H	Me (*S*)	OMe	2	_[b]	9.51
**61**	*R,S* (*S,S*)[a]	Me	H	H	Me(*S*)	OMe	2	1.33 (1.41)[a]	7.95 (7.81)[a]

[a] Minor component in the mixture. [b] Complex spectrum due to rotamery around the BnOCON(Me)-bond

Table 3S. β–Lactam amide derivatives prepared

Compd.	Config. C4	R^6	R^2	n
**62**	*R,S*	H	Bn	1
**63a**	*R*	Me(R)	Bn	1
**63b**	*S*	Me(S)	Bn	1
**64**	*R,S*	H	Bn	2
**65**	*R,S*	H	CH_2(4-Py)	1
**66**	*R,S*	H	3-Py	1
**67a**	*R*	H	4-Py	1
**67b**	*S*	H	4-Py	1

Table 4S. Kinetic solubility for some selected compounds

Compound	1% DMSO		5% DMSO	
	μM	mg/L	μM	mg/L
**41**	<10	_	<10	_
**62a**	63	38	92	55
**65**	86	56	441	291
**66**	>100	_	>500	_

Table 5S. Blockade activity of compounds **41** and **45** on different ion channels (Patch-Clamp)

Ion channel	Compd **41** (IC_{50} nM)	Compd **45** (IC_{50} nM)
TRPM8	46.0±5.6	82.9±11.4
hTRPV1	>1000	>1000
TRPA1	>1000	>1000
Kv1.1	>1000	>1000
NaV1.6	>1000	>1000

Table 6S. Computational alanine scanning and theoretical binding energy for the interaction of compound **41** at the two binding sites of TRPM8 channel.

**SITE 1**		
**Protein Residue**	**E(kcal/mol)**	**ΔE**
WT	141.3	0.0
Y754A	131.9	-9.4
L697A	135.8	-5.5
F700A	138.1	-3.2
I701A	137.5	-3.8
L704A	141.2	-0.1
L750A	141.4	0.1
L751A	138.4	-2.9
N799A	142.8	1.5
T803A	138.5	-2.8
T840A	141.9	0.6
L843A	137.8	-3.5
L1001A	141.1	-0.2
E1004A	129.3	-12.0
Y1005A	141.5	0.2
R1008A	146.2	4.9
L1009A	140.8	-0.5

**SITE 2**		
**Protein Residue**	**E(kcal/mol)**	**ΔE**
WT	94.8	0.0
W798A	85.7	-9.1
D802A	91.3	-3.5
Y836A	88.2	-6.6
L864A	91.8	-3.0
I865A	94.5	-0.3
F868A	90.8	-4.0
F869A	89.2	-5.6
F872A	91.1	-3.7
L873A	94.0	-0.8
(D) I962A*	93.1	-1.7
(D) L965A*	94.4	-0.4

Table 7S. Residues involved in binding of β–lactam derivative **41** at the predicted binding sites of TRPM8 channel. The equivalent residues in the other TRP channels were deduced form the structural alignment and the position of the compound.

Site	TRPM8	TRPV1	TRPA1	TRPV2	TRPV6
	Y754	R491	K787	G451	V401
**1**	L697	N437	N722	N396	L331
	E1004	D707	L986	E670	M602
	W798	Y511	L807	Y471	G422
**2**	Y836	M547	F841	L507	L459
	F869	C578	L871	L538	M490

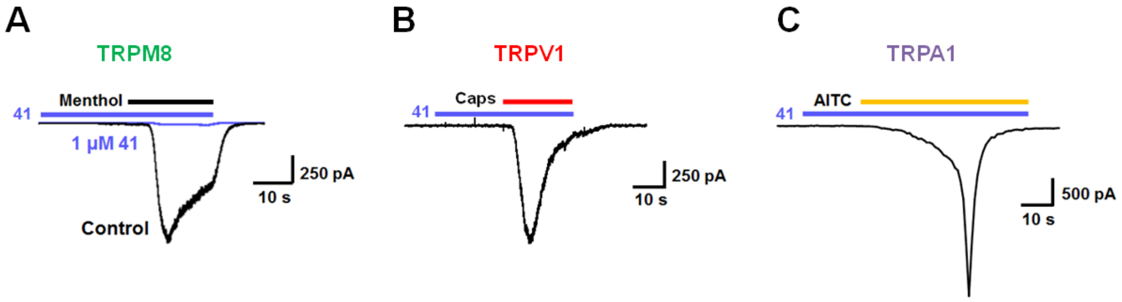

Figure 1S. Compound 41 did not produce activation on TRPM8, TRPV1 and TRPA1. A) Representative menthol-evoked TRPM8 ionic currents in absence (black line) or presence of 1µM compound **41** (blue line). Pre-application of compound (20 s) was followed by coapplication with 500 µM menthol for 20s. **B**) Representative capsaicin-evoked TRPV1 current in presence of 1µM compound **41**. Pre-application of compound (20 s) was followed by coapplication with 1 µM capsaicin for 20s. **C**) Representative AITC-evoked TRPA1 current in presence of 1µM compound **41**. Pre-application of compound (20 s) was followed by coapplication with 100 µM AITC for 60s. Voltage was held at -60 mV for all the experiments.

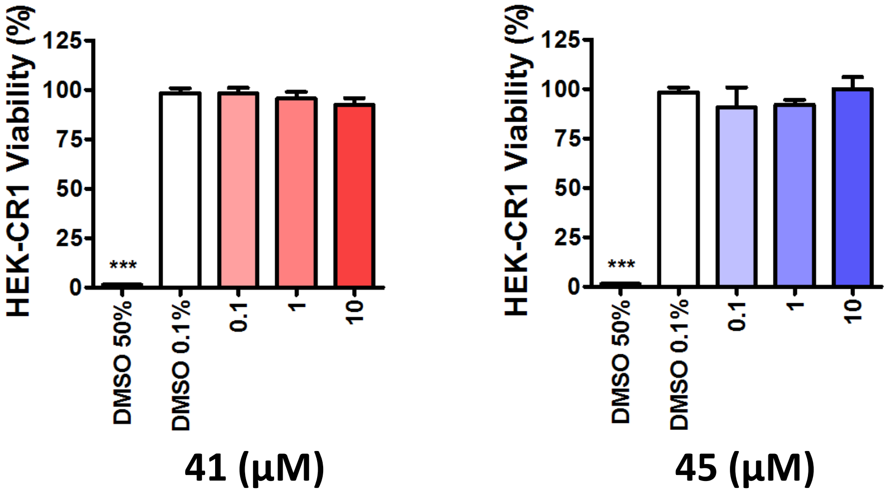

Figure 2S. Effect of derivative 41 and 45 on MTT-based assay of cytotoxicity in HEK-CR1 cells. A) Cell survival after 24 hours incubation at three concentrations of derivative **41** or **B)** derivative **45**. Each value is expressed as mean ± SEM. n ≥ 4, ANOVA with Bonferroni post hoc test (*** $p < 0.001$).

Cell viability assay (MTT)

Cell viability was assessed by the detection of mitochondrial activity in living cells using a modified colorimetric analysis of Blue Tetrazolium Bromide Thiazolyl (MTT). Briefly, HEK293 cells (2×10^4 cells/well) were subcultured in 96-well plates, grown until 80–90% confluence, and incubated with increasing concentrations of testing compounds for 24 h. Following treatment, 10 µL of MTT solution (5 mg/mL in phosphate buffered saline) was added to each well and further incubated for 4 h at 37 °C. Subsequently, 100 µL of DMSO was added to each well to dissolve any deposited formazan resulting from cleavage and reduction of MTT by active mitochondrial dehydrogenases. The optical density of each well was measured at 540 nm with a microplate reader (Polastar BMG LABTECH, Offenberg, Germany).

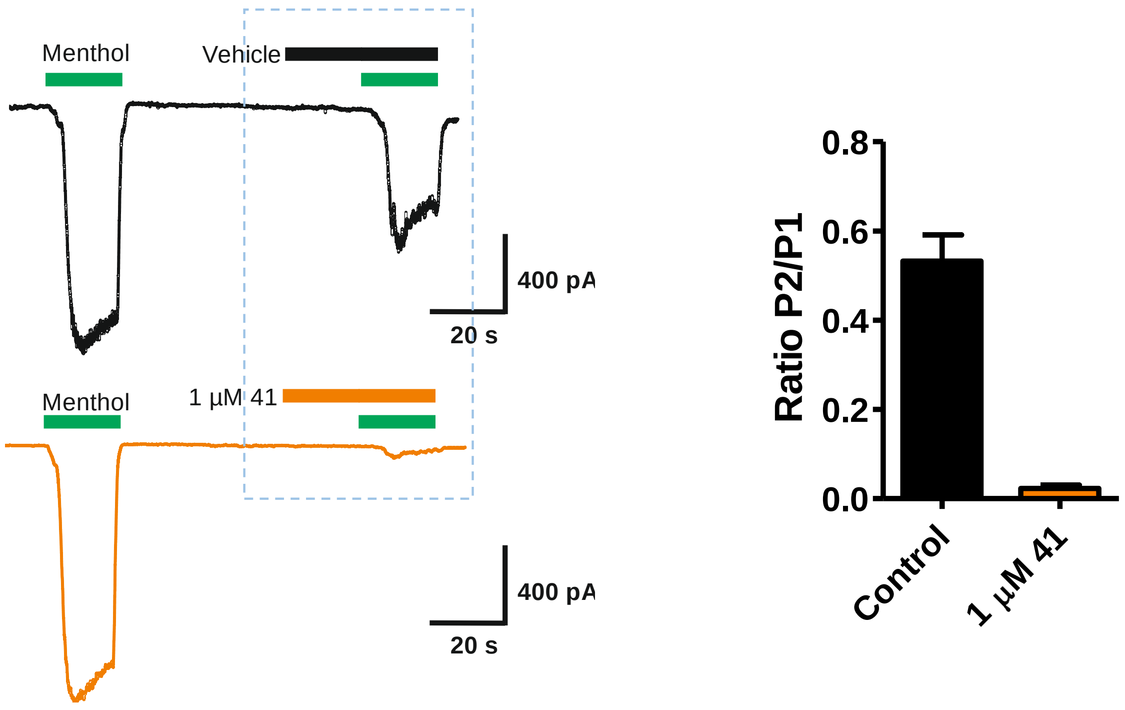

Figure 3S. Channel desensitization. A) Representative menthol-evoked TRPM8 ionic currents in two consecutive pulses, in the absence (black line) or presence of 1µM compound **41** (orange line). C) Ratio currents between pulse 2 and pulse 1 induced by menthol in the absence (Black bar or in the presence (orange bar) of compound **41**.

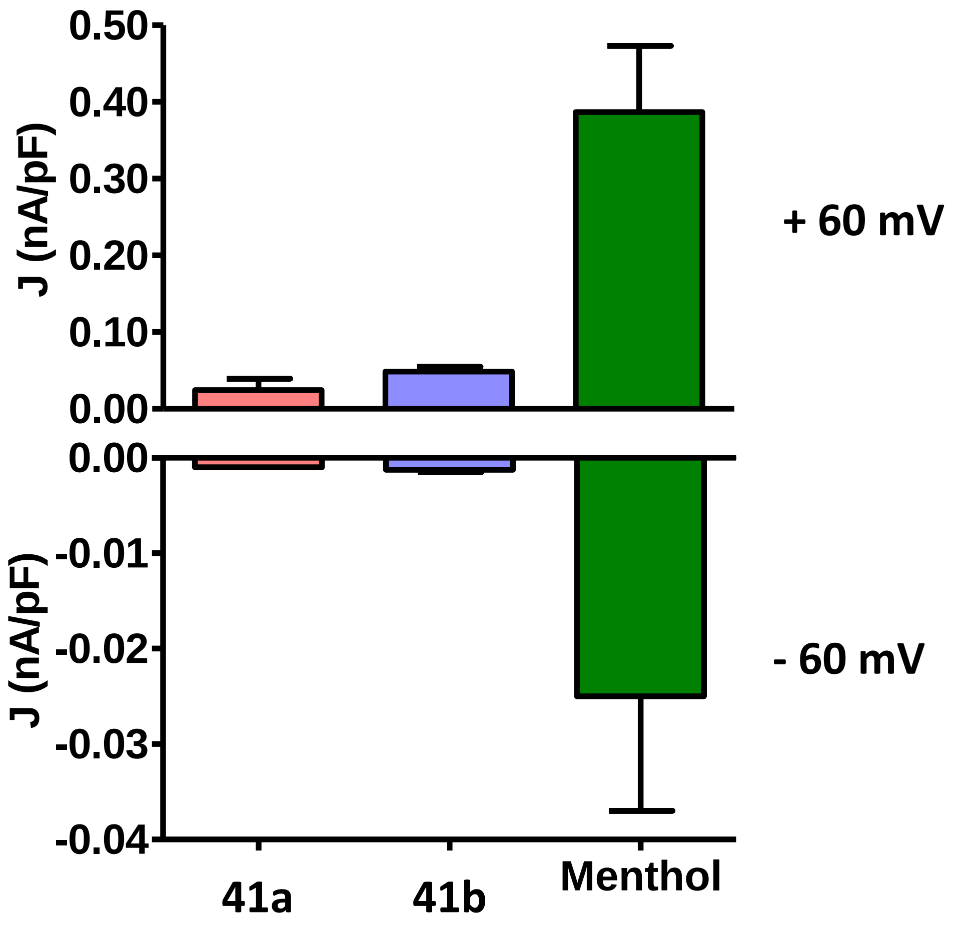

Figure 4S. Activity of separated diastereoisomers of **41** (compounds added at a 1 µM concentration)

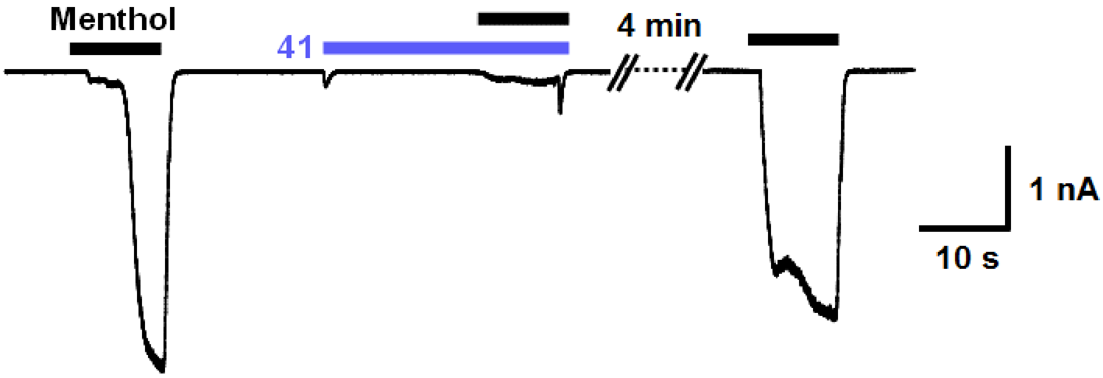

Figure 5S. Compound 41 blocked menthol-evoked response on TRPM8 in a reversible manner. Representative menthol-evoked TRPM8 ionic currents (black line) in absence or presence of 1µM compound **41** (blue line). A first pulse (10 s) of 500 µM was applied to establish the maximal menthol-evoked TRPM8 current. Pre-application of compounds (20 s) was followed by coapplication with 500 µM menthol for 10s. Four minutes after compound **41** application, a pulse of menthol was applied to measure the TRPM8 ionic current recovery. Holding potential -60 mV. Extracellular Ca^{2+} was removed to avoid TRPM8 desensitization.

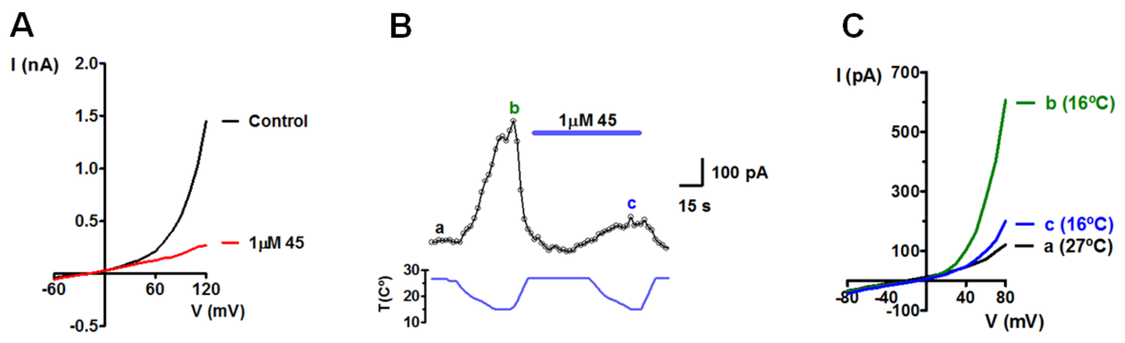

Figure 6S. **Compound 45 abolished voltage and the cold thermal activity response on TRPM8.** A) I-V relationships of TRPM8 channel activity in the absence (black) and presence of 1 µM **45** (red). B) Representative families of TRPM8 ionic currents at +80 mV in the presence of 1 µM **45** activated by cold temperature (16 ºC) obtained with a 300-ms ramp from -80 to +80 mV in intervals of 2 s during 3 minutes. Holding potential -60 mV. C) I-V relationship of TRPM8 channel in basal conditions (a, black line), activated by cold in the absence (b, green line) and presence of 1 µM **45** (c, blue line).

Molecular modelling. Experimental

Docking: The protein-ligand docking and the analysis of interactions were accomplished with Autodock,[7] implemented in the general purpose molecular modelling software Yasara,[8],[9] and optimized with AMBER 03 force field.[10] Docking trials were optimized and clustered to remove redundancy and sorted by binding energy.

The non-covalent interactions in protein-ligand complexes from the options obtained with the docking were studied with the web service Protein-Ligand Interaction Profiler (PLIP, https://projects.biotec.tu-dresden.de/plip-web/plip/index).

Binding energy: The binding energy of the complexes was recalculated with the "BindEnergy" command implemented in Yasara program. It calculates the binding energy of the selected ligand with respect to the rest of the complex, according to the force field called "yasara",[11] implemented in Yasara Structure distribution, having knowledge based components for increased accuracy in protein simulations. The force field allowed the evaluation of the properties of the complex interactions, where parameters such as atomic contact map, accessibility of the atoms and residues, backbone dihedral angles, hydrogen bonds and electrostatic networks of the protein were assessed. The binding energy is obtained by calculating the energy at infinite distance (between the selected ligand and receptor, i.e. the unbound state)[12] and subtracting the energy of the complex (i.e. the bound state). The more positive the binding energy, the more favourable is the interaction in the context of the chosen force field. In this context, Yasara reports positive 'binding energies', that is, the energy required to disassemble a complex into separate parts, usually positive. Yasara "pH" command was set to 7.0, ensuring that molecules preserve their pH dependency of bond orders and protonation patterns.

Energy minimization: After docking or mutagenesis, a local energy minimization is performed with "Experiment Minimization" command in Yasara. To remove possible bumps and correct the covalent geometry, the structure was energy-minimized with the "yasara" force field, using a 7.9 Å force cut-off to treat long range electrostatic interactions. After removal of conformational stress by a short steepest descent minimization, the procedure continued by simulated annealing (time step 2 fs, atom velocities scaled down by 0.9 every 10th step) until convergence was reached, i.e. the energy improved by less than 0.01 kcal/mol per atom during 200 steps.

---

[7] Morris, G.M.; Huey. R.; Lindstrom, W.; Sanner, M. F.; , R. K.; Goodsell, D. S.; Olson A. J. AutoDock4 and AutoDockTools4: Automated docking with selective receptor flexibility. *J. Comput. Chem*. **2009**, *30*, 2785-2791.

[8] Krieger, E.; Koraimann, G.; Vriend, G. Increasing the precision of comparative models with YASARA NOVA--a self-parameterizing force field. *Proteins* **2002,** *47*, 393-402.

[9] Krieger, E.; Darden, T.; Nabuurs, S. B.; Finkelstein, A.; Vriend, G. Making optimal use of empirical energy functions: force-field parameterization in crystal space. *Proteins* **2004,** *57*, 678-683.

[10] Duan, Y.; Wu, C.; Chowdhury, S.; Lee, M. C.; Xiong, G.; Zhang, W.; Yang, R.; Cieplak, P.; Luo, R.; Lee, T. A point-charge force field for molecular mechanics simulations of proteins based on condensed-phase quantum mechanical calculations. *J. Comput. Chem.* **2003**, *24*, 1999-2012.

[11] Krieger, E.; Joo, K.; Lee, J.; Lee, J.; Raman, S.; Thompson, J.; Tyka, M.; Baker, D.; Karplus, K. Improving physical realism, stereochemistry, and side-chain accuracy in homology modeling: Four approaches that performed well in CASP8. *Proteins: Struct., Funct., Bioinf.* **2009,** 77, 114-122.

[12] Teng, J.; Loukin, S.H.; Anishkin, A.; Kung, C. A competing hydrophobic tug on L596 to the membrane core unlatches S4-S5 linker elbow from TRP helix and allows TRPV4 channel to open. *Proc Natl Acad Sci U S A* **2016**, *113*, 11847-11852.

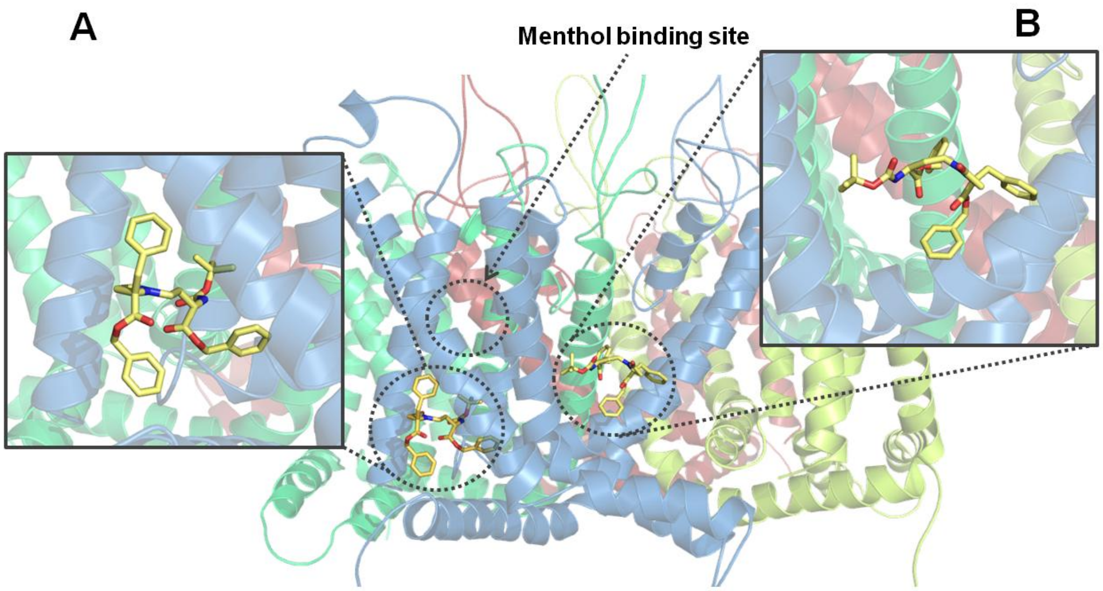

Figure 7S. Energetically most favourable binding sites for the interaction of compound **41** with the TRPM8 channel: site 1 (A), and site 2 (B). The location of the menthol binding site is also indicated.

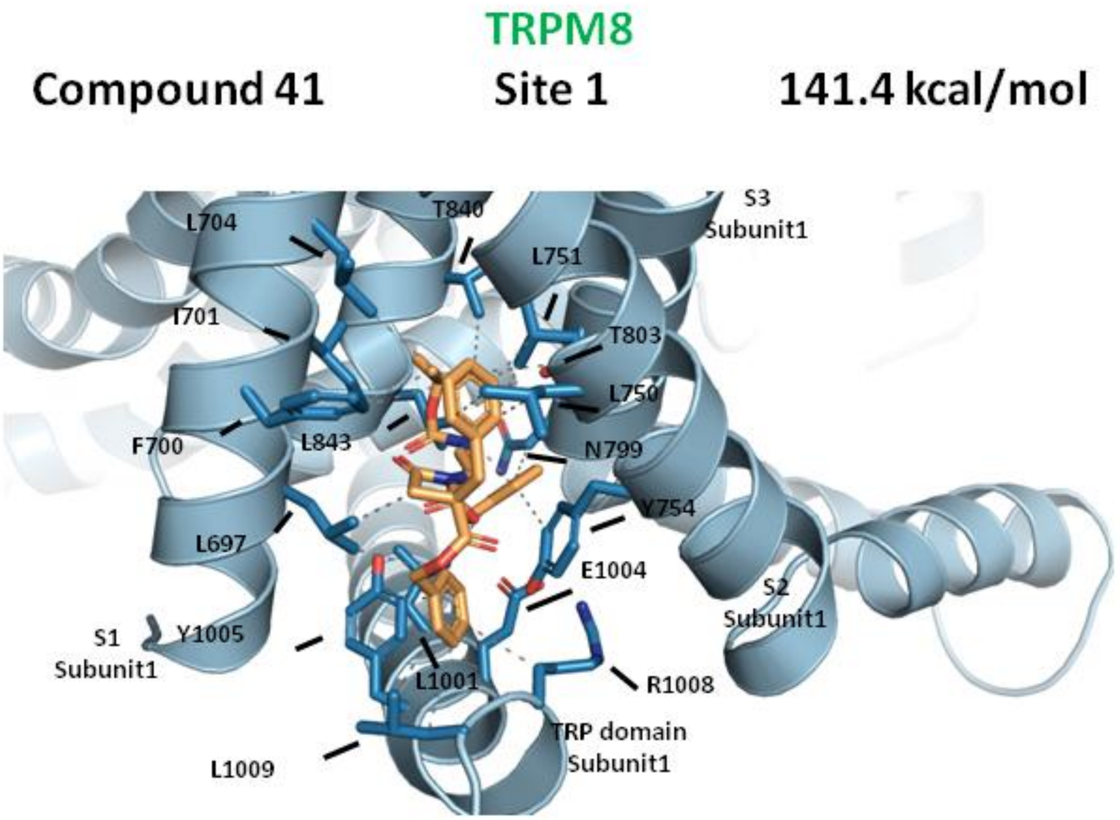

Located interactions among **41** and TRPM8 channel (Site 1)

**41**	Group	Protein residue subunit 1(domain)																Type of interaction[a]
		L697 (S1)	F700 (S1)	I701 (S1)	L704 (S1)	L750 (S2)	L751 (S2)	Y754 (S2)	N799 (S3)	T803 (S3)	T840 (S4)	L843 (S4)	L1001 (TRP)	E1004 (TRP)	Y1005 (TRP)	R1008 (TRP)	L1009 (TRP)	
C3	CH_2	X																VdW
R^1	Ph		X		X	X	X											VdW
R^2	Ph	X												X	X	X	X	VdW
R^3	Ph							X	X			X	X					VdW
R^5	^{t}Bu			X						X	X							VdW

[a] VdW: Van der Waals.

Figure 8S. Details of the interaction of compound **41** with TRPM8 channel at Site 1

TRPM8

Compound 41 Site 2 94.3 kcal/mol

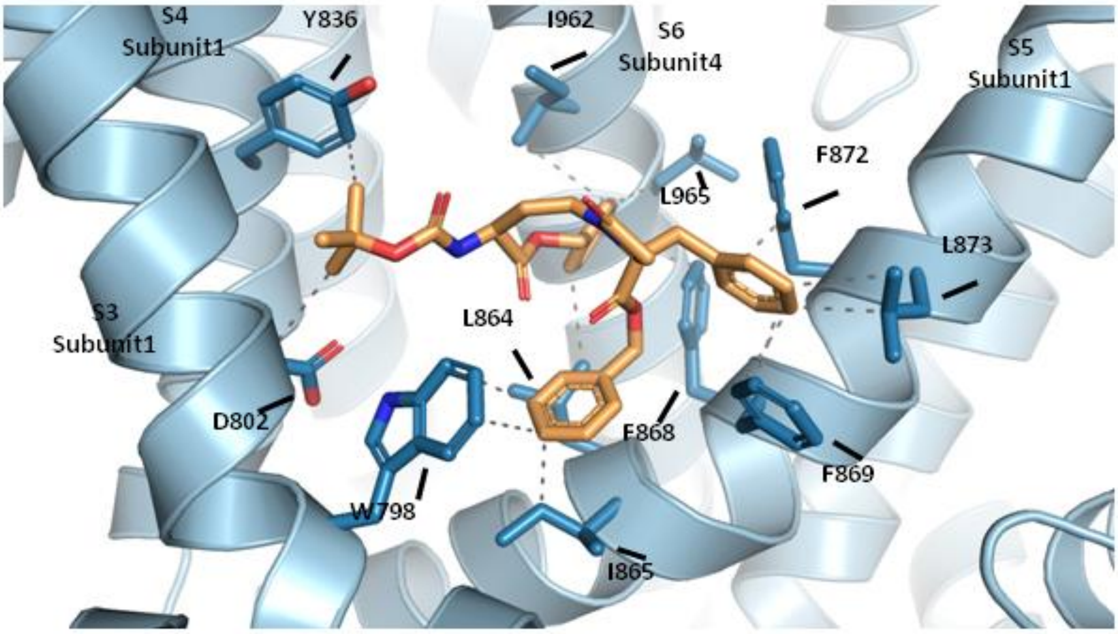

Located interactions among **41** and TRPM8 channel (Site 2)

**41**	Group	Protein residue (domain, subunit)											Type of interaction[a]
		W798 (S3,1)	D802 (S3,1)	Y836 (S4,1)	L864 (S5,1)	I865 (S5,1)	F868 (S5,1)	F869 (S5,1)	F872 (S5,1)	L873 (S5,1)	I962 (S6,4)	L965 (S6,4)	
R^1	Ph							X	X	X			VdW
R^2	Ph	X			X	X							VdW
R^3	Ph				X		X				X	X	VdW
R^5	^{t}Bu		X	X									VdW

[a] VdW: Van der Waals.

Figure 9S. Details of the interaction of compound **41** with TRPM8 channel at Site 2

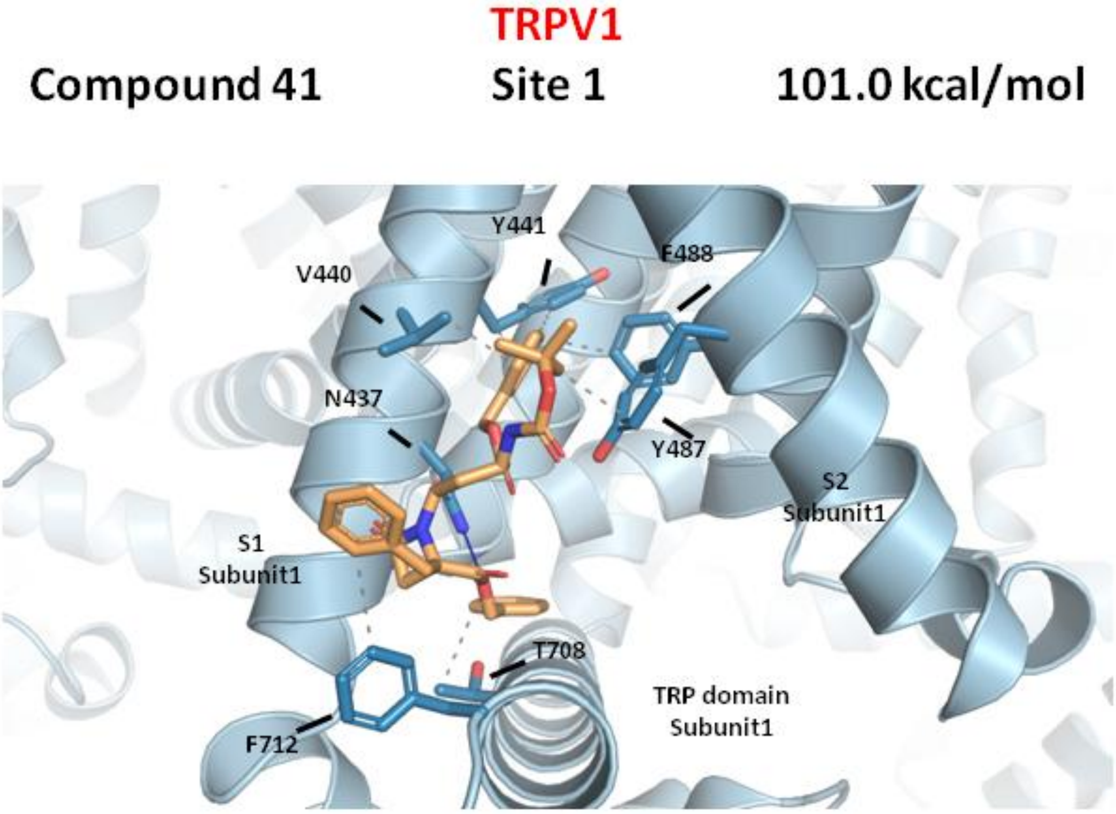

Located interactions among **41** and TRPV1 channel (Site 1)

Subst. of 41	Group implicated	Protein residue subunit 1 (domain)							Type of interaction[a]
		N57 (S1)	V60 (S1)	Y61 (S1)	Y107 (S2)	F108 (S2)	T305 (TRP)	F309 (TRP)	
R^1	Ph							X	VdW
R^2	Ph						X		VdW
	O (ester)	X (NH_2)							HB
R^3	Ph		X	X		X			VdW
R^5	^{t}Bu				X				VdW

[a] VdW: Van der Waals. HB: hydrogen bond

Figure 10S. Details of the interaction of compound **41** with TRPV1 channel at Site 1

TRPV1

Compound 41 Site 2 84.1 kcal/mol

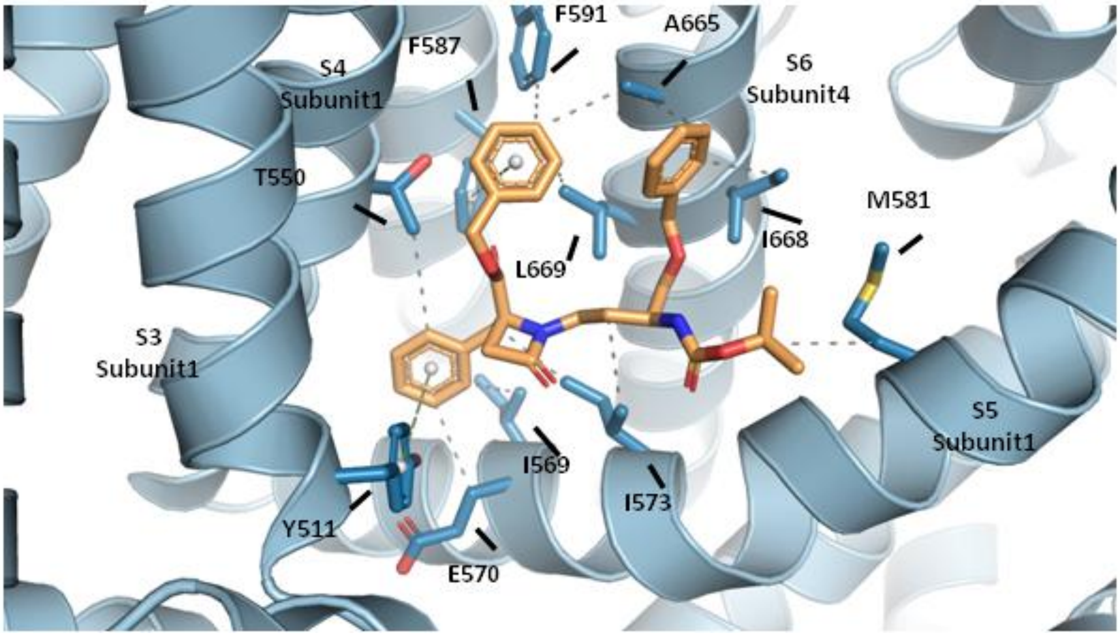

Located interactions among **41** and TRPV1 channel (Site 2)

**41**	Group	Protein residue (domain, subunit)											Type of interaction[a]
		Y131 (S3,1)	T170 (S4,1)	I189 (S5,1)	D190 (S5,1)	I193 (S5,1)	M201 (S5,1)	F207 (S5,4)	F211 (S5,4)	A262 (S6,4)	I265 (S6,4)	L266 (S6,4)	
2'-CH_2						X							VdW
R^1	Ph		X	X	X								VdW
R^1	Ph	X											Ar-Ar
R^1	CH_2					X							VdW
R^2	Ph								X	X		X	VdW
R^2	Ph							X					Ar-Ar
R^3	Ph									X	X		VdW
R^5	^{t}Bu						X						VdW

[a] VdW: Van der Waals. Ar-Ar: π-π stacking

Figure 11S. Details of the interaction of compound **41** with TRPV1 channel at Site 2 .

^{1}H-NMR (400 MHz, $CDCl_3$) and ^{13}C-NMR (100 MHz, $CDCl_3$) (39)

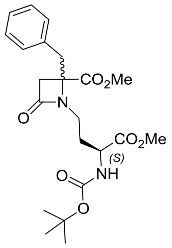

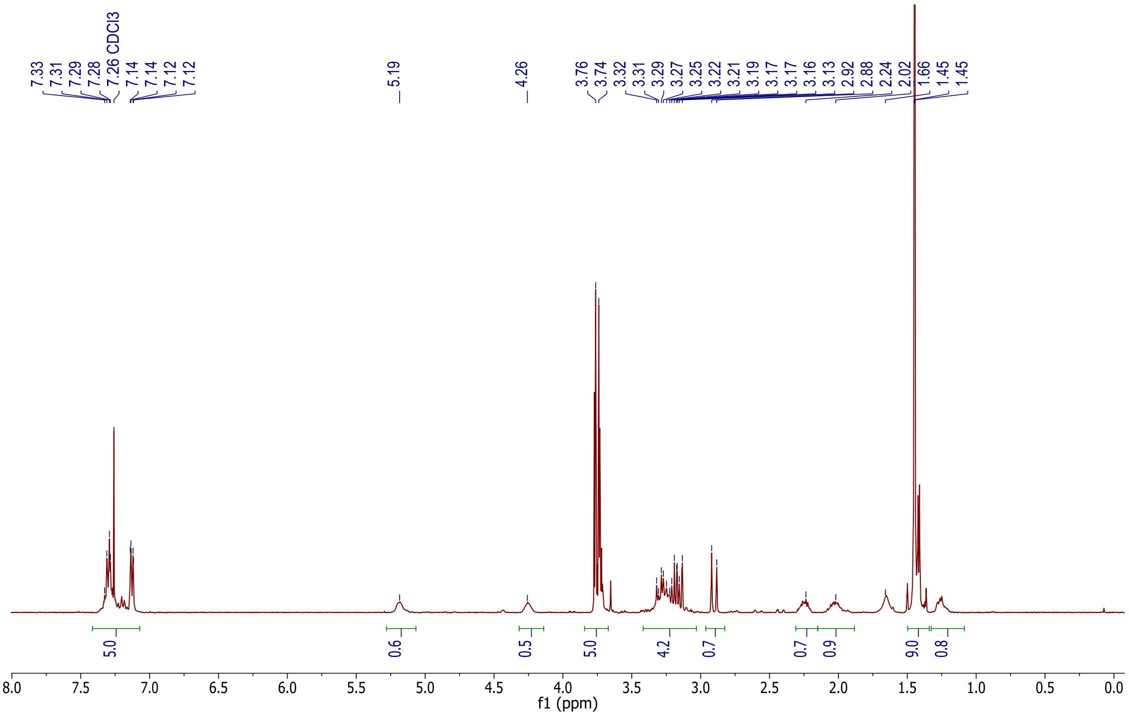

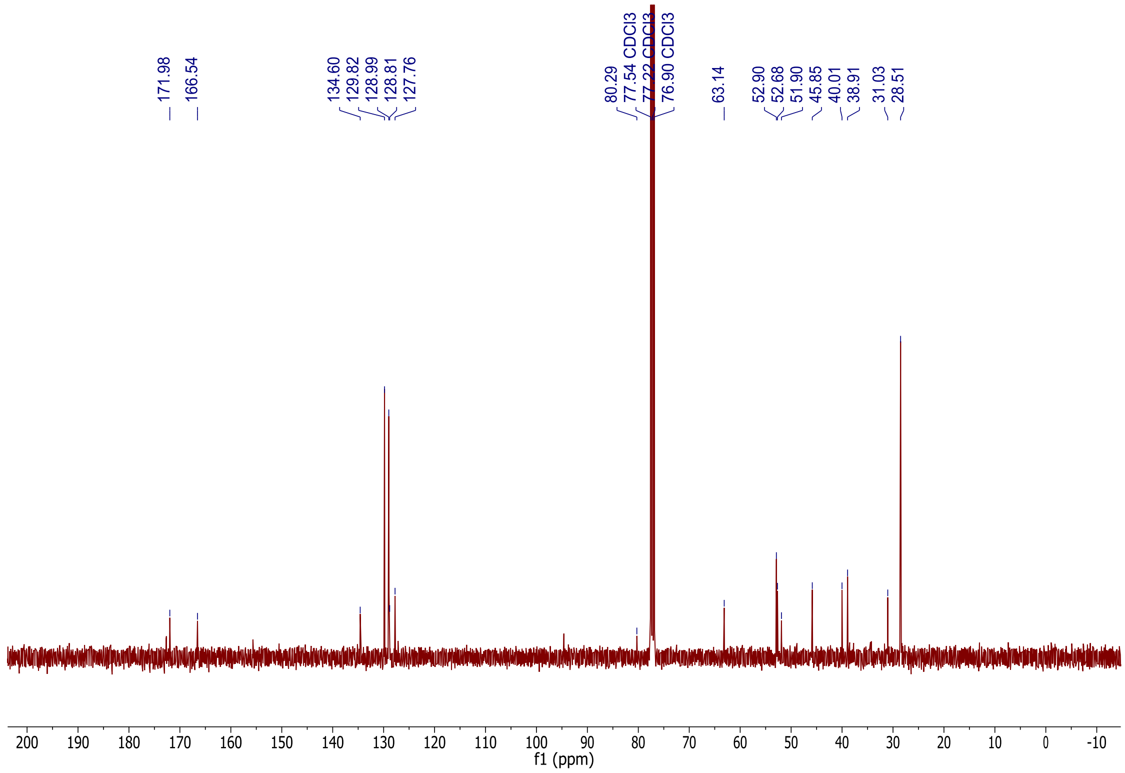

^{1}H-NMR (400 MHz, CDCl$_3$) and ^{13}C-NMR (100 MHz, CDCl$_3$) (40)

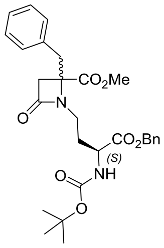

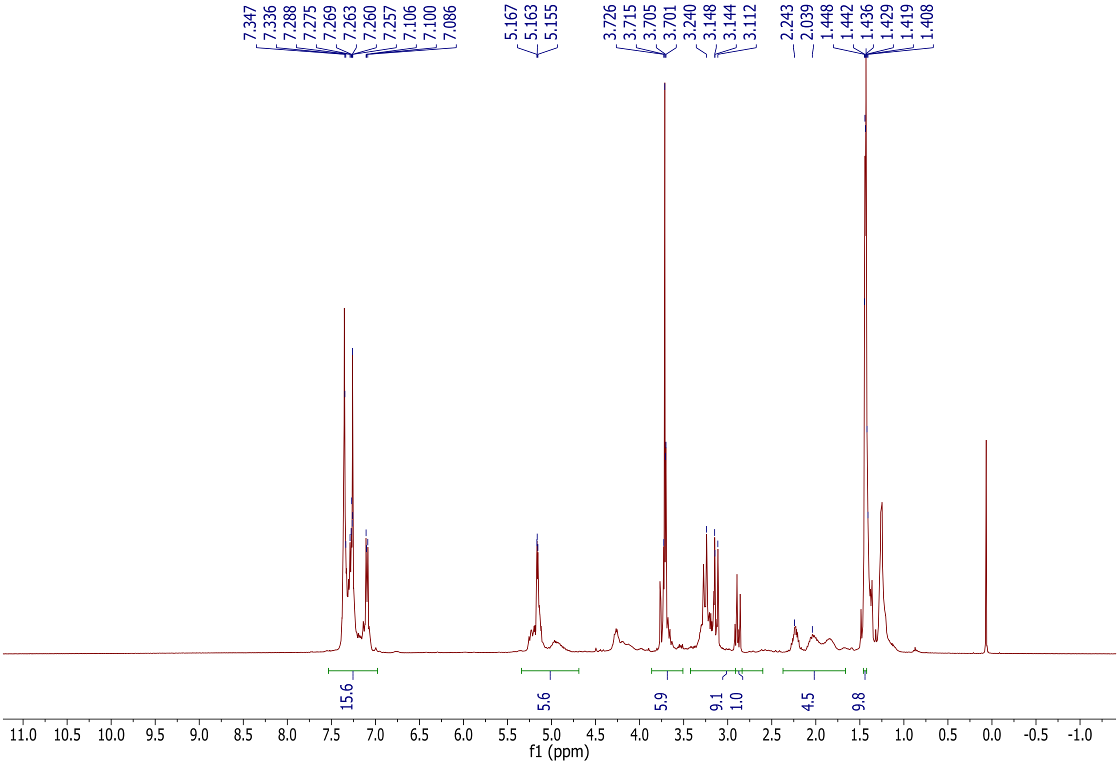

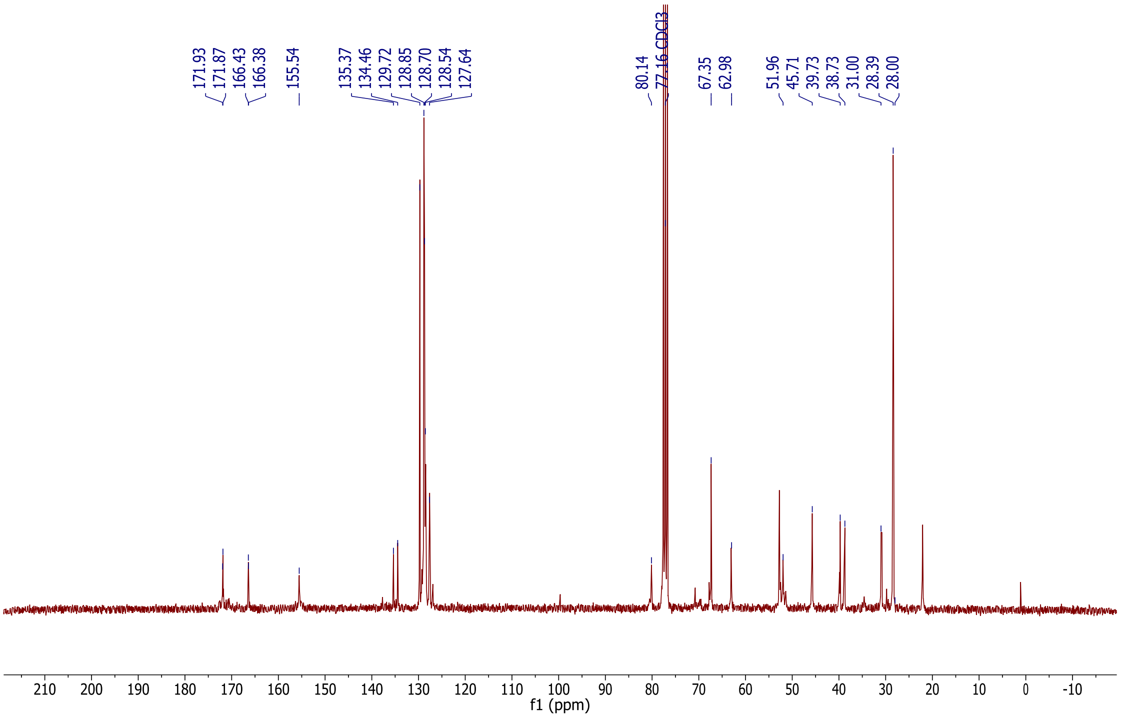

^{1}H-NMR (400 MHz, $CDCl_3$) and ^{13}C-NMR (75 MHz, $CDCl_3$) (41)

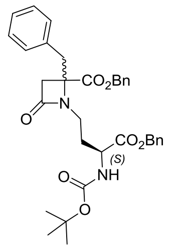

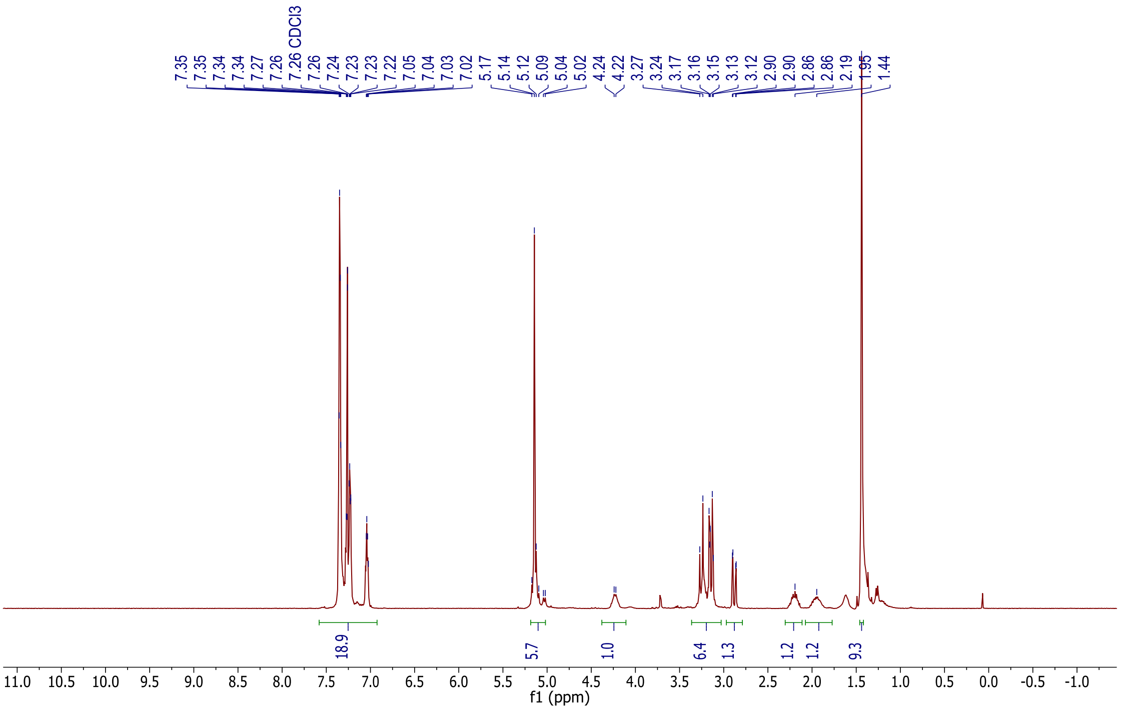

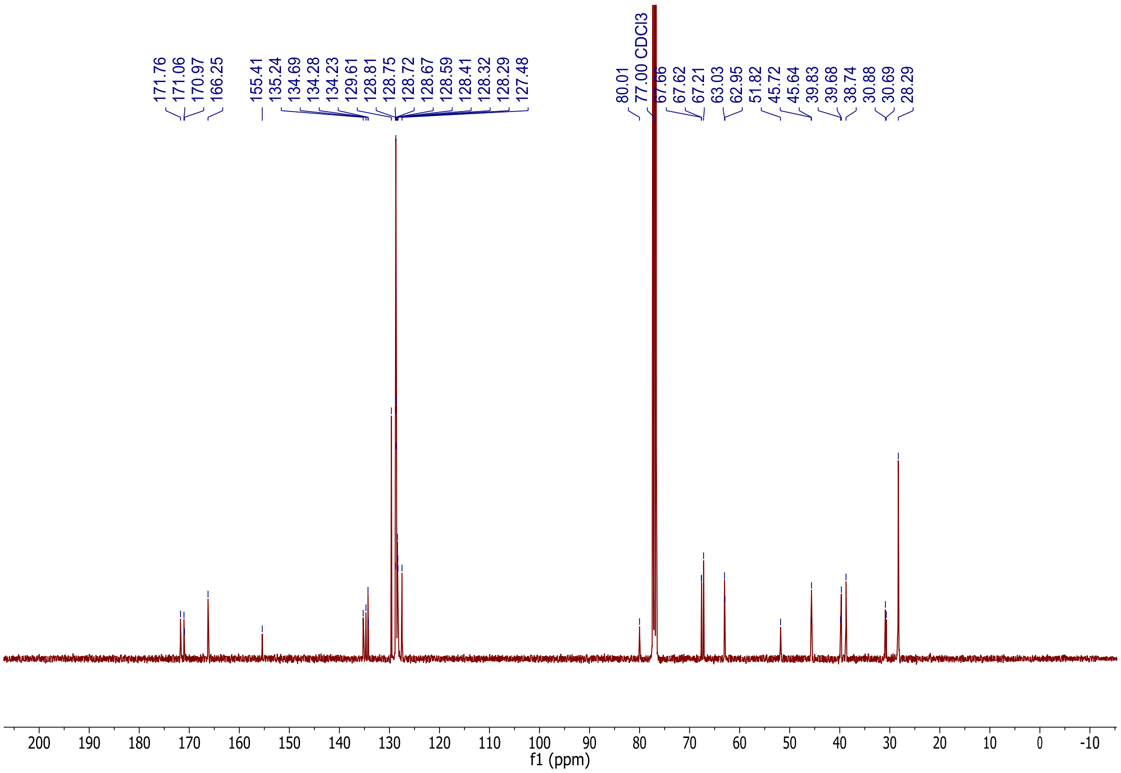

^{1}H-NMR (400 MHz, $CDCl_3$) and ^{13}C-NMR (75 MHz, $CDCl_3$) (41a)

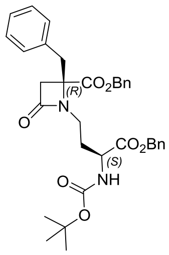

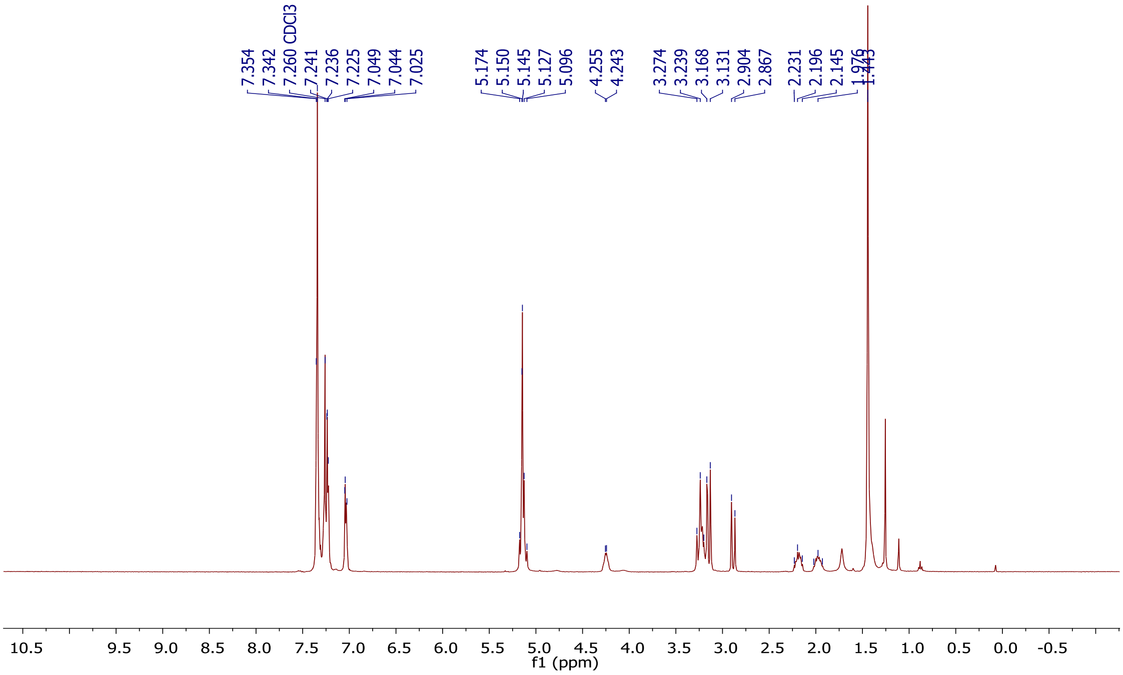

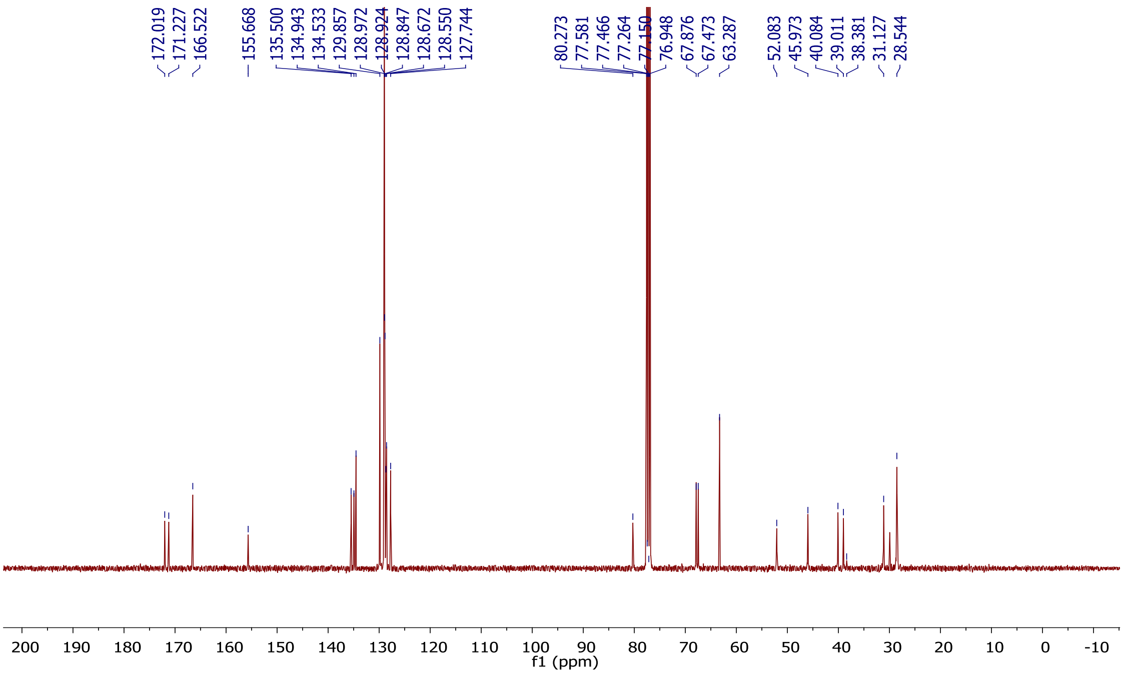

^{1}H-NMR (400 MHz, $CDCl_3$) and ^{13}C-NMR (75 MHz, $CDCl_3$) (41b)

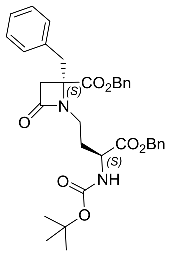

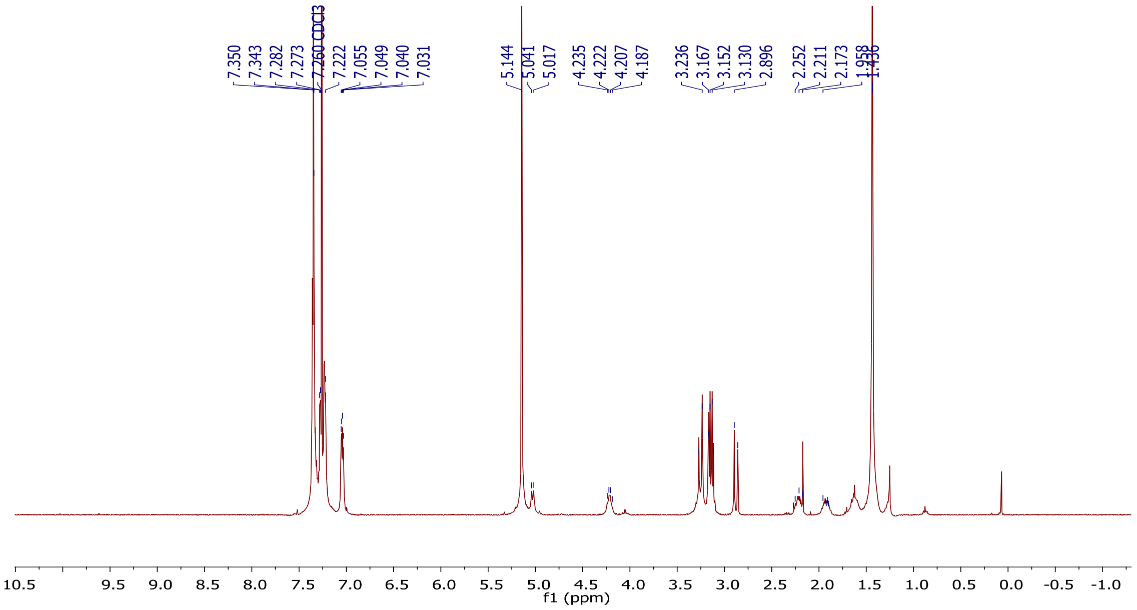

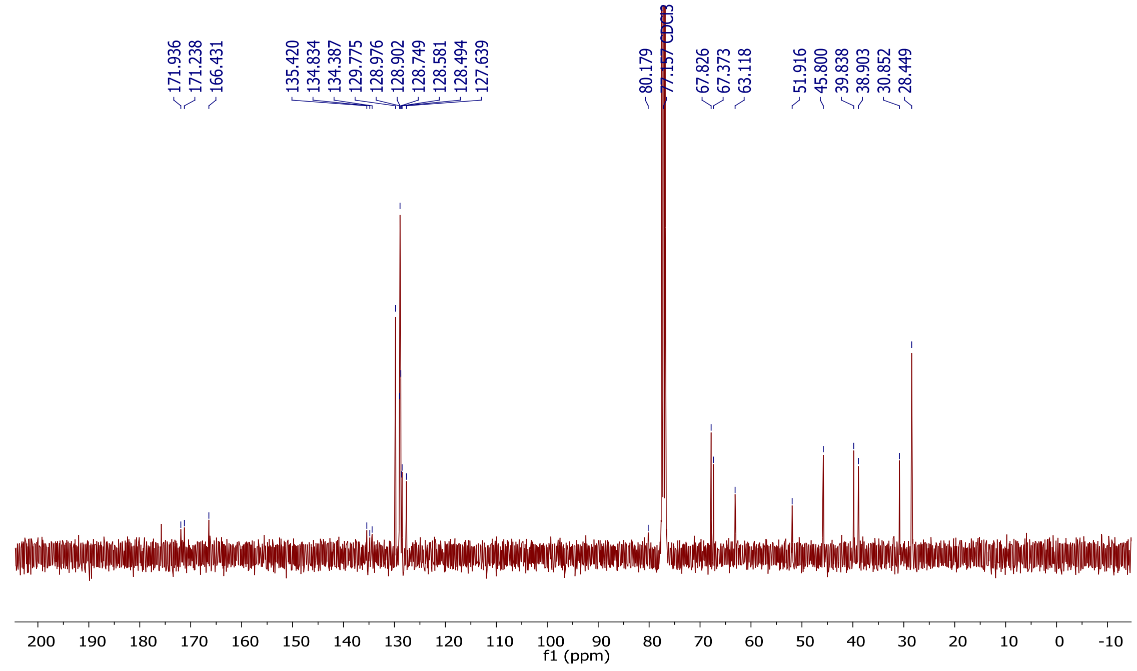

^{1}H-NMR (400 MHz, CDCl$_3$) and ^{13}C-NMR (75 MHz, CDCl$_3$) (42)

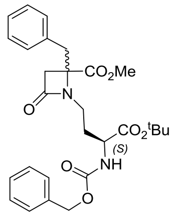

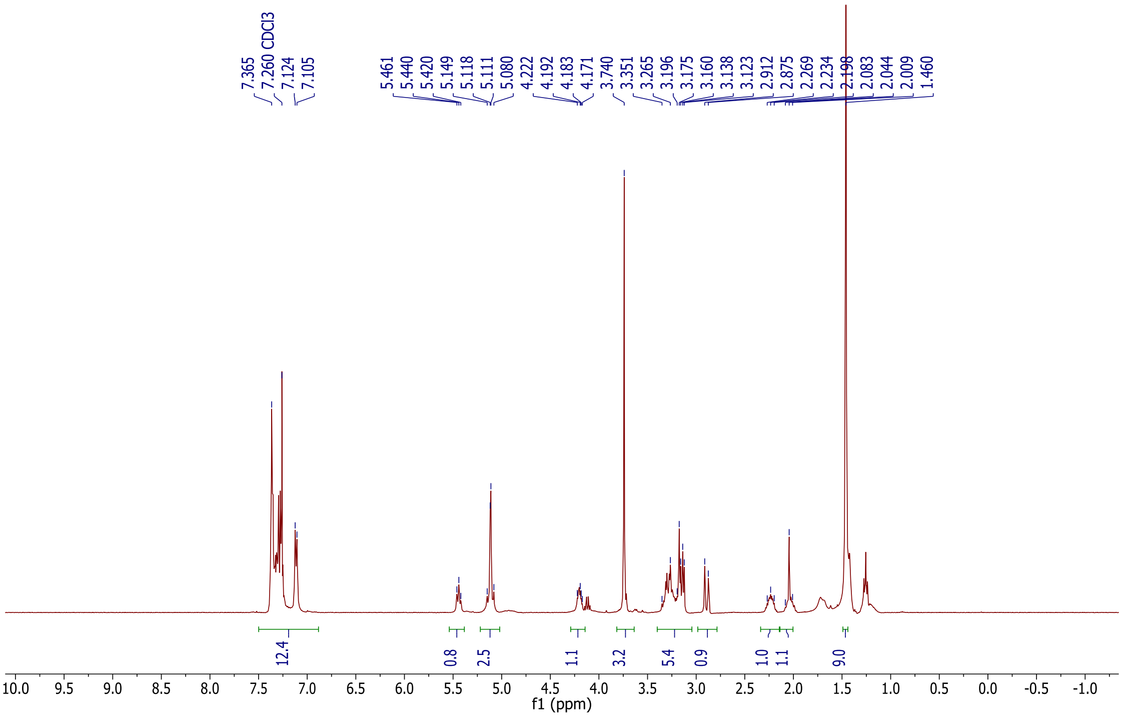

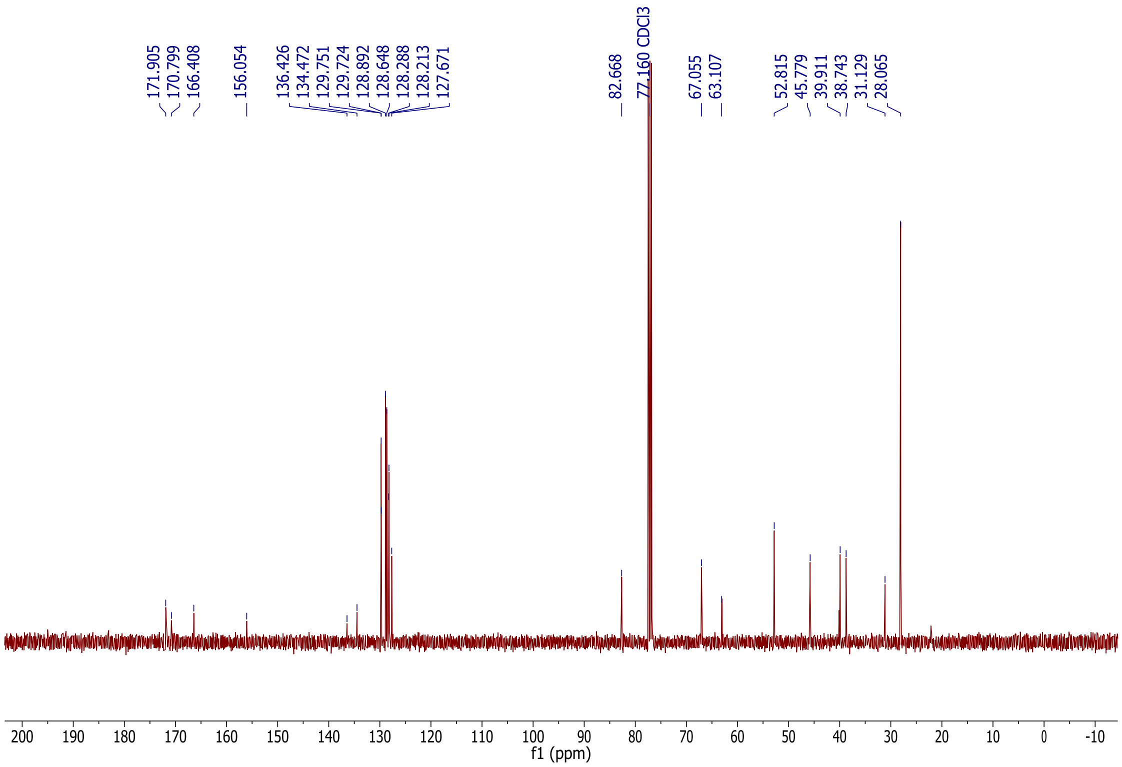

^{1}H-NMR (400 MHz, $CDCl_3$) and ^{13}C-NMR (75 MHz, $CDCl_3$) (43)

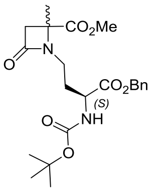

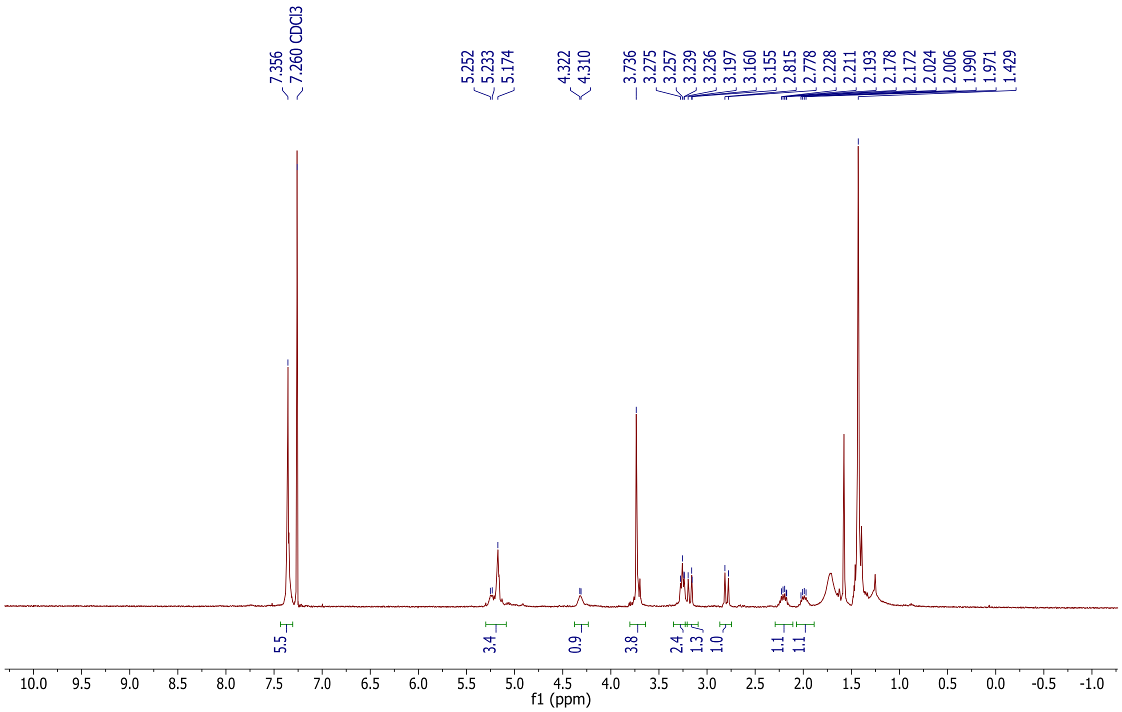

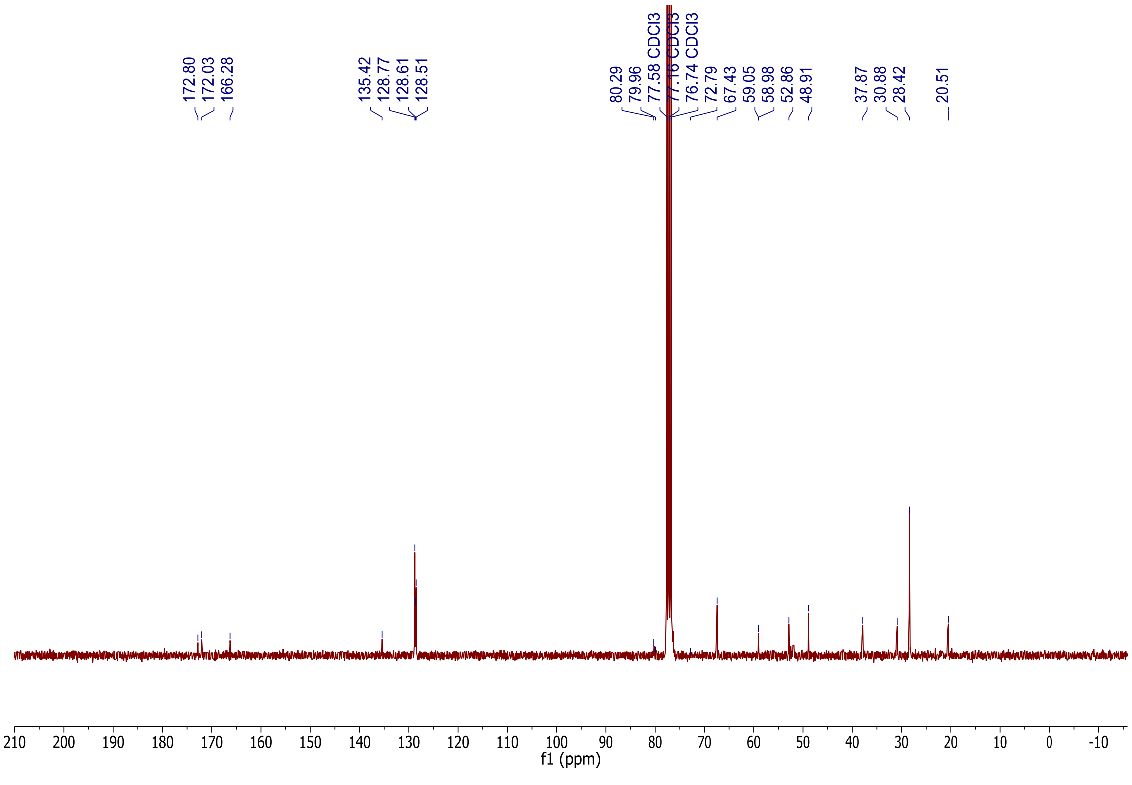

^{1}H-NMR (400 MHz, $CDCl_3$) and ^{13}C-NMR (75 MHz, $CDCl_3$) (44)

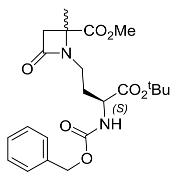

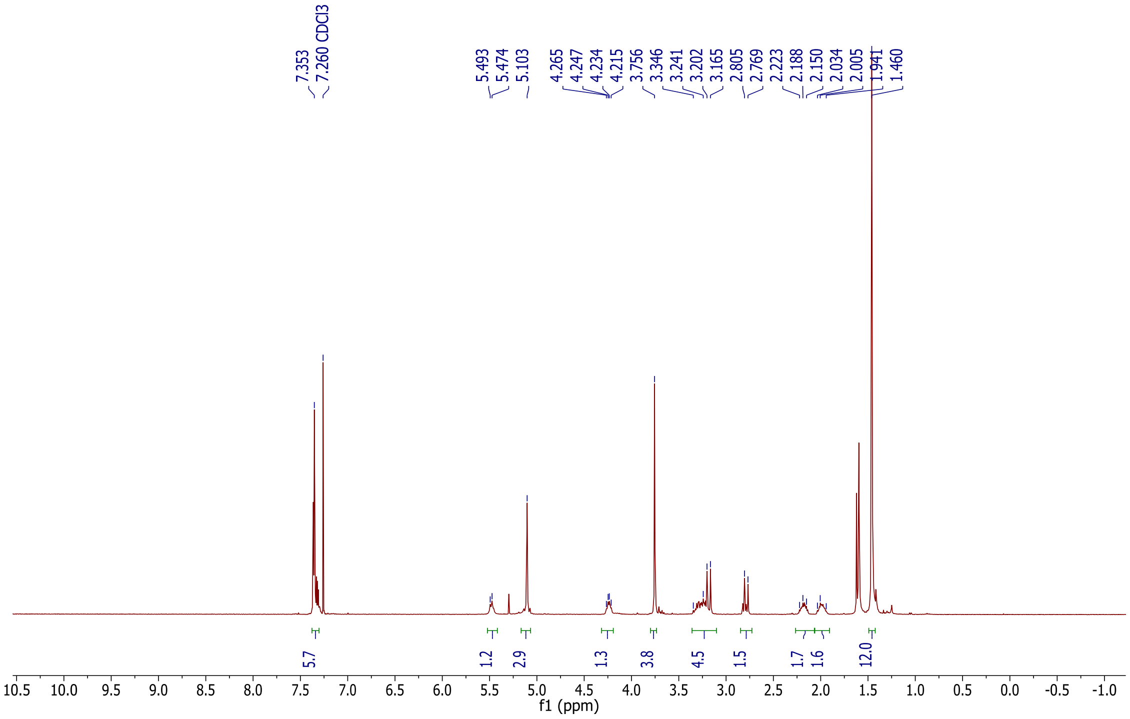

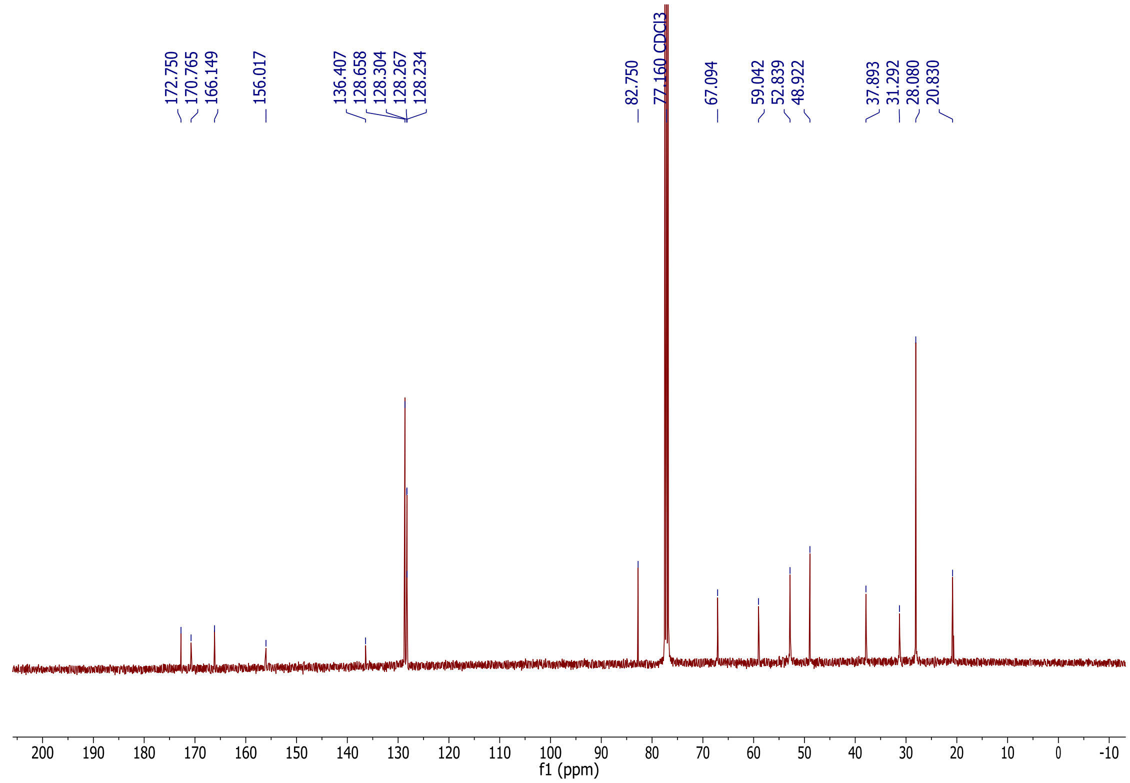

^{1}H-NMR (400 MHz, $CDCl_3$) and ^{13}C-NMR (75 MHz, $CDCl_3$) (45)

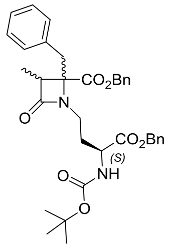

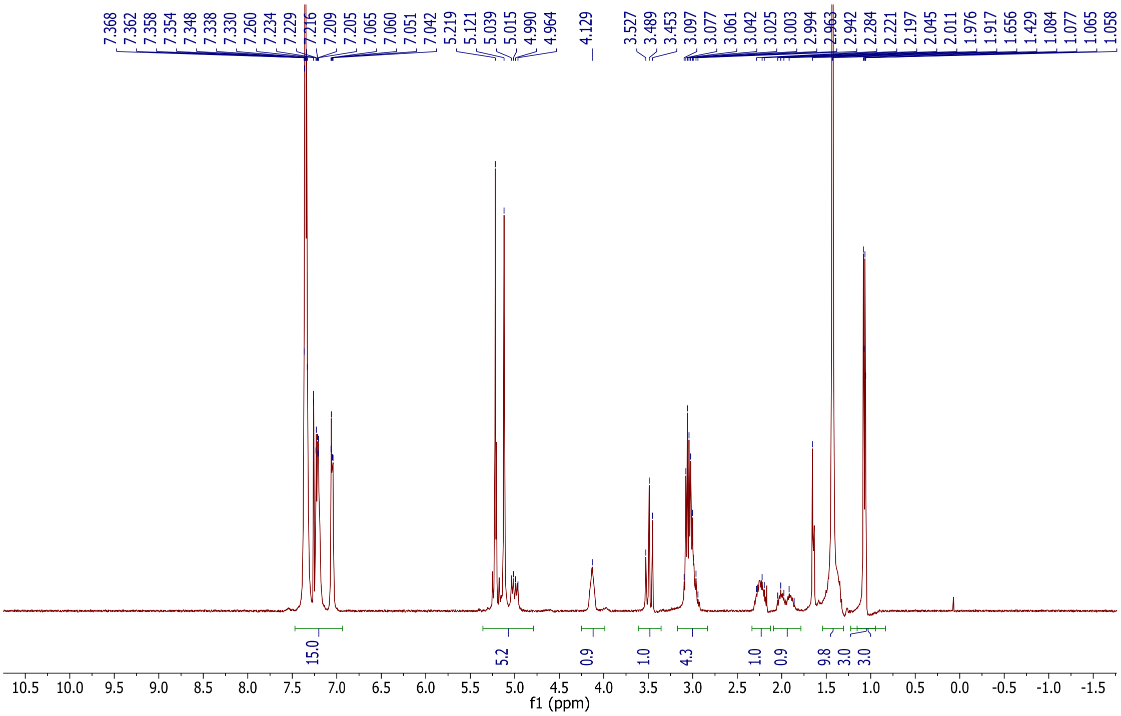

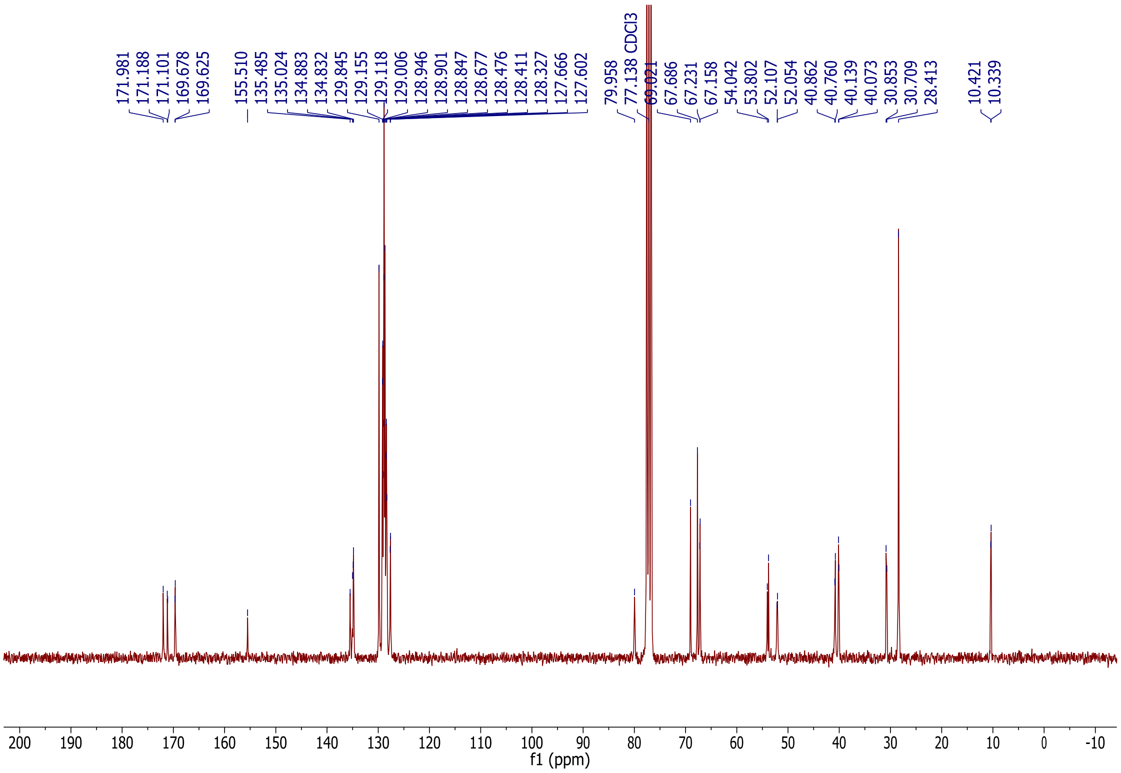

^{1}H-NMR (400 MHz, $CDCl_3$) and ^{13}C-NMR (75 MHz, $CDCl_3$) (46)

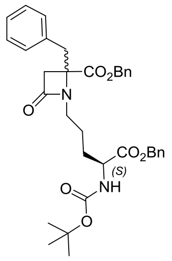

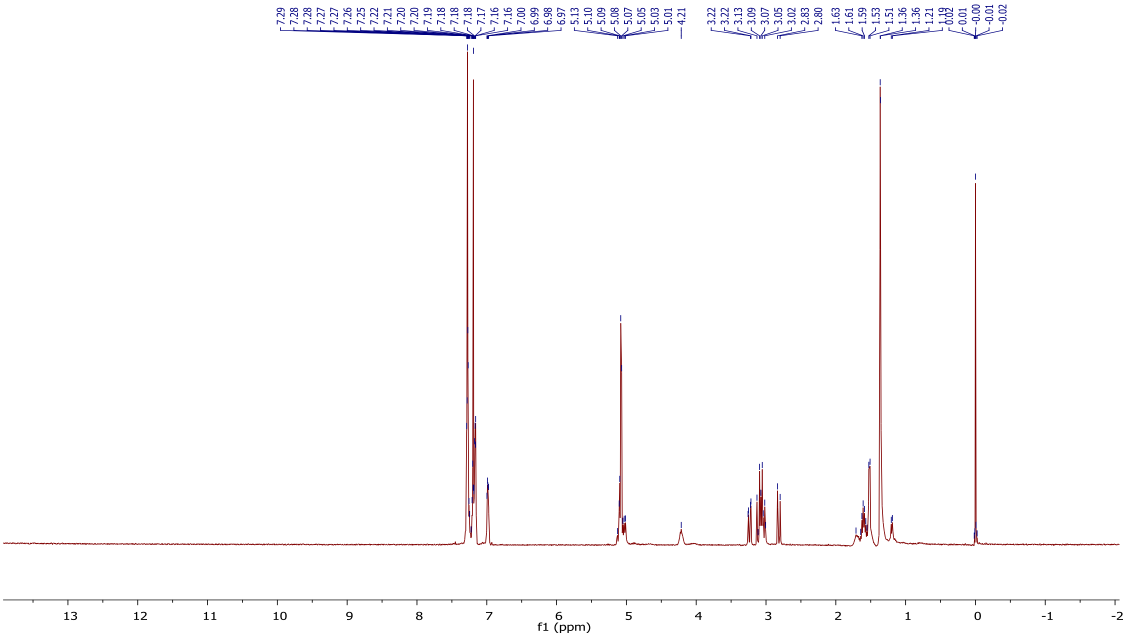

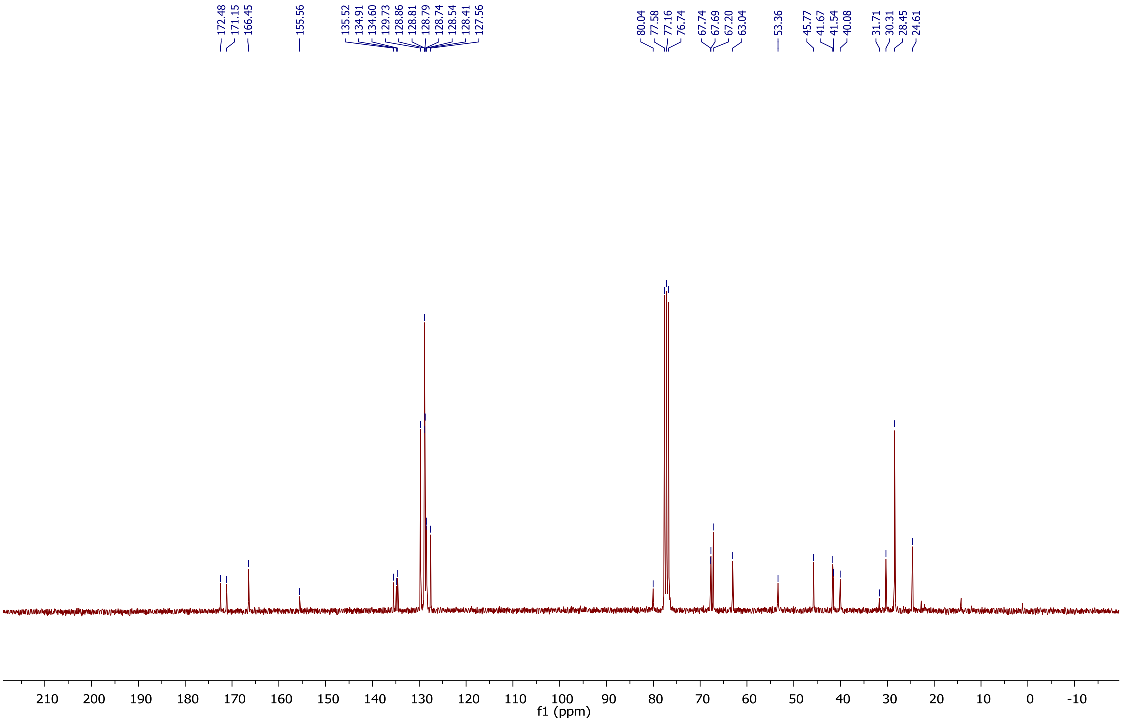

^{1}H-NMR (300 MHz, $CDCl_3$) (47)

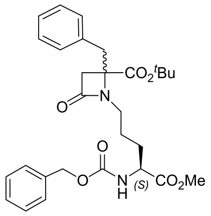

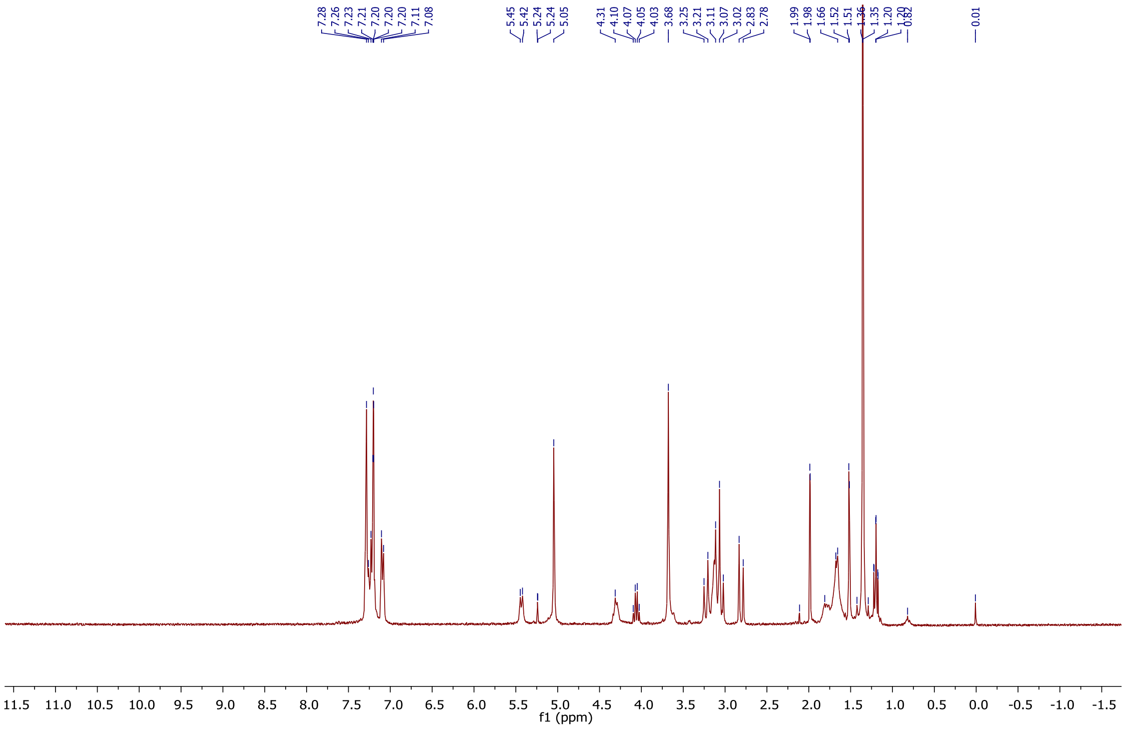

^{1}H-NMR (400 MHz, $CDCl_3$) and ^{13}C-NMR (75 MHz, $CDCl_3$) (48)

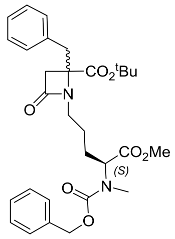

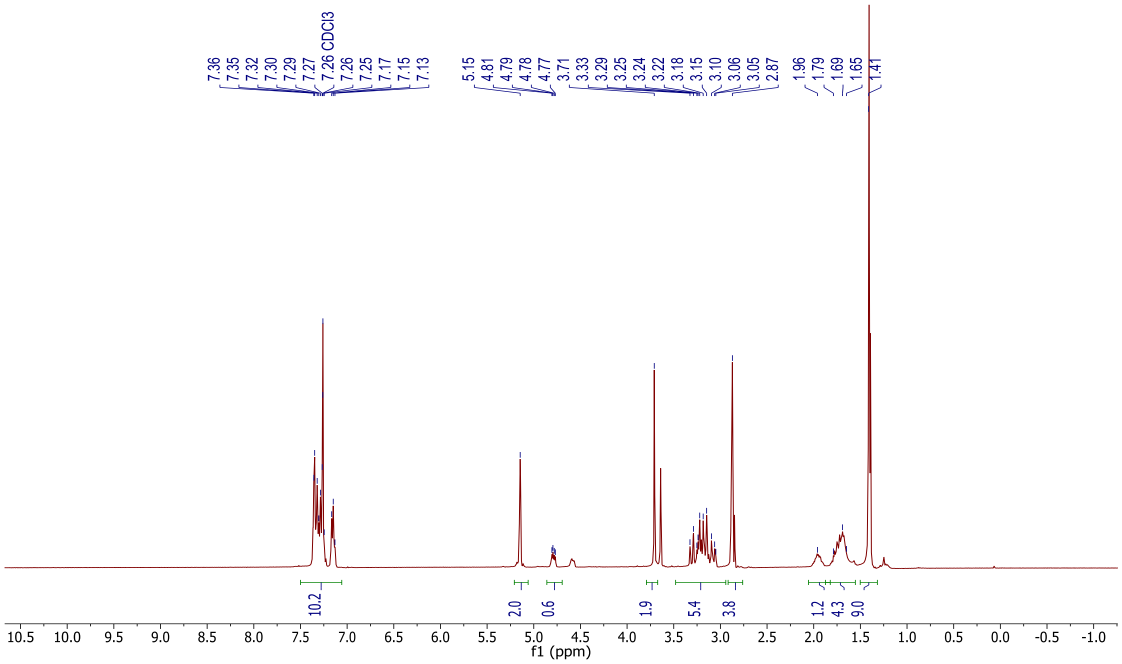

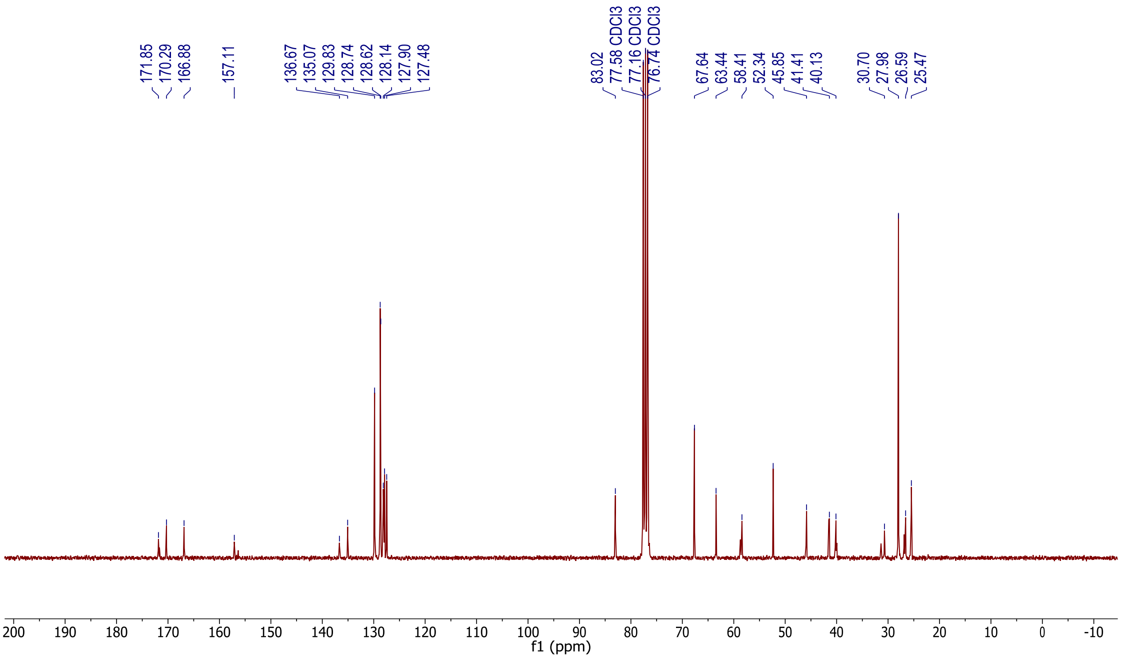

^{1}H-NMR (400 MHz, $CDCl_3$) and ^{13}C-NMR (75 MHz, $CDCl_3$) (49)

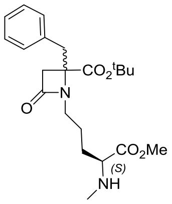

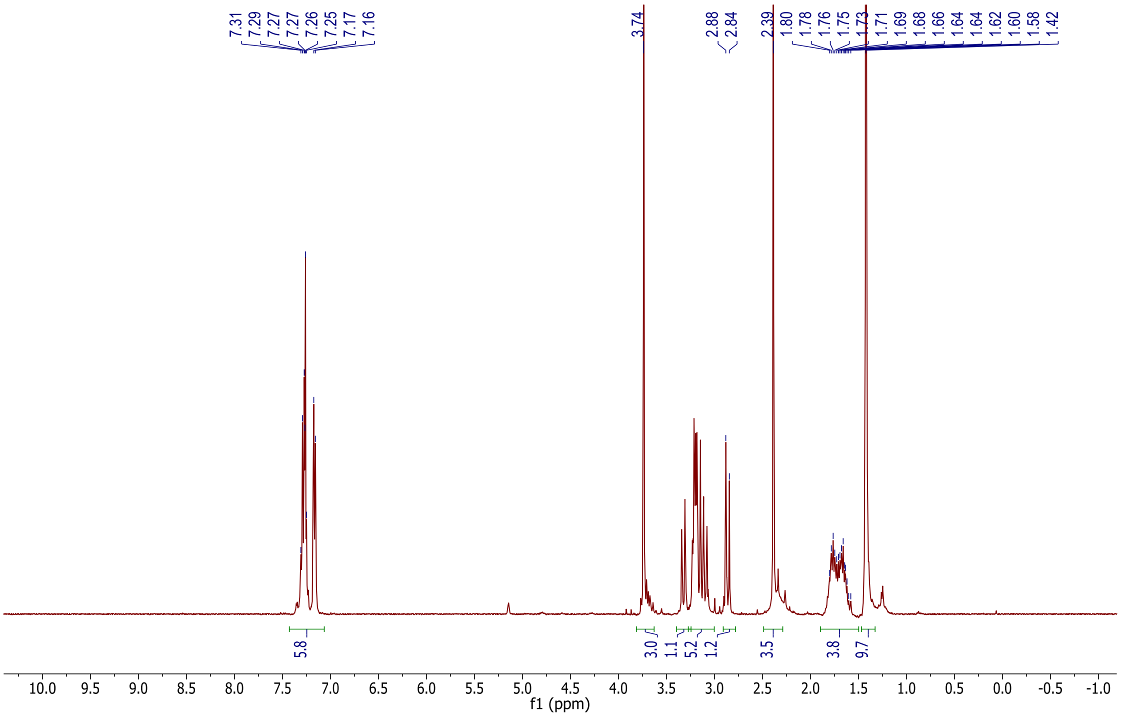

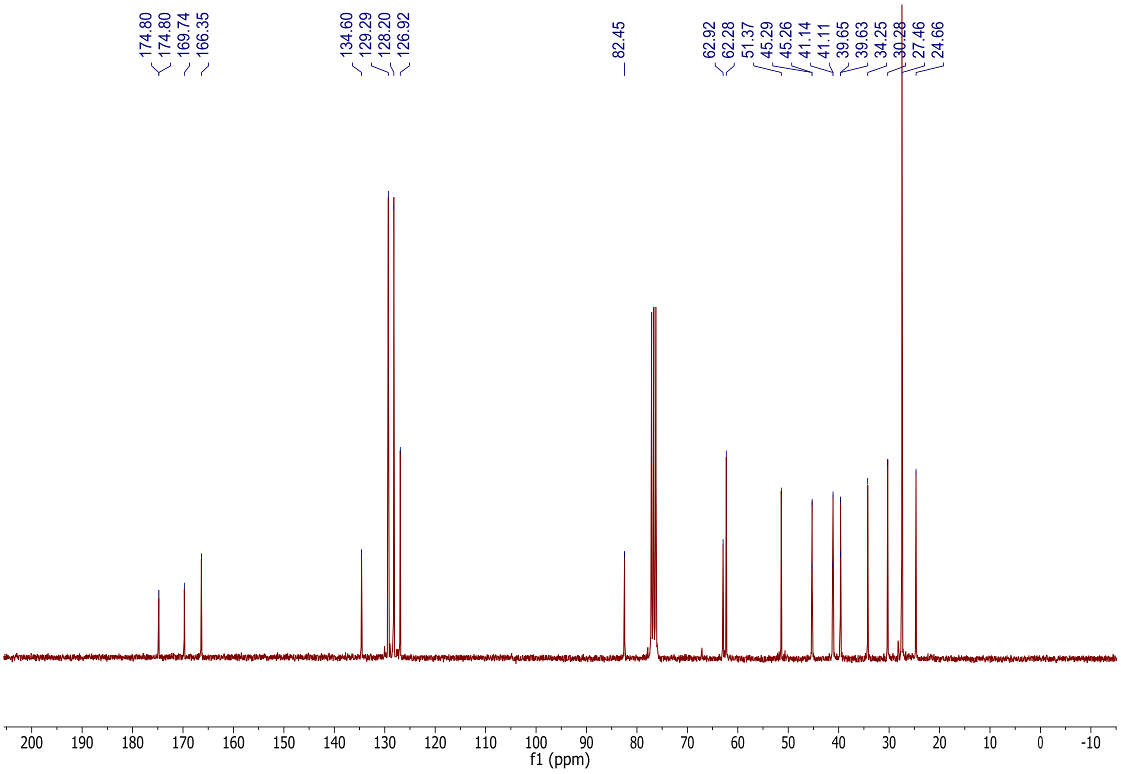

^{1}H-NMR (300 MHz, $CDCl_3$) (50)

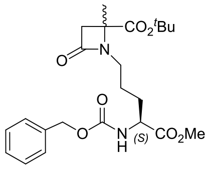

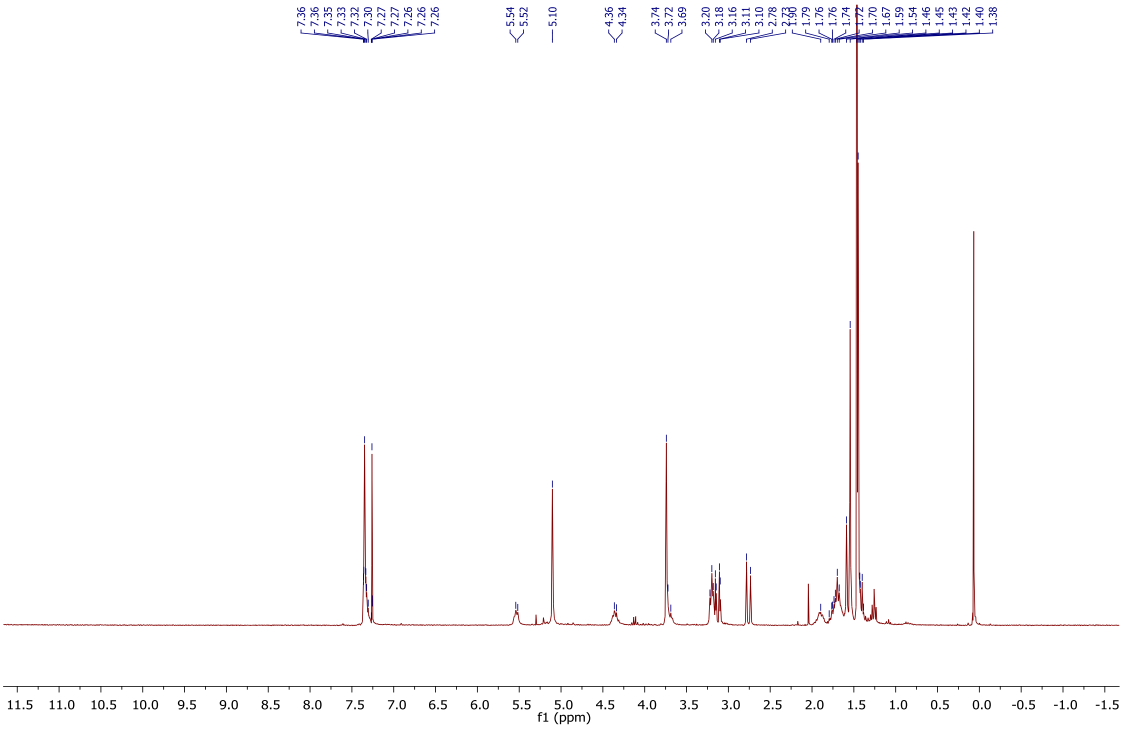

^{1}H-NMR (400 MHz, $CDCl_3$) and ^{13}C-NMR (75 MHz, $CDCl_3$) (51)

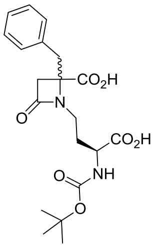

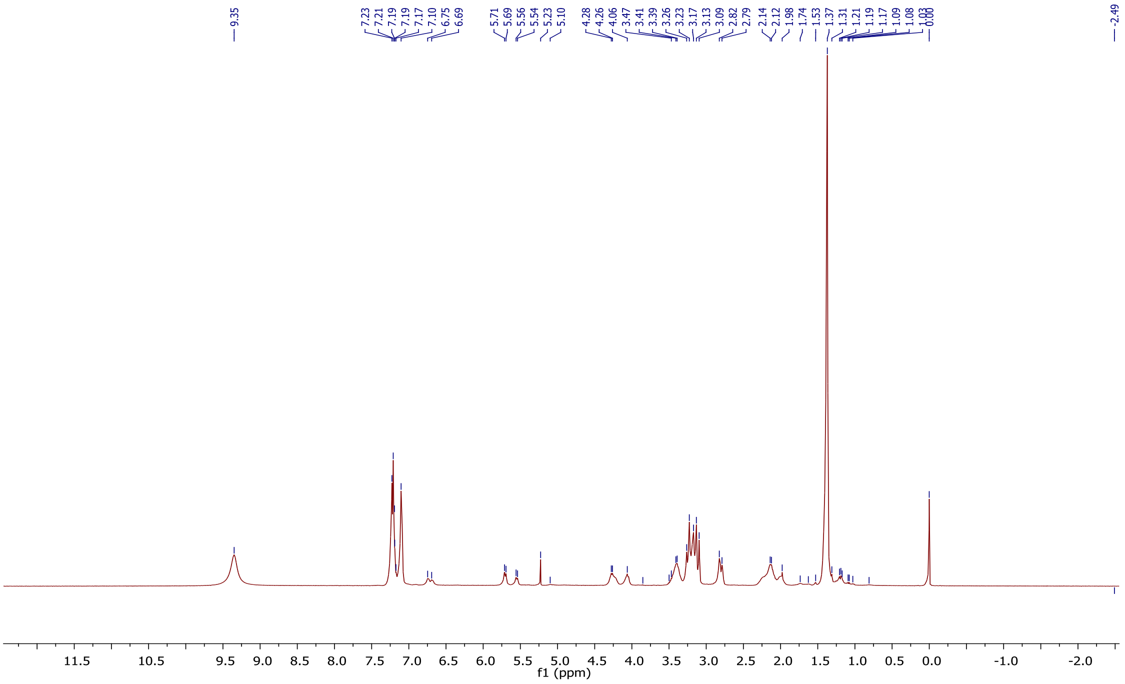

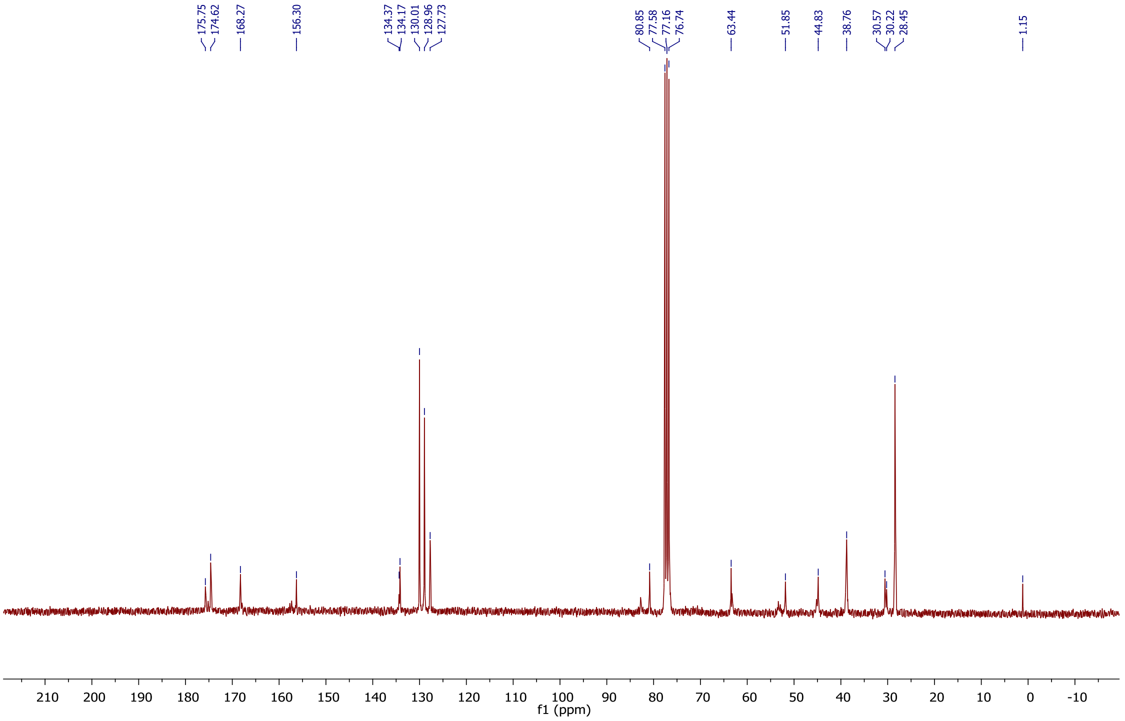

^{1}H-NMR (300 MHz, $CDCl_3$) and ^{13}C-NMR (75 MHz, $CDCl_3$) (53)

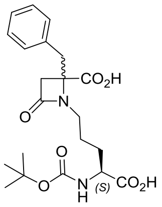

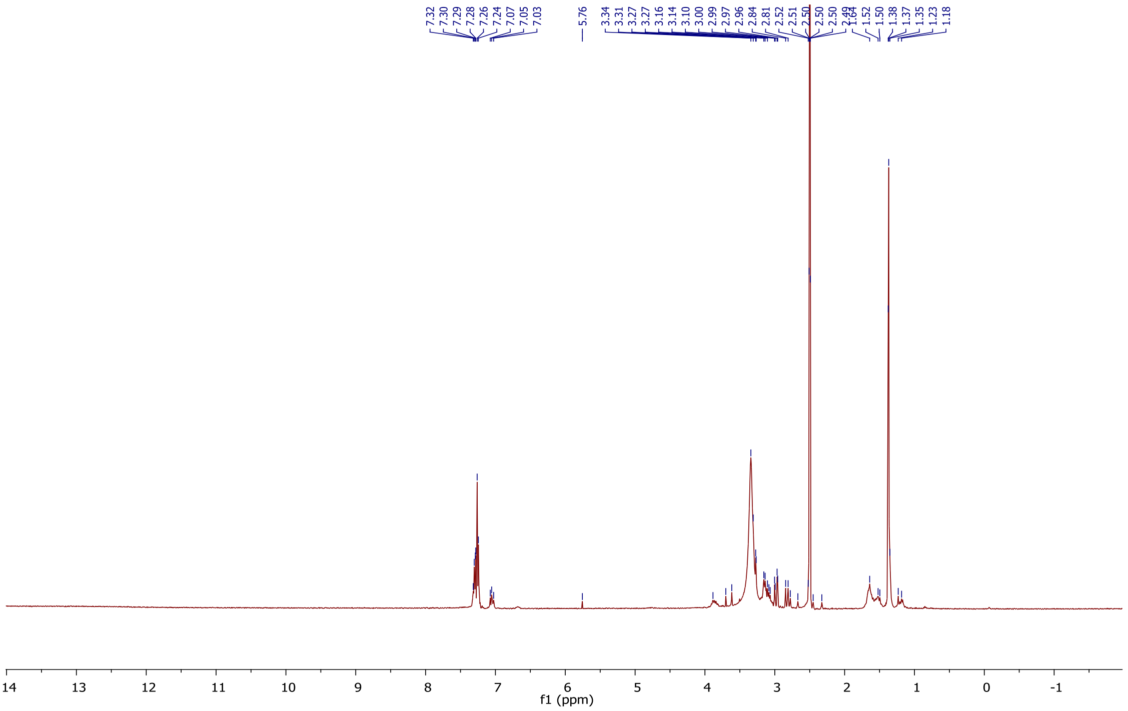

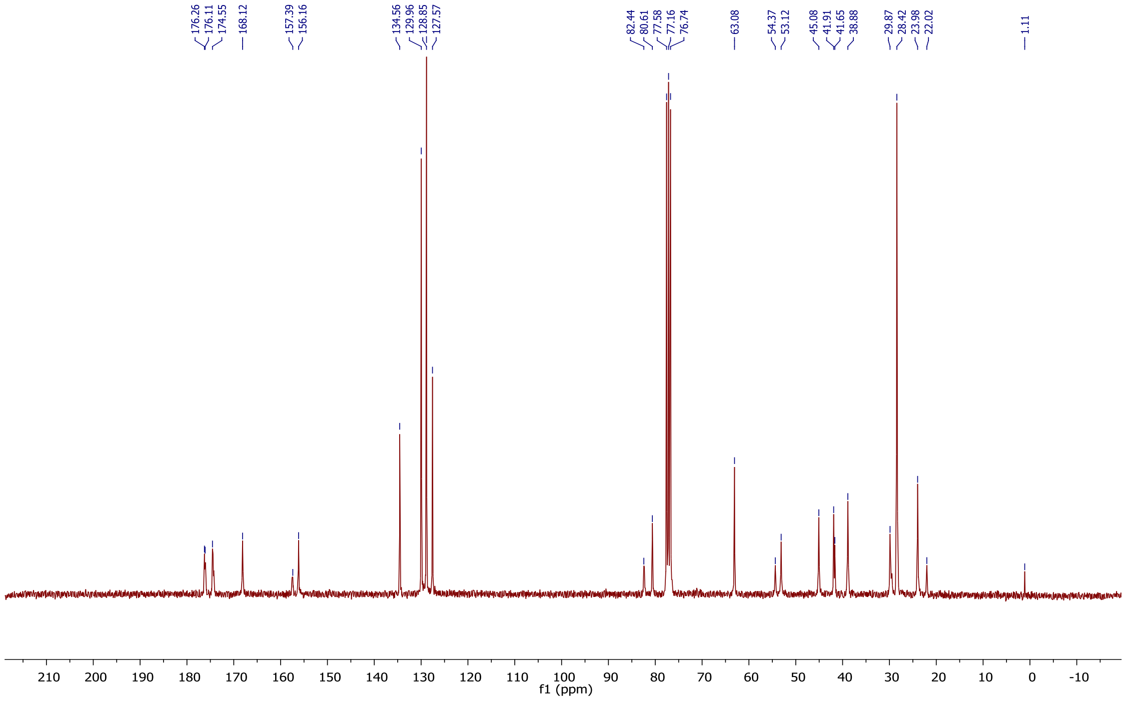

^{1}H-NMR (400 MHz, $CDCl_3$) and ^{13}C-NMR (75 MHz, $CDCl_3$) (56a)

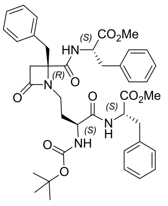

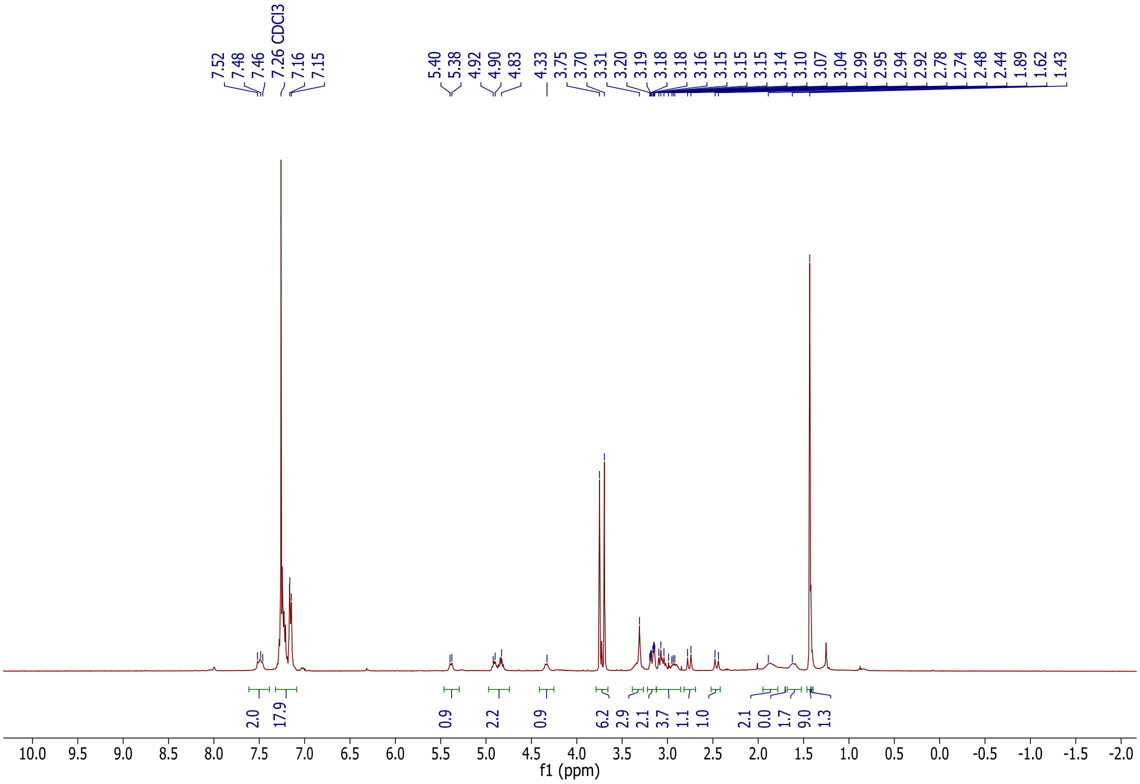

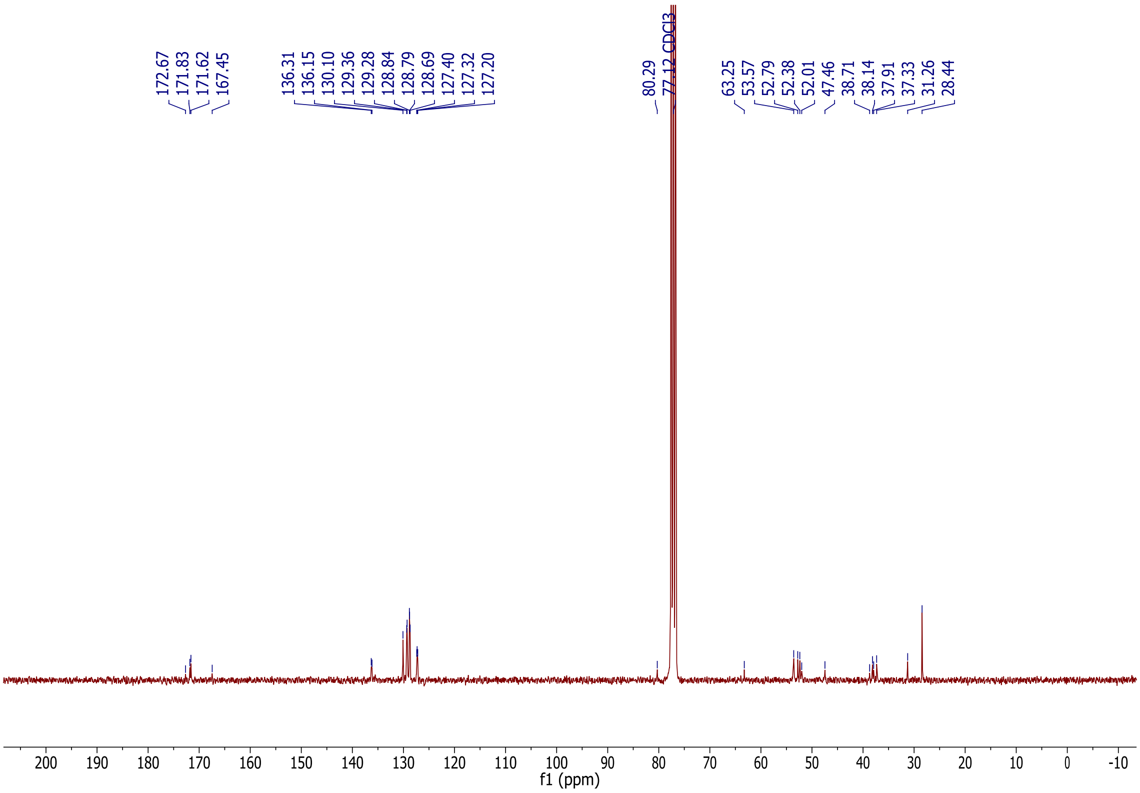

^{1}H-NMR (400 MHz, $CDCl_3$) and ^{13}C-NMR (75 MHz, $CDCl_3$) (56b)

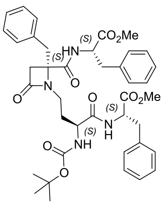

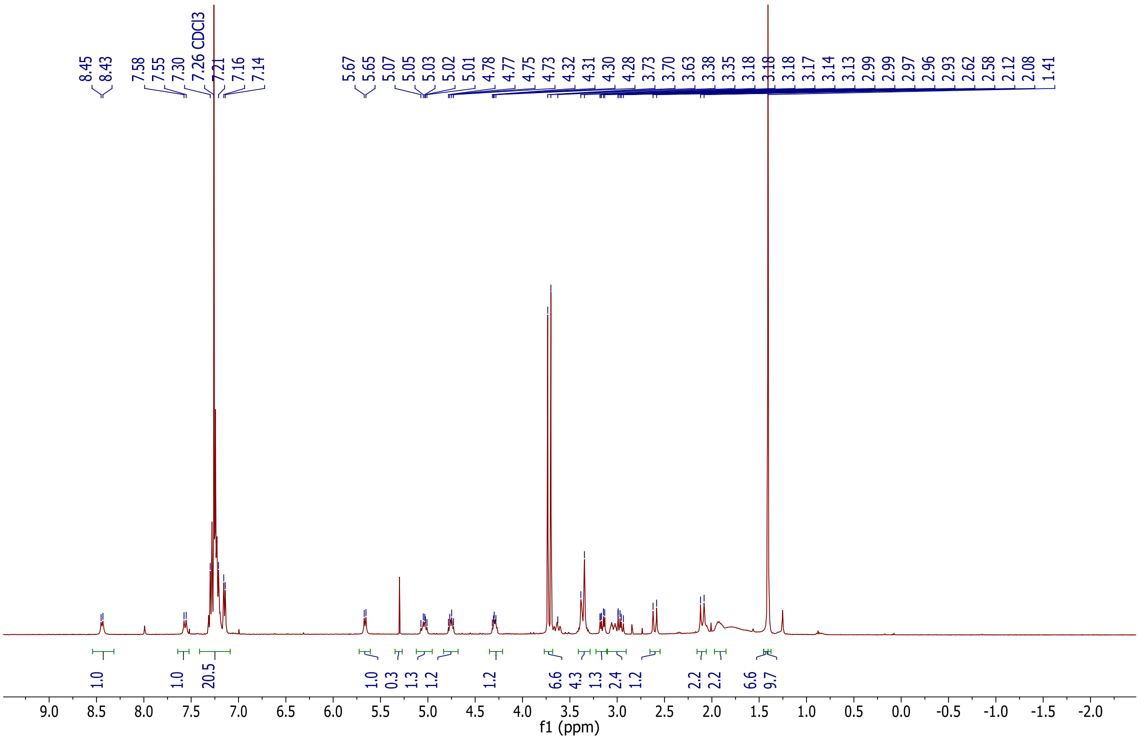

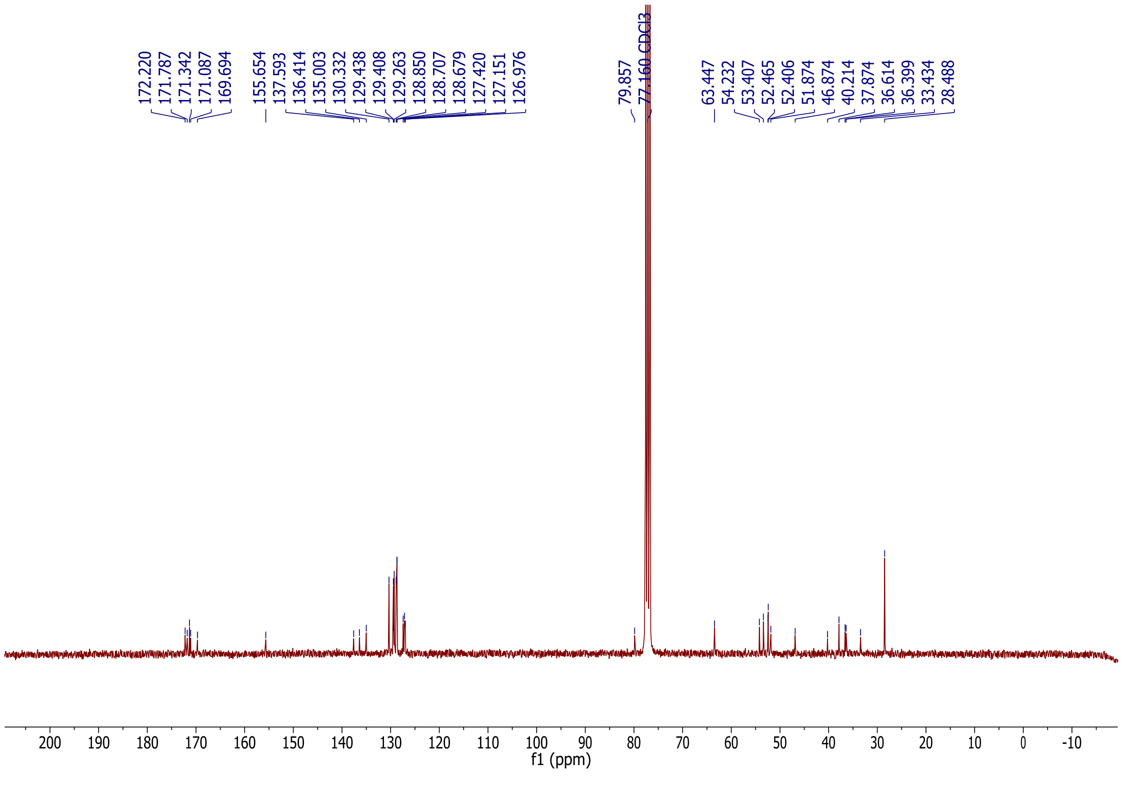

^{1}H-NMR (400 MHz, $CDCl_3$) and ^{13}C-NMR (75 MHz, $CDCl_3$) (57a)

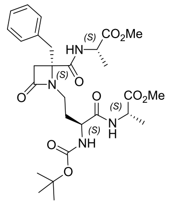

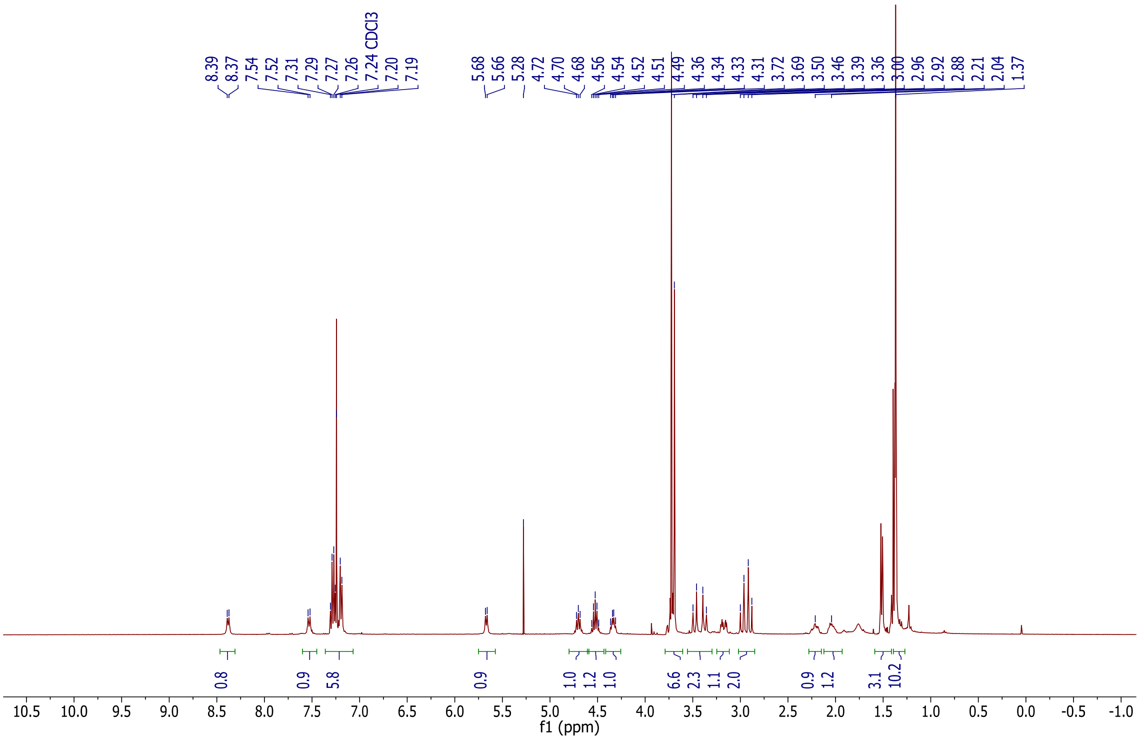

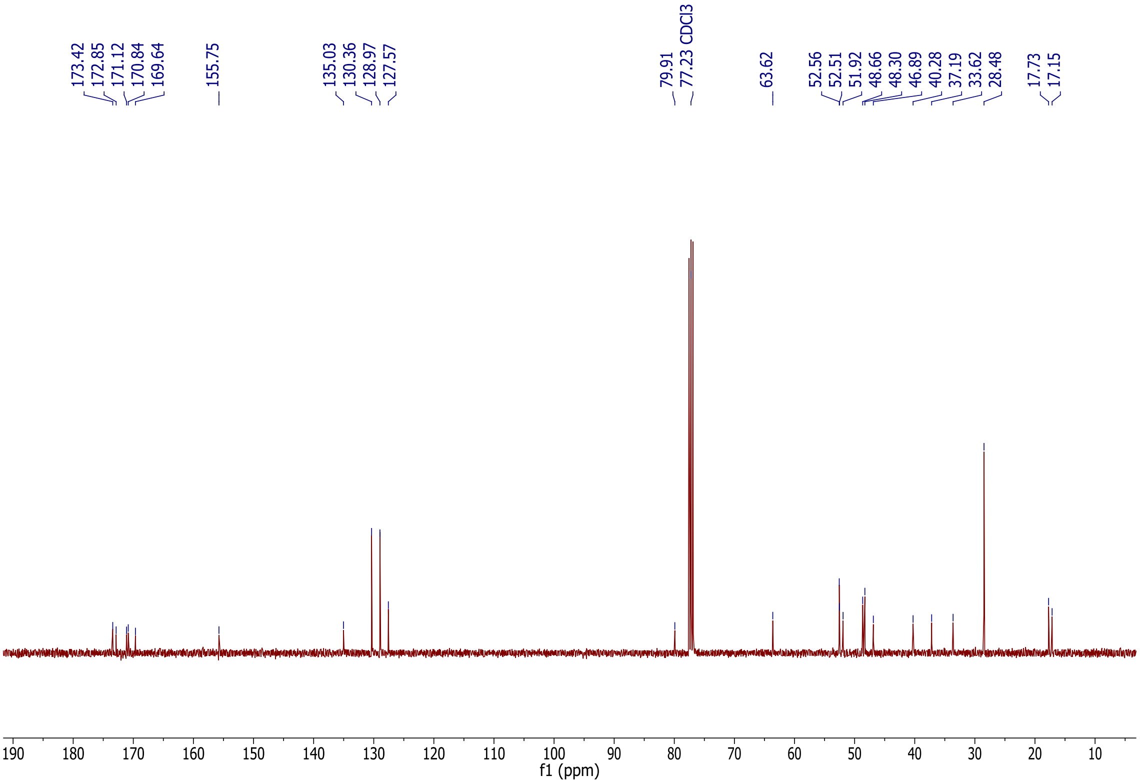

^{1}H-NMR (400 MHz, $CDCl_3$) and ^{13}C-NMR (75 MHz, $CDCl_3$) (57b)

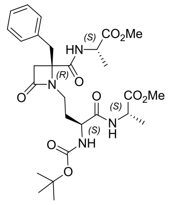

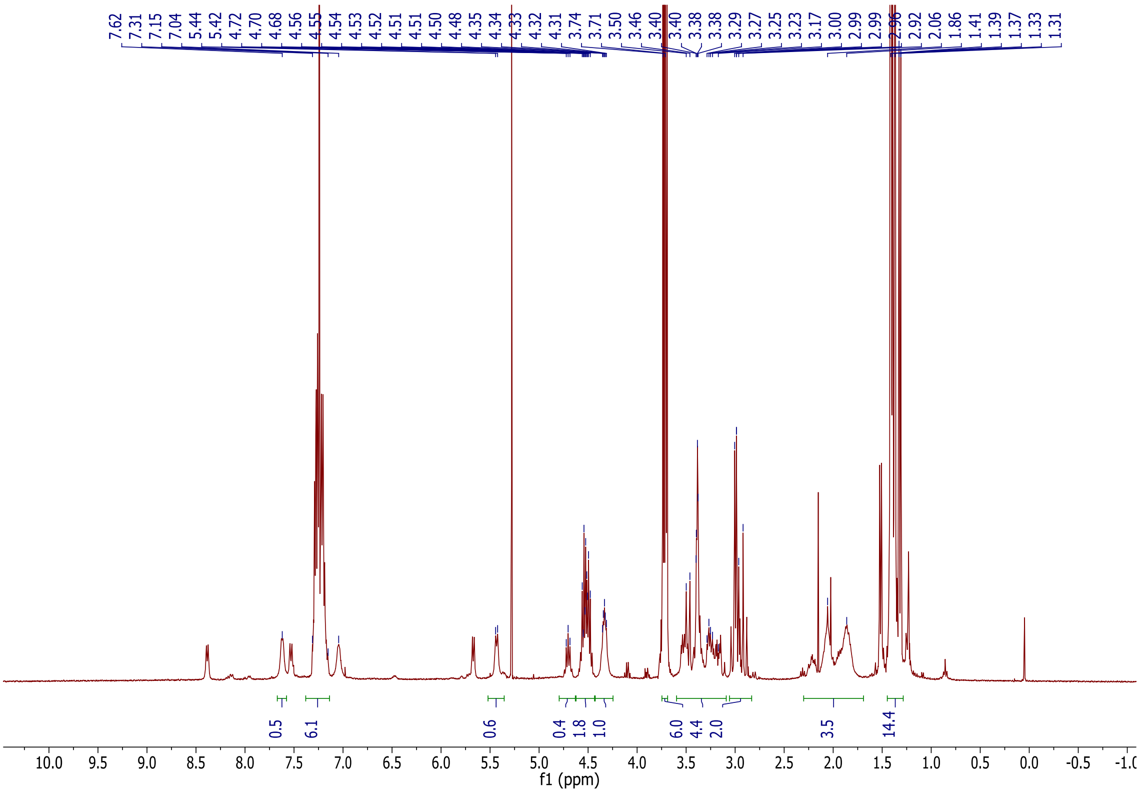

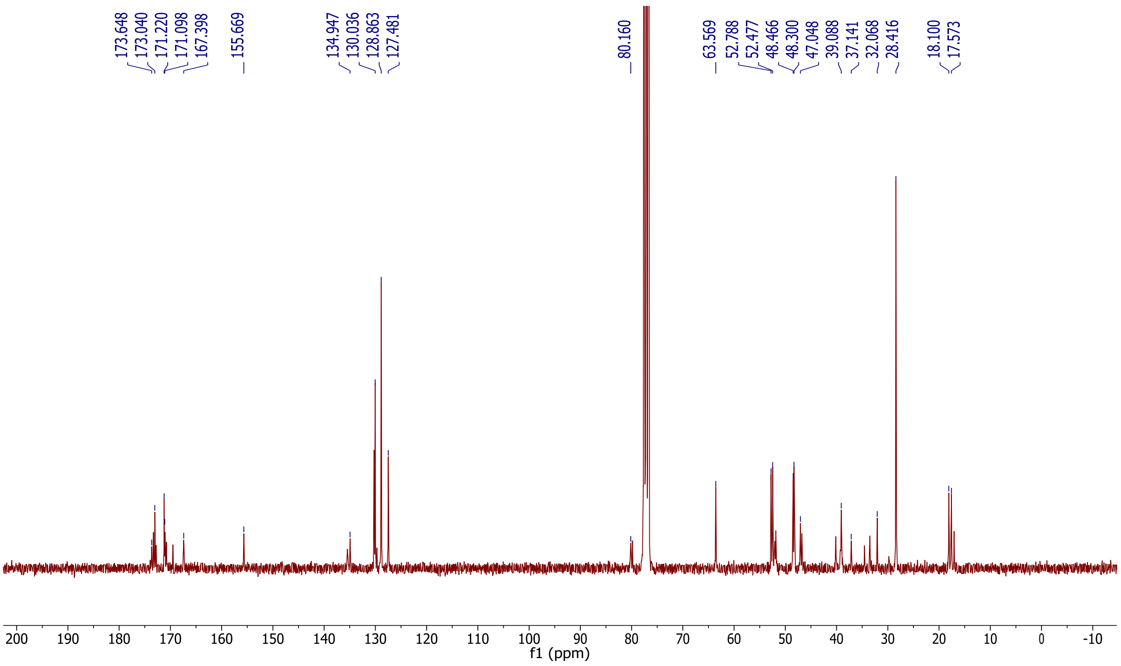

^{1}H-NMR (300 MHz, $CDCl_3$) (58)

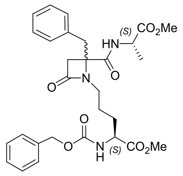

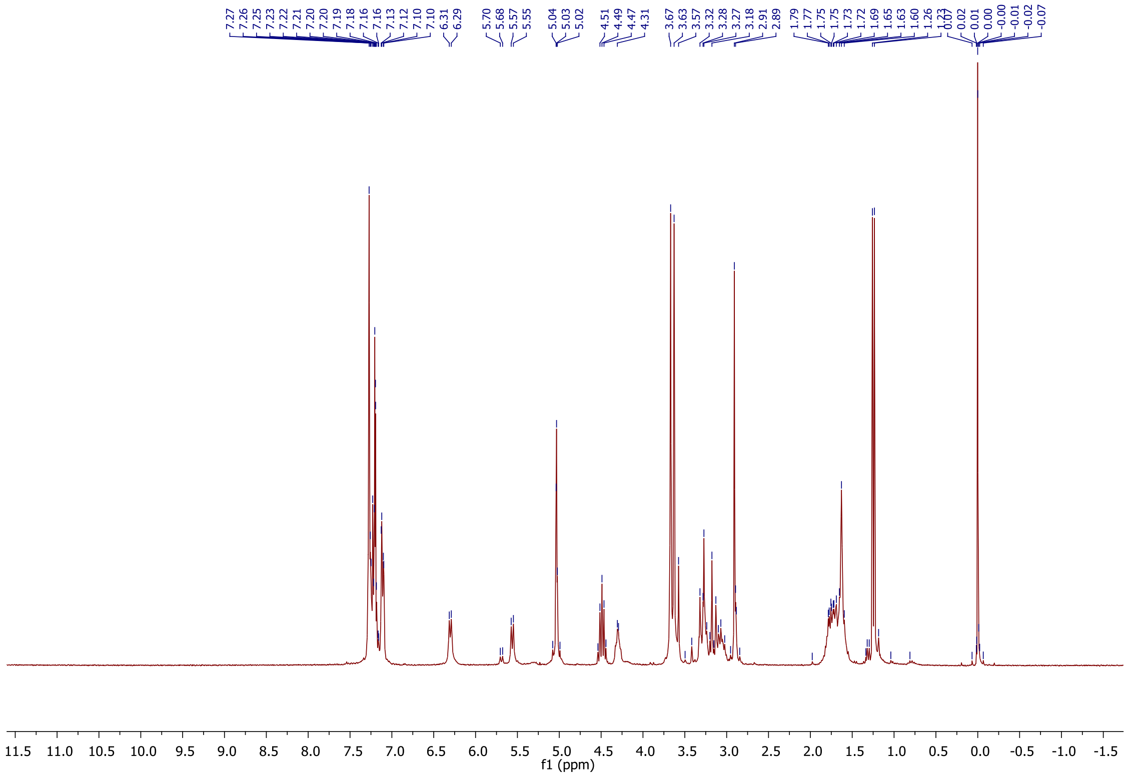

^{1}H-NMR (300 MHz, $CDCl_3$) (59)

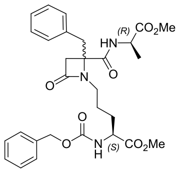

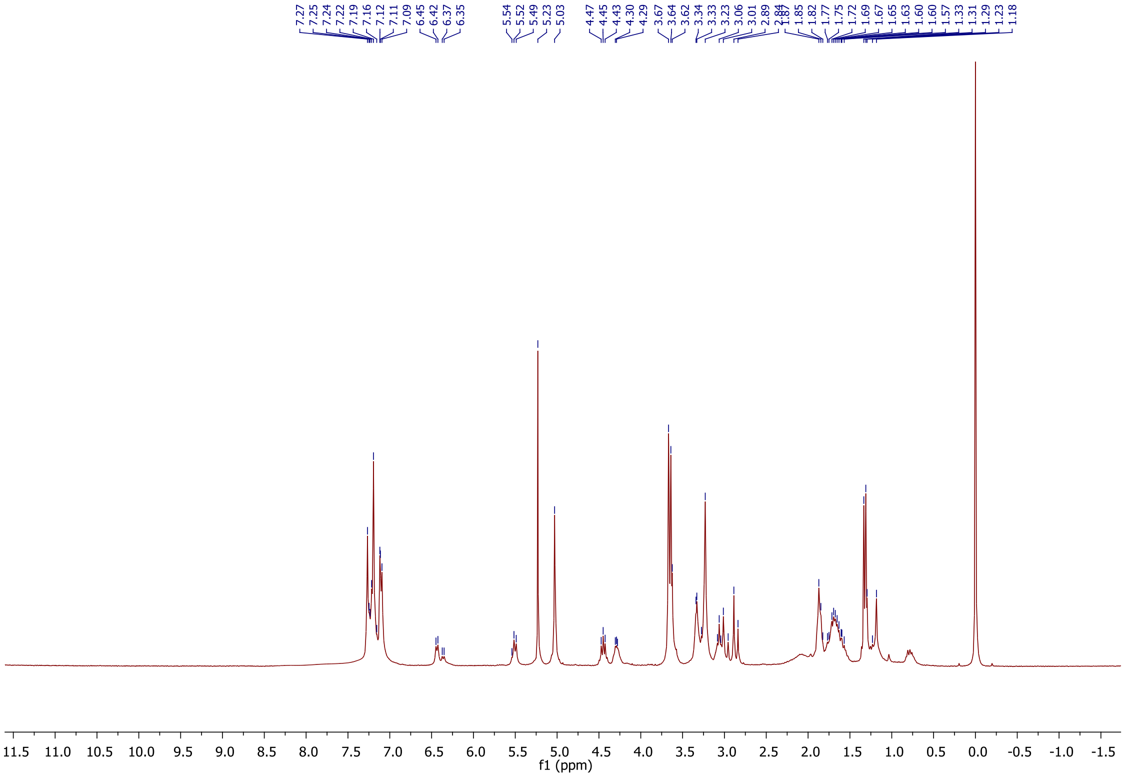

^{1}H-NMR (400 MHz, CDCl$_3$) and ^{13}C-NMR (75 MHz, CDCl$_3$) (60)

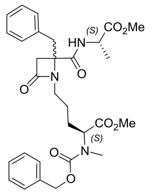

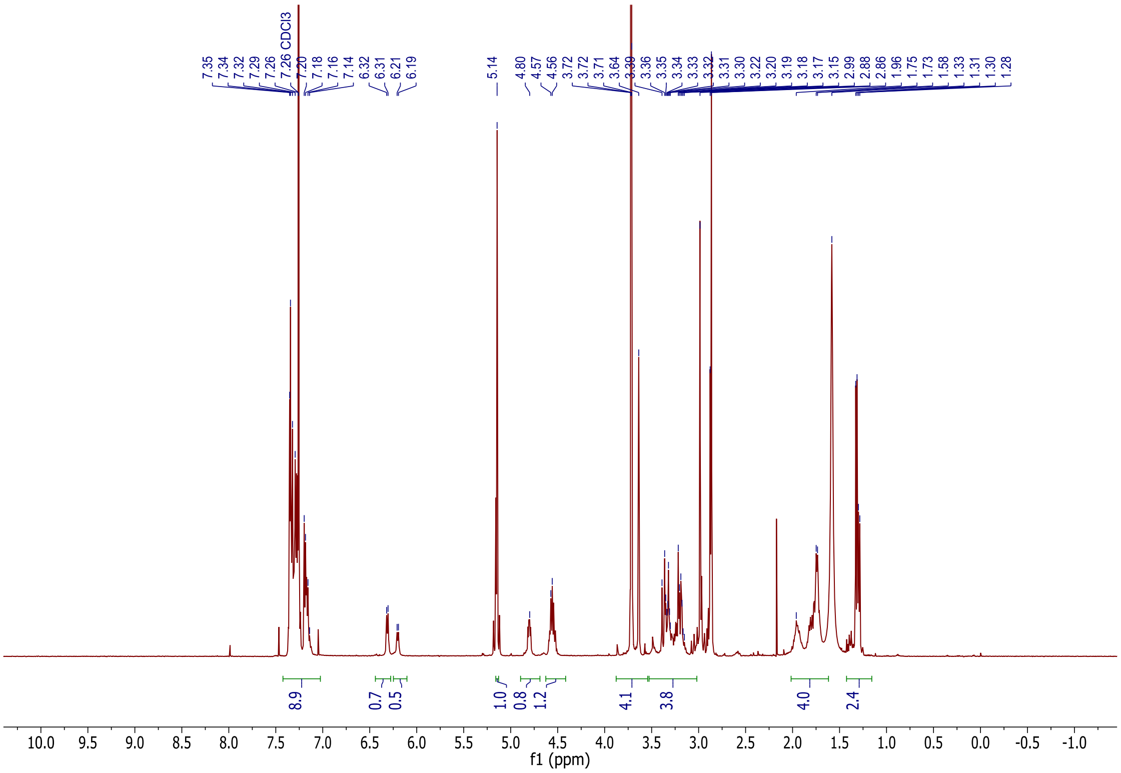

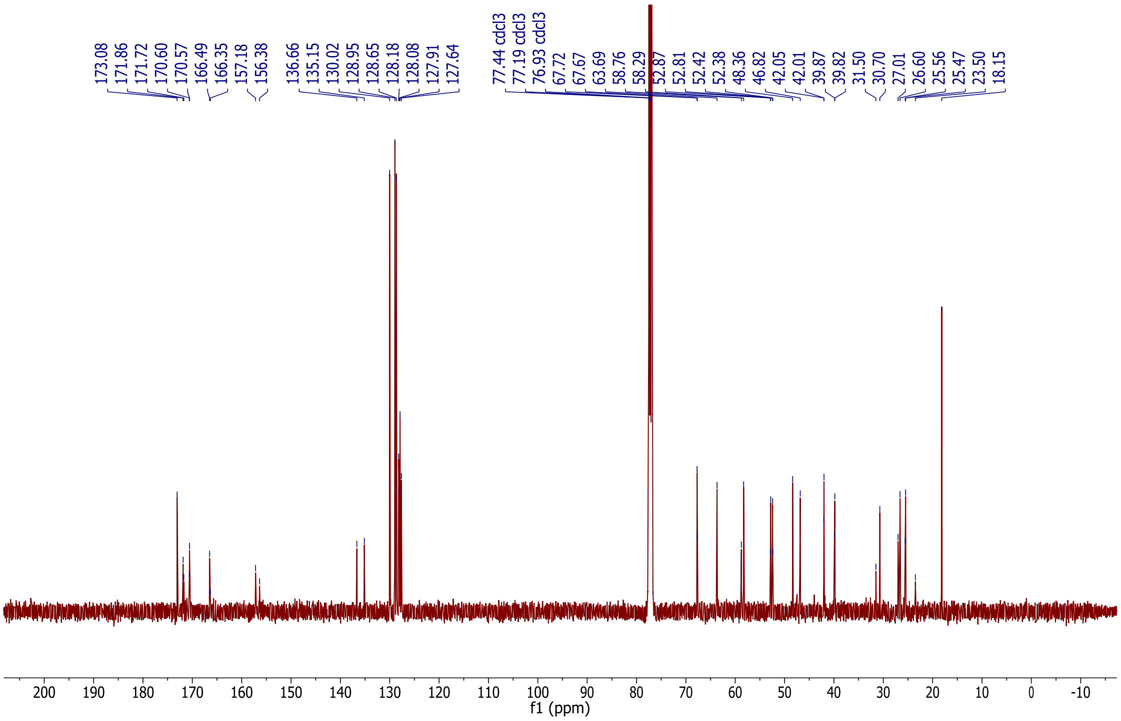

^{1}H-NMR (400 MHz, $CDCl_3$) and ^{13}C-NMR (75 MHz, $CDCl_3$) (61)

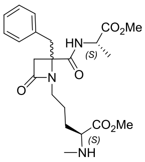

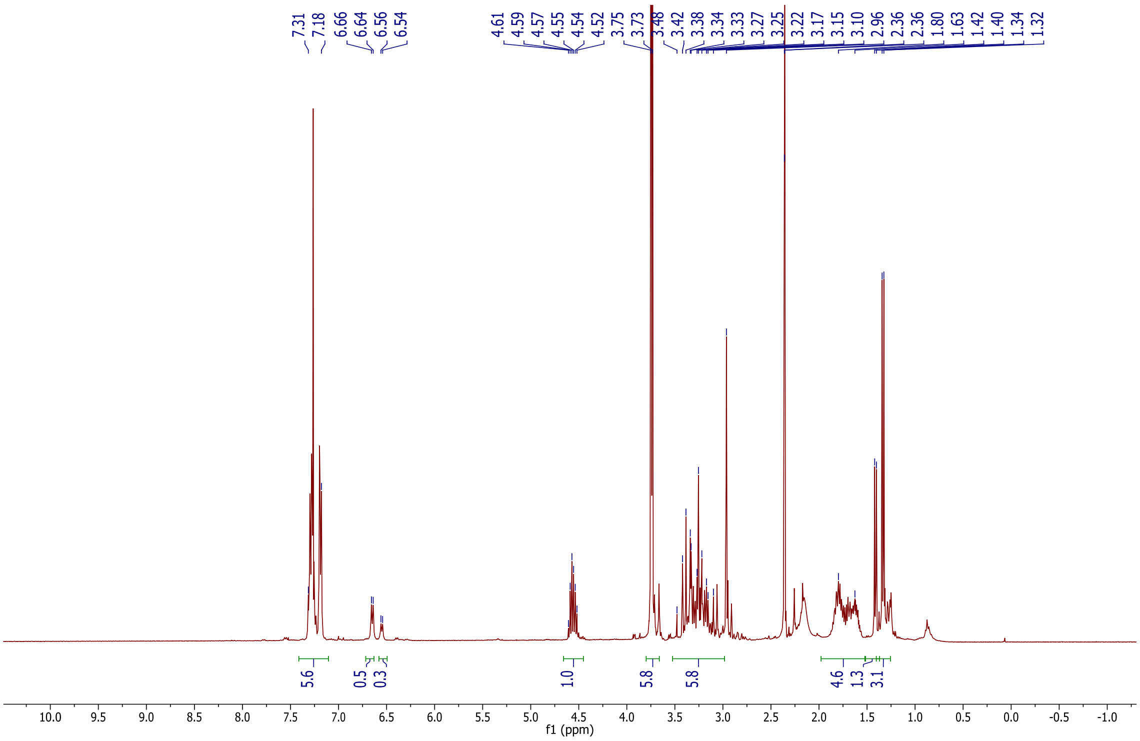

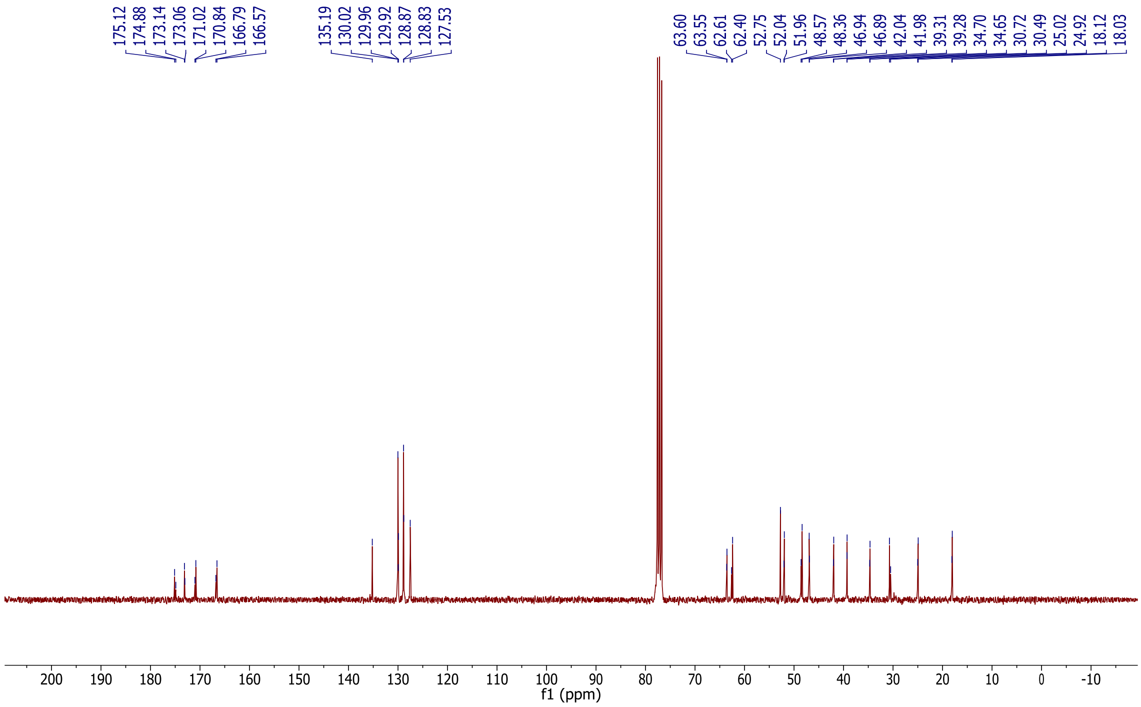

^{1}H-NMR (400 MHz, $CDCl_3$) and ^{13}C-NMR (75 MHz, $CDCl_3$) (62a)

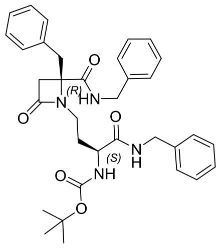

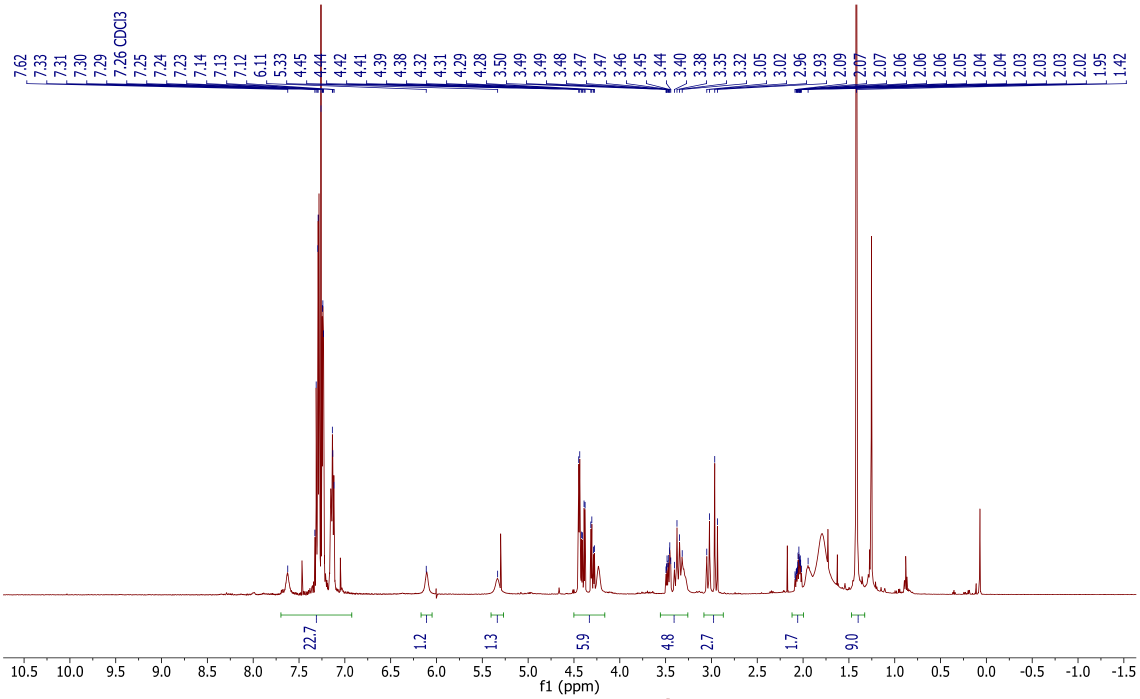

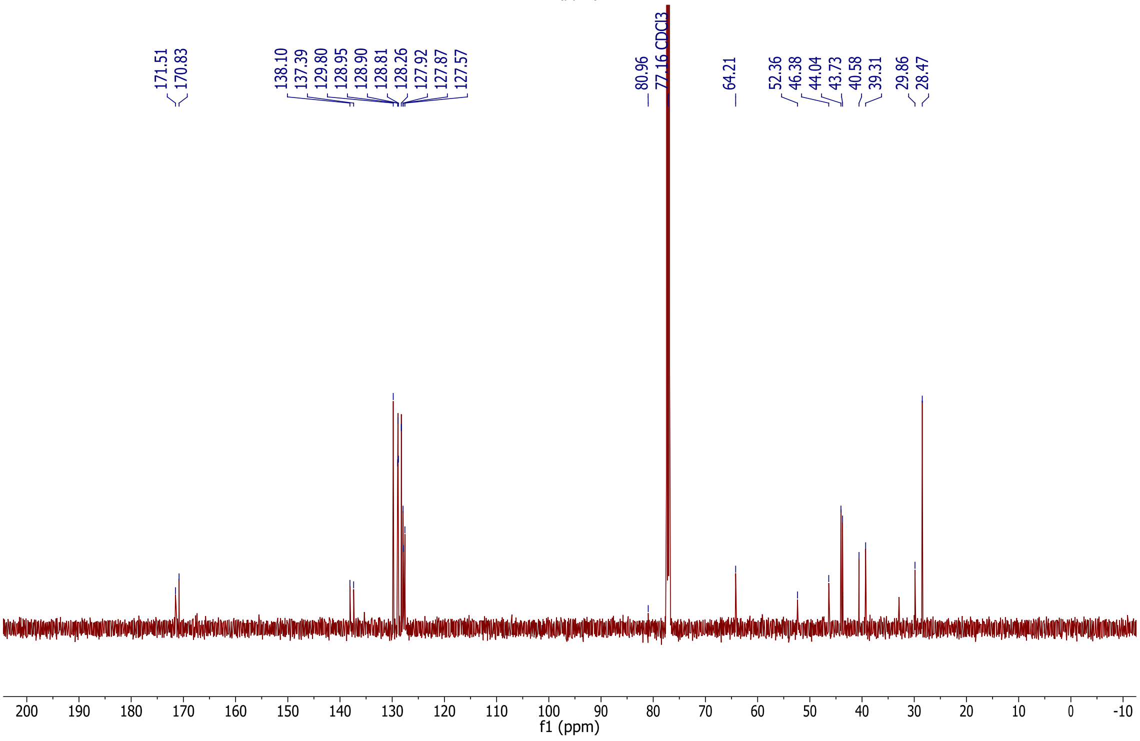

^{1}H-NMR (400 MHz, $CDCl_3$) and ^{13}C-NMR (75 MHz, $CDCl_3$) (63a)

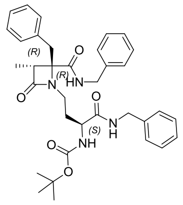

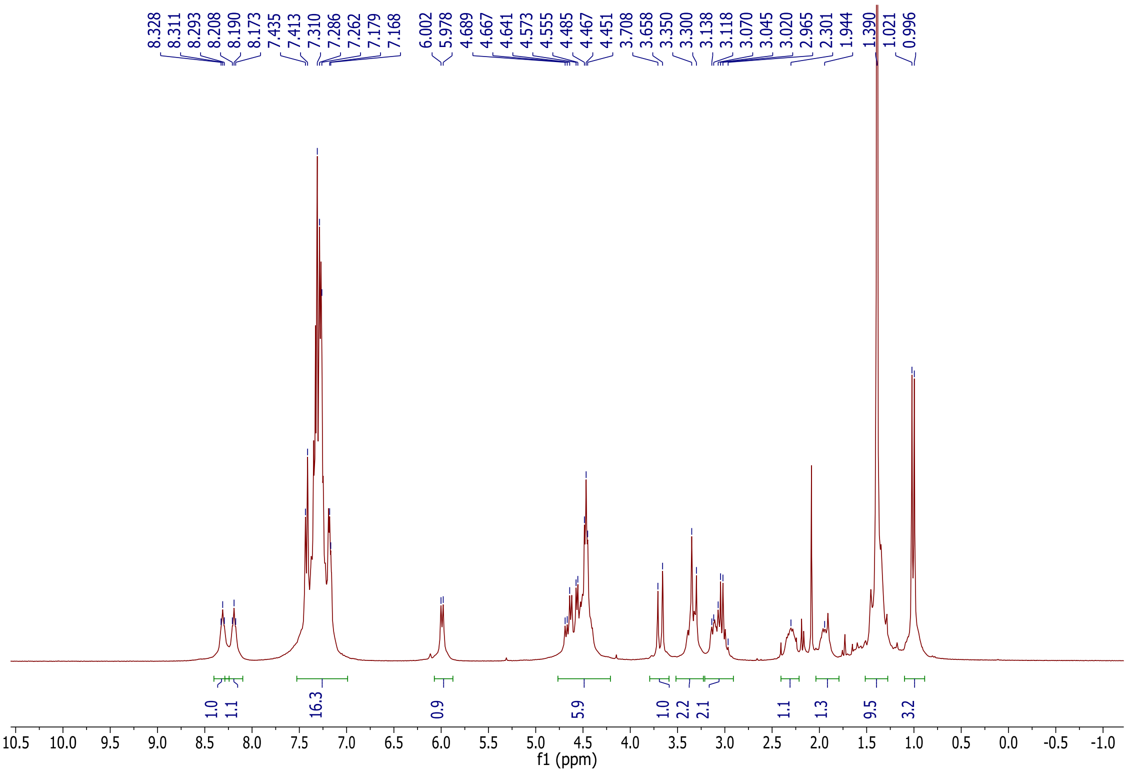

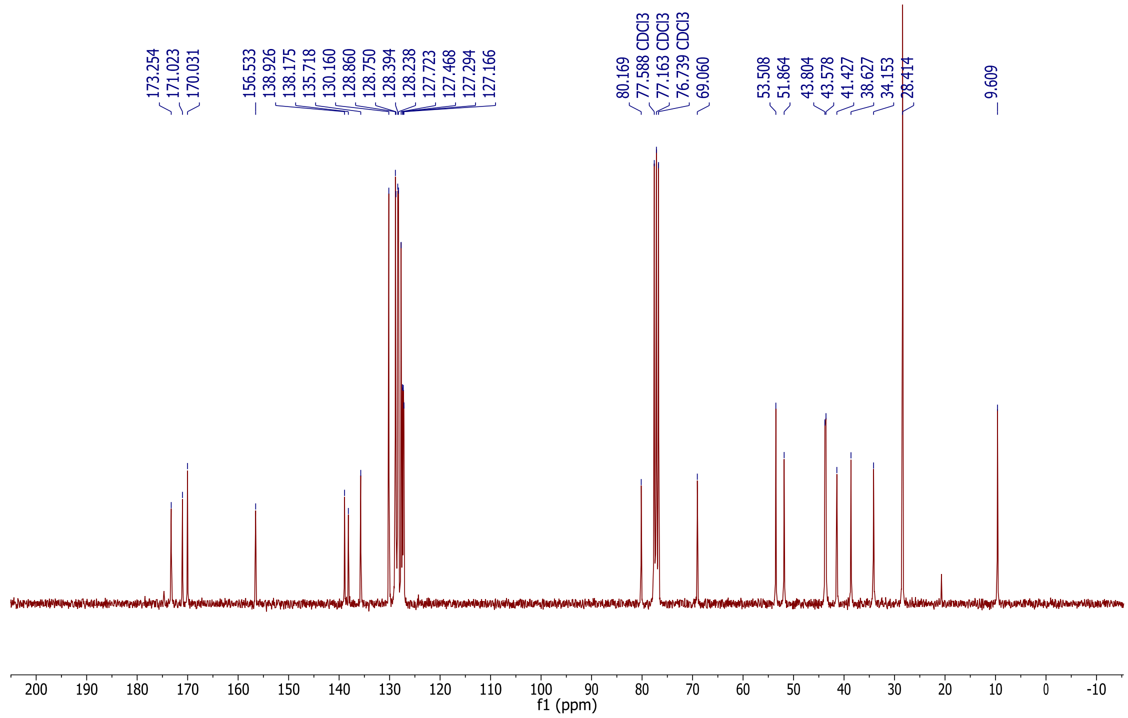

^{1}H-NMR (400 MHz, $CDCl_3$) and ^{13}C-NMR (75 MHz, $CDCl_3$) (63b)

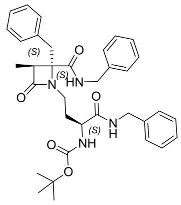

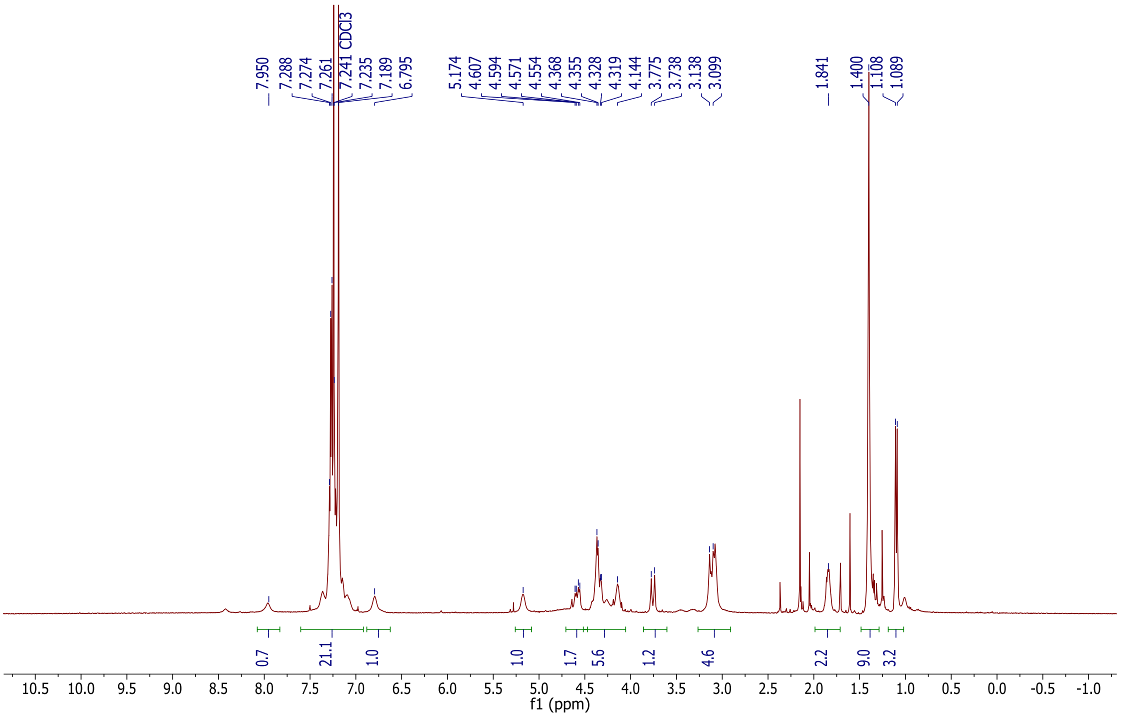

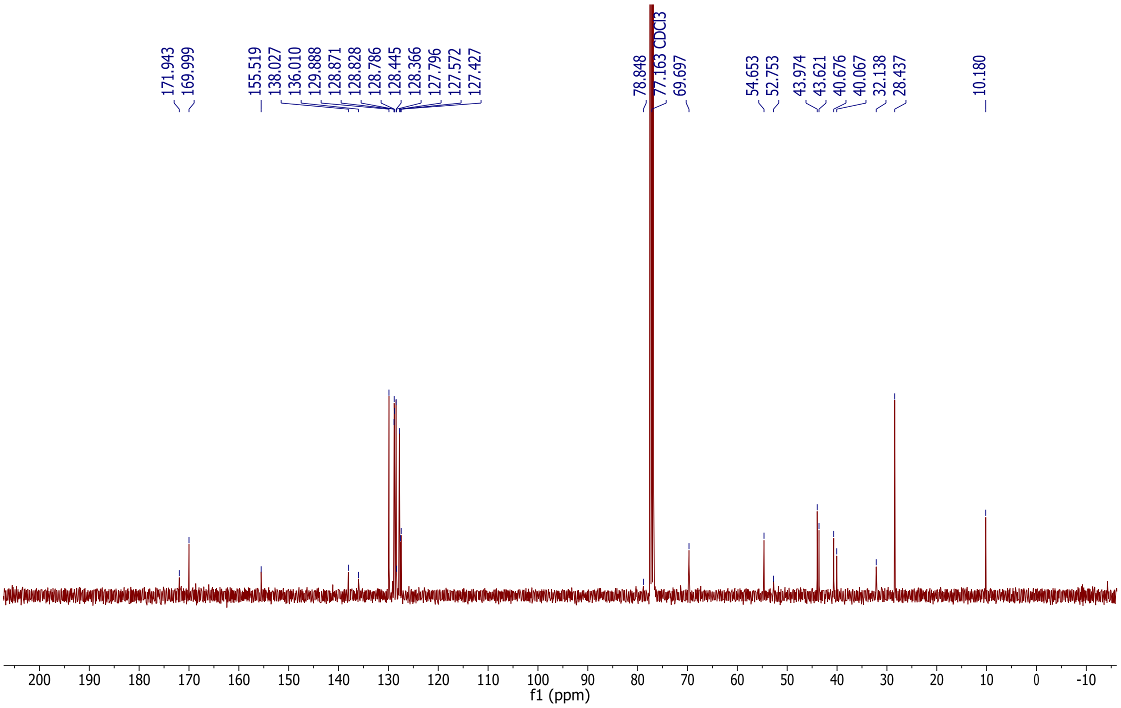

^{1}H-NMR (400 MHz, $CDCl_3$) and ^{13}C-NMR (75 MHz, $CDCl_3$) (64)

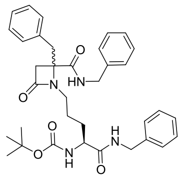

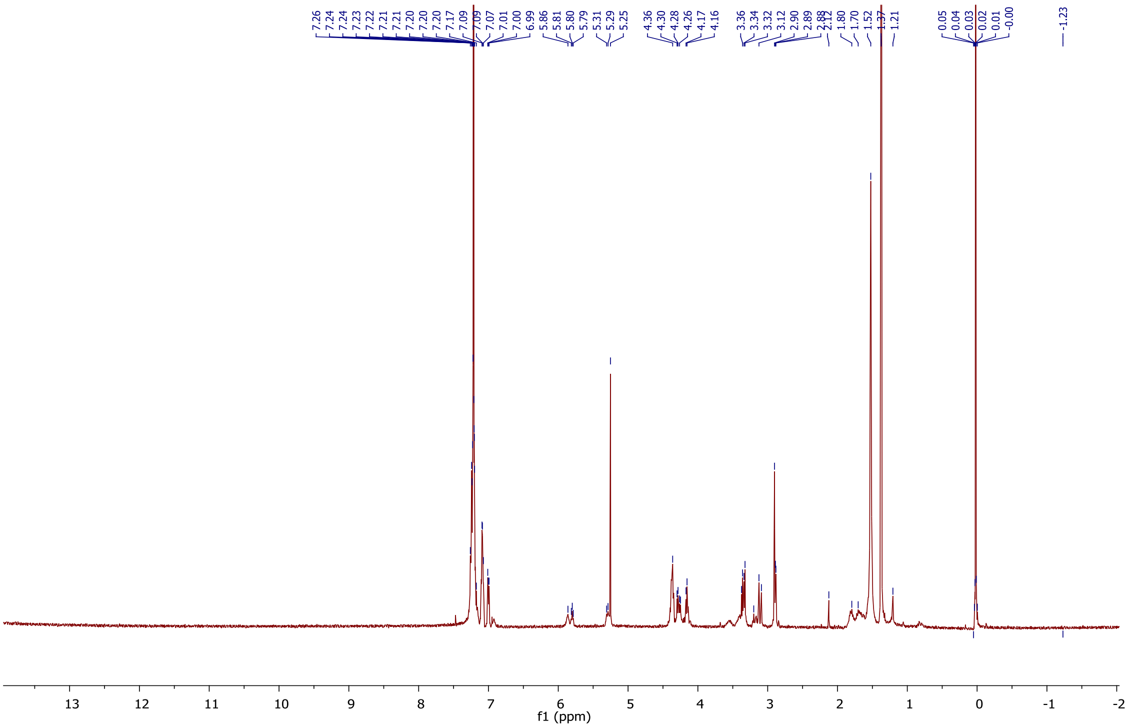

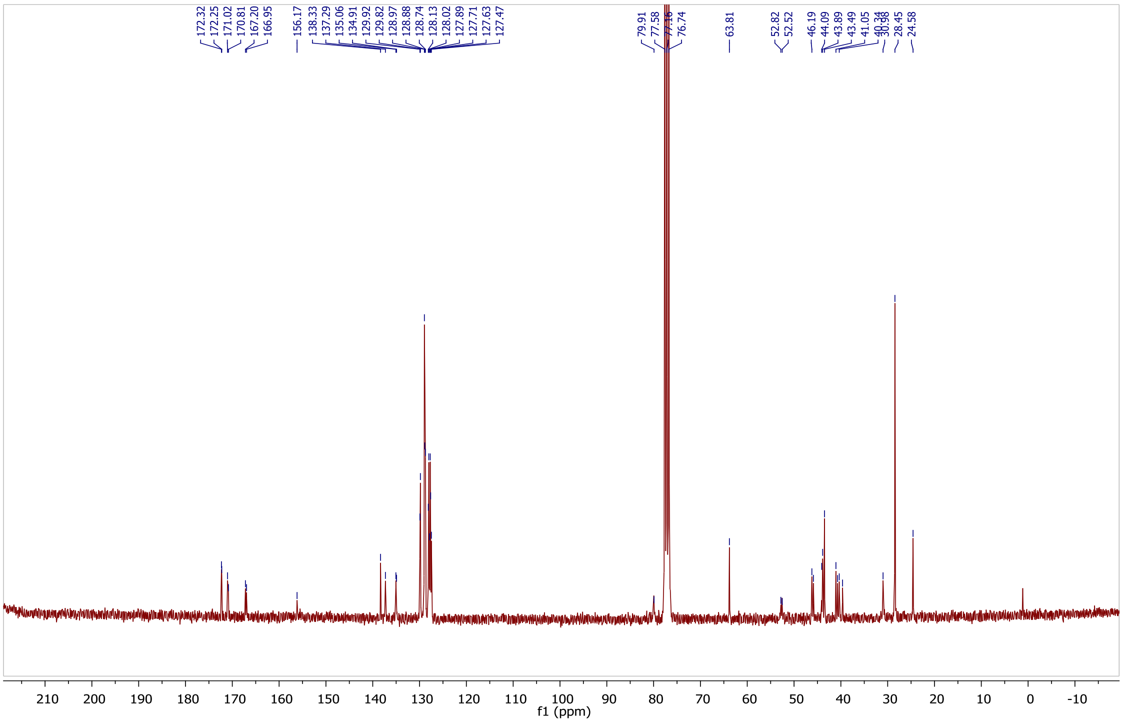

^{1}H-NMR (400 MHz, DMSO-d$_6$) and ^{13}C-NMR (75 MHz, DMSO-d$_6$) (65a)

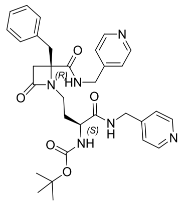

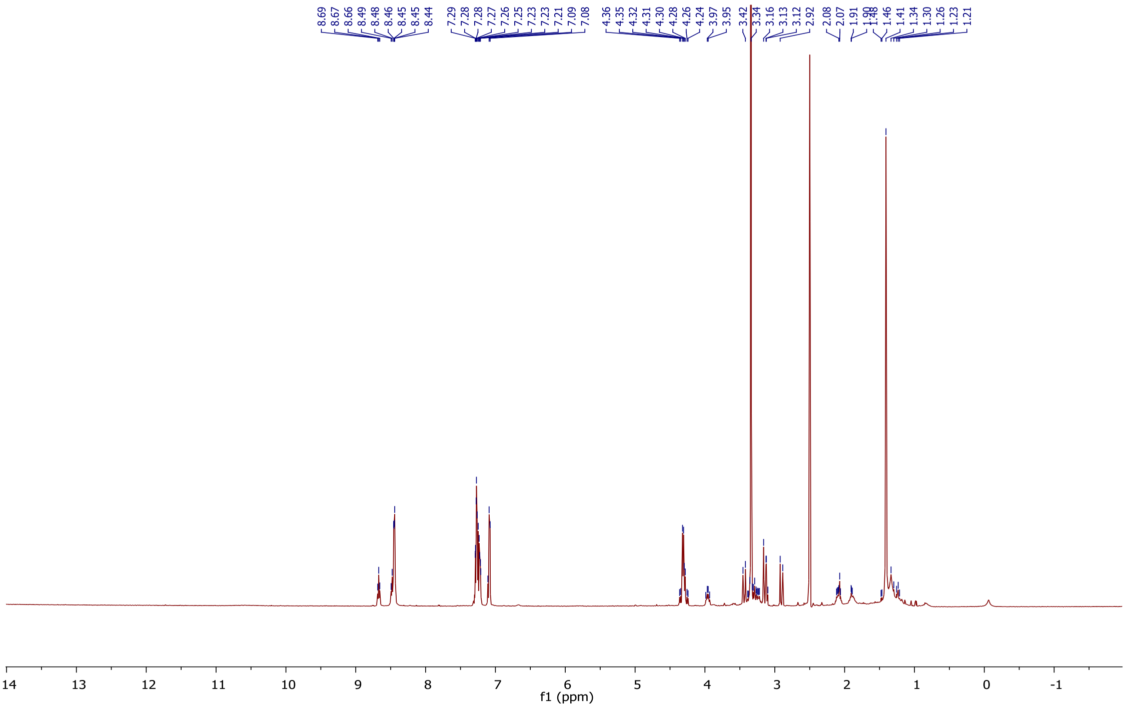

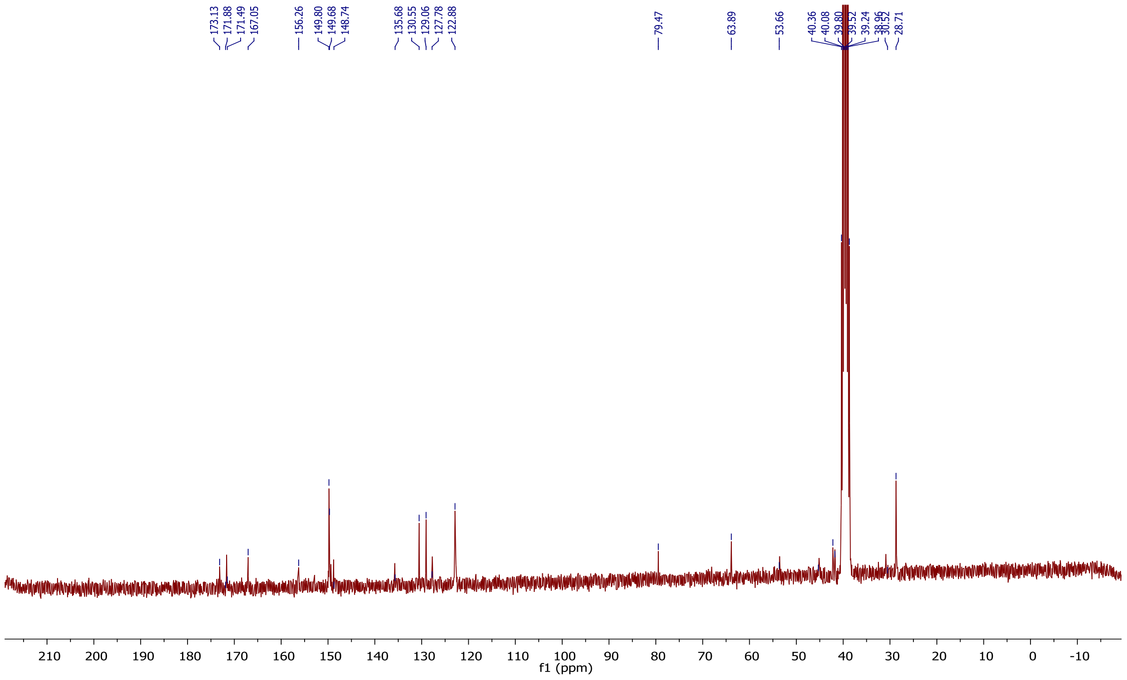

^{1}H-NMR (400 MHz, $CDCl_3$) and ^{13}C-NMR (75 MHz, $CDCl_3$) (65b)

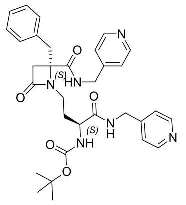

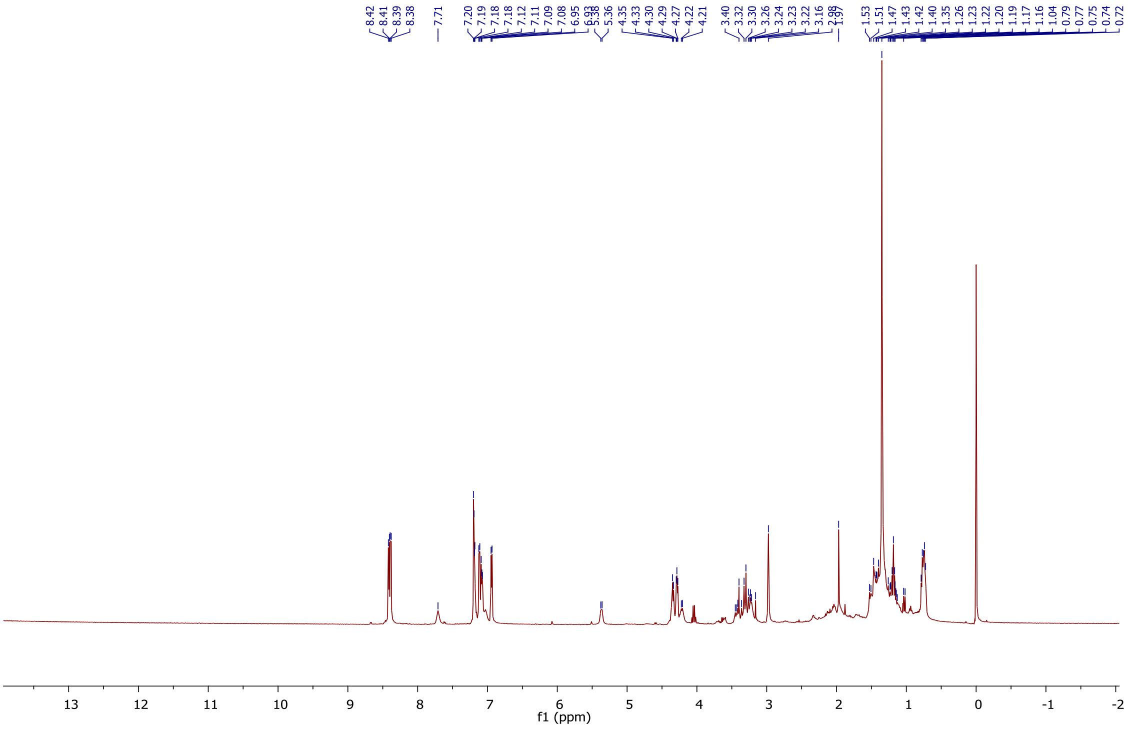

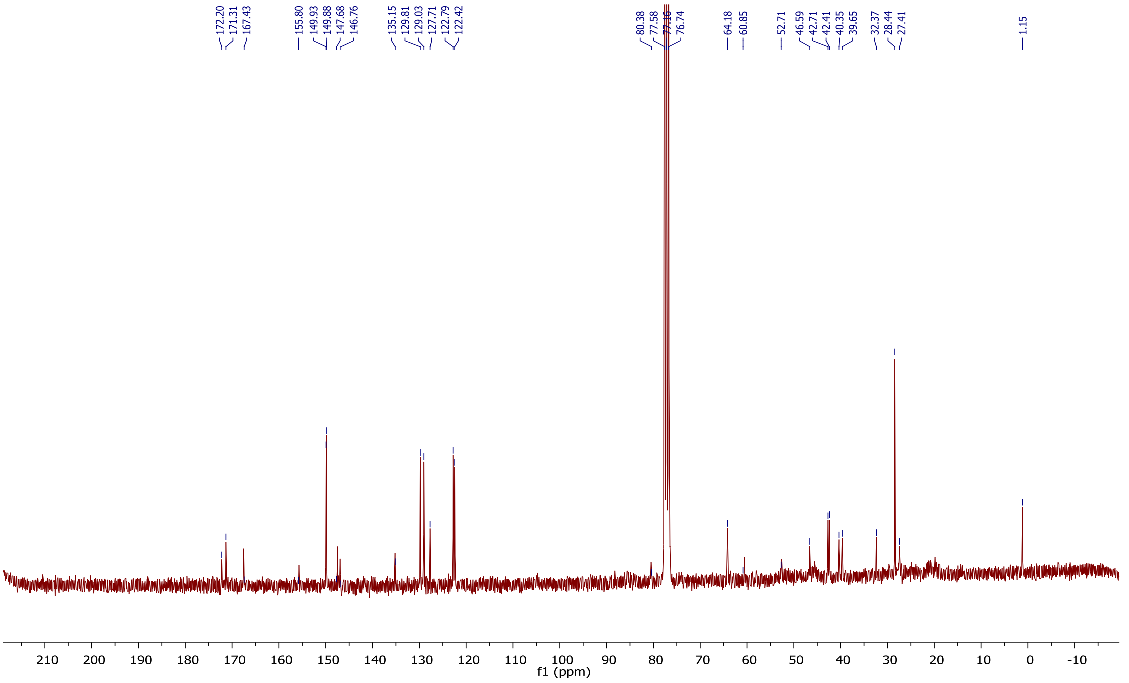

^{1}H-NMR (400 MHz, $CDCl_3$) and ^{13}C-NMR (75 MHz, $CDCl_3$) (66)

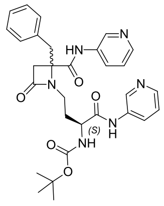

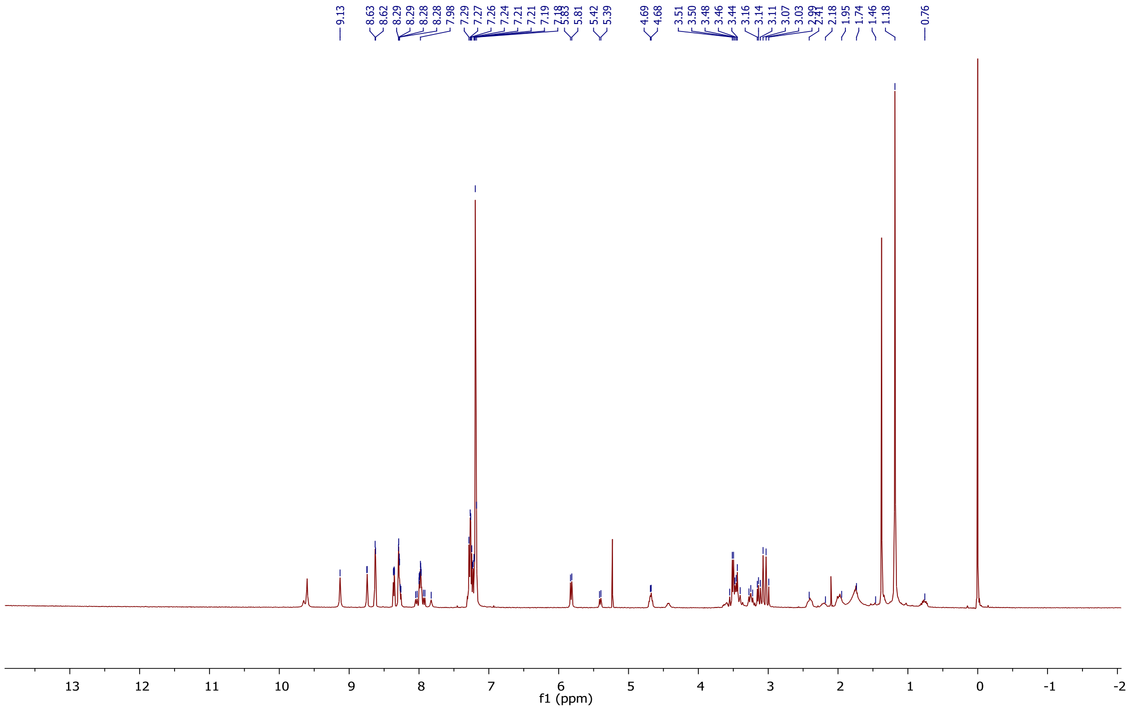

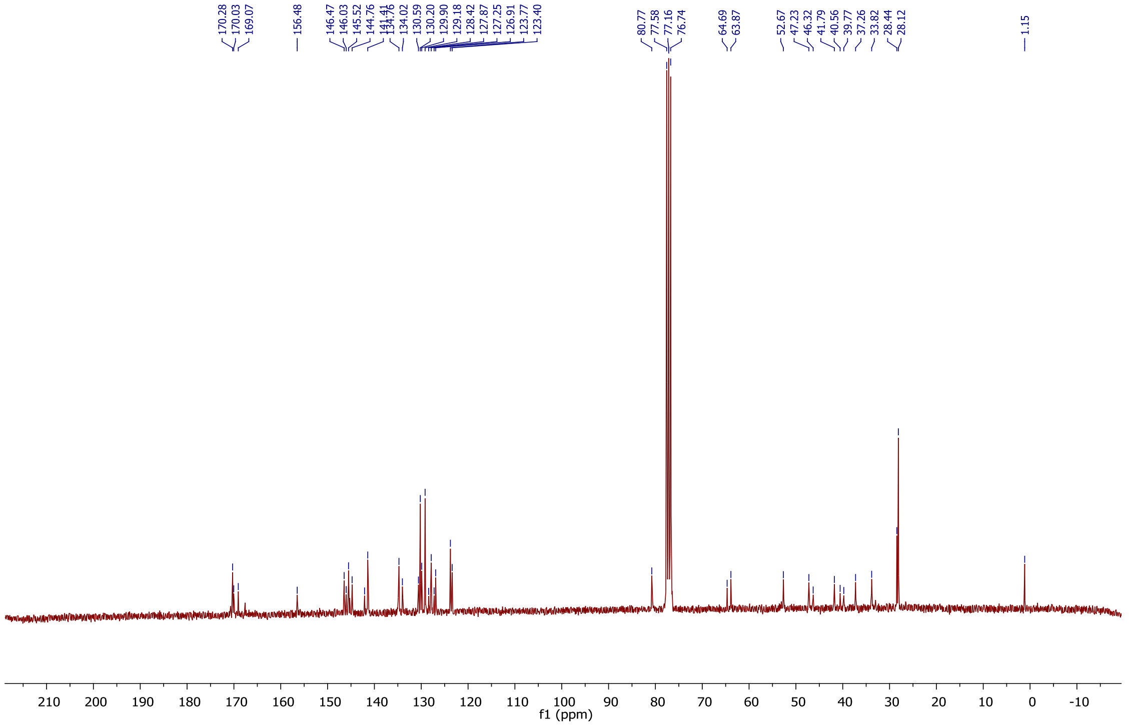

^{1}H-NMR (400 MHz, DMSO-d_6) and ^{13}C-NMR (75 MHz, CD_3OD) (67a)

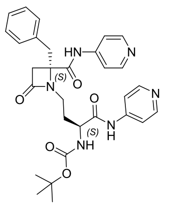

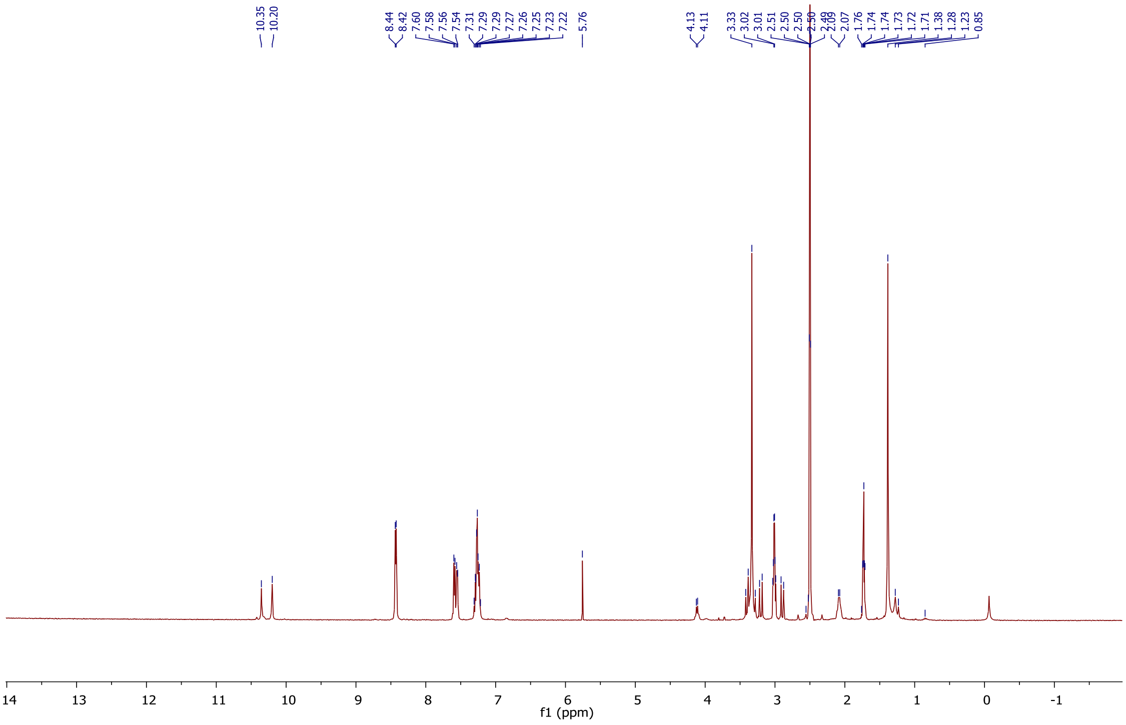

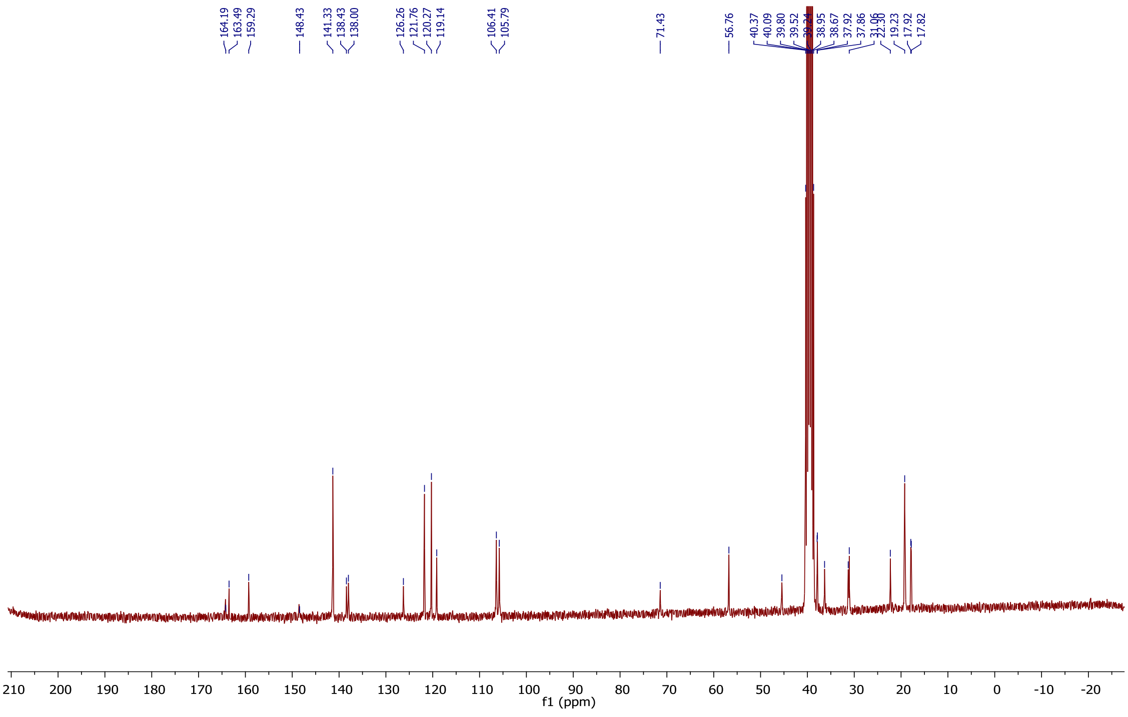

^{1}H-NMR (400 MHz, DMSO-d_6) and ^{13}C-NMR (75 MHz, CD_3OD) (67b)

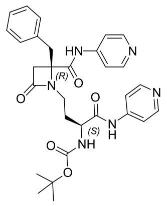

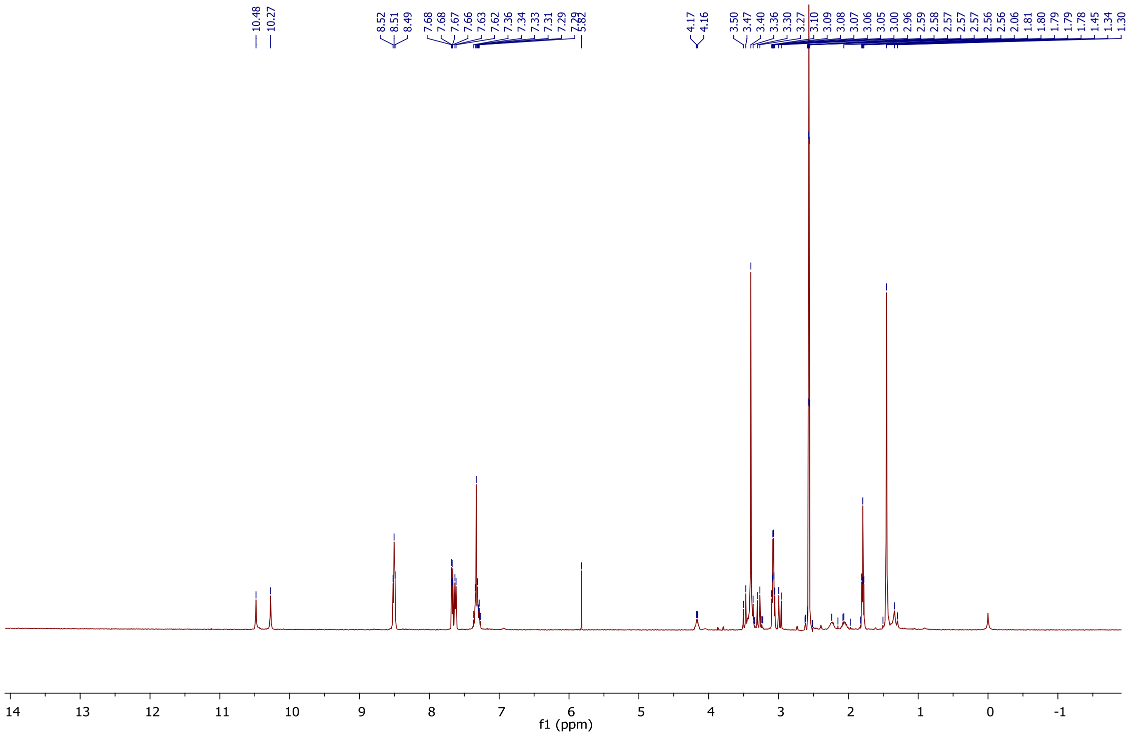

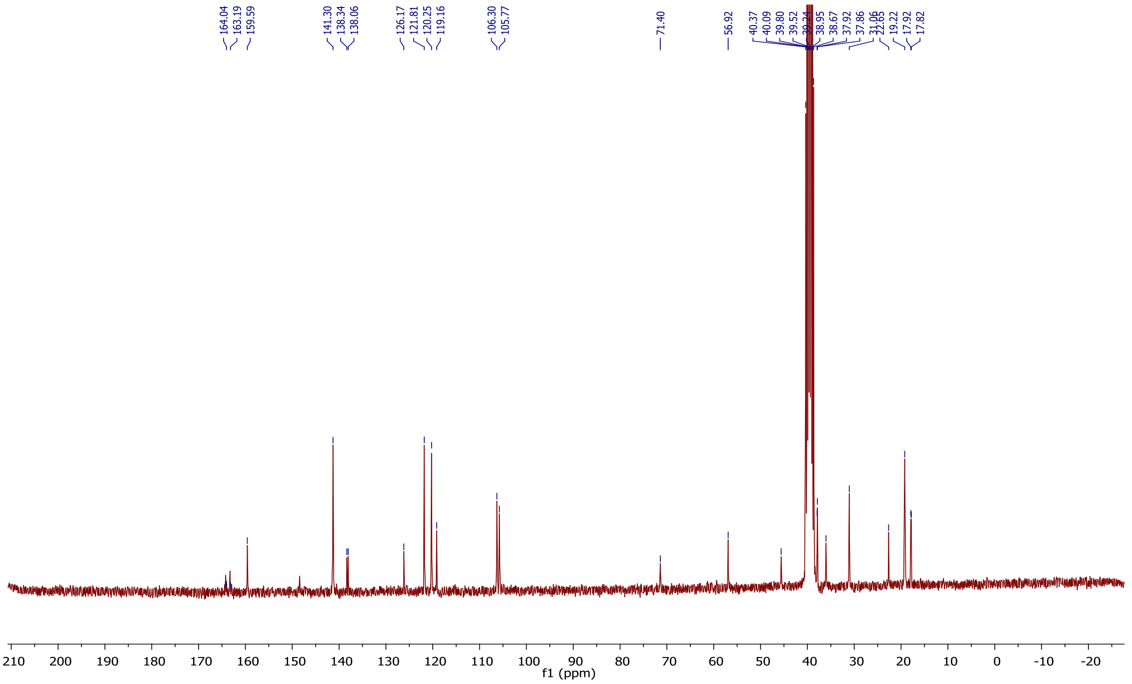

Supplement: Supplementary file 1 — Supporting Data [file 41598_2017_10913_MOESM1_ESM.pdf]
